# Polyester nanoparticles delivering chemotherapeutics: Learning from the past and looking to the future to enhance their clinical impact in tumor therapy

**DOI:** 10.1002/wnan.1990

**Published:** 2024-09-01

**Authors:** Giuseppe Longobardi, Thomas Lee Moore, Claudia Conte, Francesca Ungaro, Ronit Satchi‐Fainaro, Fabiana Quaglia

**Affiliations:** ^1^ Department of Pharmacy University of Naples Federico II Naples Italy; ^2^ Department of Physiology and Pharmacology, Faculty of Medicine Tel Aviv University Tel Aviv Israel; ^3^ Sagol School of Neurosciences Tel Aviv University Tel Aviv Israel

**Keywords:** cancer nanomedicine, nanoparticles, PLGA, polyester

## Abstract

Polymeric nanoparticles (NPs), specifically those comprised of biodegradable and biocompatible polyesters, have been heralded as a game‐changing drug delivery platform. In fact, poly(α‐hydroxy acids) such as polylactide (PLA), poly(lactide‐*co*‐glycolide) (PLGA), and poly(ε‐caprolactone) (PCL) have been heavily researched in the past three decades as the material basis of polymeric NPs for drug delivery applications. As materials, these polymers have found success in resorbable sutures, biodegradable implants, and even monolithic, biodegradable platforms for sustained release of therapeutics (e.g., proteins and small molecules) and diagnostics. Few fields have gained more attention in drug delivery through polymeric NPs than cancer therapy. However, the clinical translational of polymeric nanomedicines for treating solid tumors has not been congruent with the fervor or funding in this particular field of research. Here, we attempt to provide a comprehensive snapshot of polyester NPs in the context of chemotherapeutic delivery. This includes a preliminary exploration of the polymeric nanomedicine in the cancer research space. We examine the various processes for producing polyester NPs, including methods for surface‐functionalization, and related challenges. After a detailed overview of the multiple factors involved with the delivery of NPs to solid tumors, the crosstalk between particle design and interactions with biological systems is discussed. Finally, we report state‐of‐the‐art approaches toward effective delivery of NPs to tumors, aiming at identifying new research areas and re‐evaluating the reasons why some research avenues have underdelivered. We hope our effort will contribute to a better understanding of the gap to fill and delineate the future research work needed to bring polyester‐based NPs closer to clinical application.

This article is categorized under:

Therapeutic Approaches and Drug Discovery > Nanomedicine for Oncologic Disease
Nanotechnology Approaches to Biology > Nanoscale Systems in Biology
Therapeutic Approaches and Drug Discovery > Emerging Technologies

## Graphical abstract

An overview of key factors in the clinical translation of nanoparticle (NPs) technologies, from composition, scalable production and biological identity through in vitro and in vivo testing, to personalized medicine and patient selection.

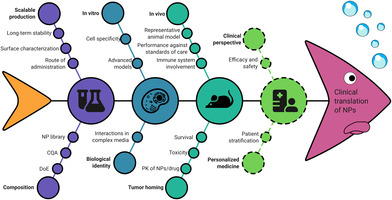

## 1 INTRODUCTION

Nanomedicines represent the forefront of today's therapeutics, and their application in oncology has steadily increased in the last 30 years (Salvioni et al., 2019). By providing a protective housing for the drug, nanomedicines promise to enhance the therapeutic outcomes of conventional chemotherapies, reduce their toxicity, treat metastasis, and overcome multidrug resistance (Iyer et al., 2013; Jain, 2010; Markman et al., 2013; Mitchell et al., 2021; Peer et al., 2007; Pozzi et al., 2021; Satchi‐Fainaro et al., 2004; Schroeder et al., 2012; Su et al., 2021; Wang et al., 2012). The advances in RNA‐based drugs and gene therapies and the increasing understanding of cancer biology open new avenues to rethink nanocarrier design and applications. The availability of a wide range of materials and the advances in production technologies now enable precise tailoring of nanocarrier properties relevant to the delivery of chemotherapeutics. In parallel, the elucidation of nanocarrier behavior at nano‐biointerface (e.g., biomolecular corona formation; Caracciolo et al., 2017; Lazarovits et al., 2015), and an increasing understanding of the link between their chemical (or synthetic) identity and dynamic biological identity is expected to provide effective tools for controlling their biodistribution in the body.

Polymeric nanoparticles (NPs) have emerged as a versatile and promising drug delivery system for cancer therapy due to their favorable properties such as biocompatibility, tunable physicochemical properties, protection from in vivo degradation, controlled release of therapeutic payload, and ease of surface modification for targeted delivery of therapeutics (Markovsky et al., 2012; Nishiyama & Kataoka, 2006; Pérez‐Herrero & Fernández‐Medarde, 2015). The dramatic advances in synthesizing polymers with diverse topologies and architectures, combined with controlled living polymerization methods and click reactions, provide almost infinite opportunities to build complex macromolecules (Roka et al., 2022). In parallel, by devising supramolecular assembly, core‐shell nanoconstructs, where the core primarily hosts the therapeutic agent and the shell acts as the functional interface with the biological environment, open new possibilities for designing multifunctional nanoplatforms with various aspect ratios and responsive to internal and external stimuli (Deng et al., 2012; Fleige et al., 2012; Hadinoto et al., 2013; Zhang, Chen, et al., 2020; Zhong et al., 2014).

In the large class of biocompatible and biodegradable polymers, aliphatic polyesters have been considered the preferred choice in the development of NPs for the delivery of chemotherapeutics for decades (Acharya et al., 2009; Danhier et al., 2012), and more recently, as nano‐vaccines for infectious diseases and cancer (Florindo et al., 2020; Peres et al., 2021). Poly(lactide‐*co*‐glycolide) (PLGA) is the most widely known and primarily employed poly(α‐hydroxy acid) in the drug delivery field. PLGA has been successfully used to deliver peptides at sustained rates and protect the therapeutic cargo from enzymatic degradation (Park et al., 2019). Several injectable medicines based on PLGA microparticles were introduced in the early 1990s and remain valid therapeutic options for certain cancers (Lupron Depot®, Sandostatin® LAR, Trelstar®, Somatuline® Depot, Arestin®, Bydureon®, Lupaneta Pack™, Signifor® LAR). Polylactide (PLA) and poly(є‐caprolactone) (PCL) are primarily used in tissue engineering scaffolds, implantable medical devices, sutures, and surgical meshes.

Amphiphilic diblock copolymers of PLGA, PLA, and PCL with poly(ethylene glycol) (PEG) open tremendous opportunities in the development of a variety of NPs with different sizes, shapes, surface‐to‐volume ratios, and functional surfaces (Chatterjee & Chanda, 2022; Danhier et al., 2012; Dash & Konkimalla, 2012; Dinarvand et al., 2011; Grossen et al., 2017; Lakkireddy & Bazile, 2016; Makadia & Siegel, 2011; Swider et al., 2018; Zhang et al., 2014).

Despite the existing solutions and research efforts in developing therapeutic polyester NPs, their full potential has not been realized yet (Hare et al., 2017; Hua et al., 2018; Luxenhofer et al., 2014). Only a limited number of clinical trials have been conducted, and the outcomes have not been consistently encouraging. Polyester NPs delivering paclitaxel have been evaluated in clinical trials up to Phase II in the case of BIND‐014 and reached the market only for Genexol®‐PM (Box 1) Polyester NPs currently undergoing evaluation in clinical trials are reported in Table 1. These studies are predominantly focused on PLGA, and are not confined to oncological applications alone but extend to a diverse array of medical conditions. This raises the question of why it is so challenging to develop such systems and how to overcome their shortcomings. As for many nanopharmaceuticals, the mode of action of polyester NPs remains elusive, and novel delivery concepts are often confined to preclinical studies. Furthermore, their technological development is complex since robust prototyping at the bench frequently underestimates regulatory constraints and scale‐up manufacturing (Operti et al., 2021).

BOX 1 **The battle of “nano” paclitaxel**

This is the title of a paper reporting the chronology of nano‐paclitaxel development was told (Sofias et al., 2017) and to which we add the finale. Abraxane, an albumin‐bound paclitaxel manufactured by Abraxis Bioscience, was approved by the Food and Drug Administration (FDA) and the European Medicines Agency (EMA) in 2005 and 2008, respectively, as second‐line therapy for metastatic breast cancer patients with resistance to an anthracycline plus taxane regimen. Abraxane increased the maximum tolerated dose (MTD) compared to the Cremophor‐EL formulation (Gradishar et al., 2005). In 2010, Celgene Corporation acquired Abraxane/Abraxis Bioscience for $2.9 billion (http://ir.celgene.com/releasedetail.cfm?releaseid=799482). The results of a phase III study led to enlarge the indications of metastatic pancreatic adenocarcinoma in 2012 increasing sales. In the same time window, Genexol®‐PM (Cynviloq™), an injectable micelle formulation of paclitaxel based on PEG–PLA (2 and 1.75 kDa, respectively) originally developed by Samyang Biopharmaceuticals Corporation, gained the first clinical approval in South Korea in 2006 with the indications of a first‐line therapy for recurrent and metastatic breast cancer and nonsmall‐cell lung cancer (NSCLC) in combination with cisplatin (Lee et al., 2017). In 2013, the product was acquired by Sorrento Therapeutics after merging with IgDraSol obtaining exclusive distribution rights in North America, the European Union, Australia, and South America. A bioequivalence trial (TRIBECA™) was designed to establish the bioequivalence between Cynviloq™ and the blockbuster drug Abraxane™. The initial results of the study demonstrated bioequivalence for the two formulations (http://www.prnewswire.com/news‐releases/initial‐pharmacokinetic‐data‐from‐tribeca‐study‐support‐potential‐for‐bioequivalence‐between‐cynviloq‐and‐albumin‐bound‐paclitaxel‐279181641.html) making Cynviloq™ a possible Abraxane® competitor. Cynviloq™ allowed treatment in the range of 300–435 mg/m^2^ in comparison to Abraxane which had an MTD of 300 mg/m^2^ (Yamada et al., 2010) (https://sorrentotherapeutics.com/wp‐content/uploads/2013/12/Sorrento_Corporate_Presentation_December_2013.pdf). Meanwhile, a Phase III clinical trial (NCT00876486) conducted in South Korea demonstrated superior clinical efficacy of Cynviloq™ in comparison to a conventional Cremophor‐EL based PTX formulation in patients with HER2‐negative advanced or metastatic invasive breast cancer, likely, due to the higher allowed dose (i.e., 260 mg/m^2^ and 175 mg/m^2^ for Cynviloq™ and the conventional paclitaxel formulation, respectively) (Park et al., 2017).

After the first results of TRIBECA™, in 2015 NantPharma acquired Igdrasol, the subsidiary of Sorrento, to acquire the rights of Cynviloq™ in a deal worth $1.3 billion. Sorrento had the option to co‐develop and co‐market Cynviloq™ (https://www.pharmaceutical‐technology.com/news/newsnantpharma‐buy‐sorrento‐igdrasol‐4,579,487/?cf‐view). Nantpharma was a drug developer founded by the same founder of Abraxis BioScience, which is dedicated to the development of the core technologies behind albumin‐bound paclitaxel. In 2019, Sorrento Therapeutics filed an arbitration demand against NantPharma, alleging that the company had failed to live up to its contractual obligations to develop Cynviloq™. In March 2023, the LA County Superior Court confirmed the arbitration award of $125 million in damages to be paid by NantPharma established on December 2022. The doubt arises on how much financial reasons impacted the approval of Cynviloq™.

**TABLE 1:** PLGA‐based nanomedicines in ongoing clinical trials.

Nanoparticles type	API	Disease	Stage	Description	Clinical trial ID number	Sponsor	Source
PLGA–PEG	Quercetin	Tongue squamous cell carcinoma	Phase II	Quercetin‐encapsulated nanoparticles for targeting and treating tongue squamous cell carcinoma.	NCT05456022	Cairo University	www.clinicaltrials.gov/study/NCT05456022
PLGA	PRECIOUS‐01	New York esophageal squamous cell carcinoma‐1 (NY‐ESO‐1) positive cancers	Phase I	PRECIOUS‐01 is an immunomodulating agent composed of the invariant natural killer T cell (iNKT) activator threitolceramide‐6 (ThrCer6, IMM60) and NY‐ESO‐1 cancer‐testis antigen peptide encapsulated in PLGA NPs.	NCT04751786	Radboud University Medical Center	(Creemers et al., 2021)
PLA–PLGA	Ciprofloxacin hydrochloride	Antibiotic‐resistant infections	Early Phase I	NPs containing antibiotics incorporated in in situ gel to treat recurrent endodontic infections.	NCT05442736	British University, Egypt	(Fereig et al., 2020)
PLGA	CNP‐104	Primary biliary cholangitis	Phase II	CNP‐104 is composed of PDC‐E2 peptide dispersed within a negatively charged polymer matrix of PLGA particles to treat primary biliary cholangitis.	NCT05104853	COUR Pharmaceutical Development Company, Inc.	www.clinicaltrials.gov/study/NCT05104853
PLGA	CNP‐106	Myasthenia gravis	Phase II	CNP‐106 is comprised of an antigenic AChR peptide pool dispersed within a negatively charged polymer matrix of PLGA particles (400–800 nm in size).	NCT06106672	COUR Pharmaceutical Development Company, Inc.	www.clinicaltrials.gov/study/NCT06106672
PLGA	Ciprofloxacin	Bacterial infections	–	Chitosan‐coated PLGA NPs loaded with ciprofloxacin, incorporated in smart gel for localized infection treatment	NCT05475444	British University, Egypt	https://clinicaltrials.gov/study/NCT05475444

This review provides an overview of the results achieved so far on polyester NPs engineered explicitly for delivering chemotherapeutics to solid tumors. First, we will give a snapshot of the relevance of polyester NPs in cancer research. Then, we describe the main critical aspects in their formulation and fabrication, pointing at critical attributes expected to drive biological behavior. After discussing the main barriers hampering tumor homing of NPs, we critically revise the engineering strategies adopted thus far for targeting solid tumors upon intravenous administration, focusing on the link between NP physicochemical properties and their interaction with the biological milieu. Finally, we report the latest advances in the design of polyester NPs and opportunities for alternative administration routes.

## 2 KNOWLEDGE MAPPING OF BIODEGRADABLE NANOPARTICLES DELIVERING CHEMOTHERAPEUTICS

To better understand the landscape around PLGA, PLA and PCL NPs for the treatment of cancer, a science mapping approach was employed via Clarivate™ Web of Science™ (WoS). A search of several combined topics was conducted to find documents related to polyester NPs delivering chemotherapeutics (https://www.webofscience.com/wos/woscc/summary/3041a176-28c5-4a54-8f22-a2c8822f2bfb-cc22902a/relevance/1, Accessed 12 February 2024), and search results were filtered only to include “Articles” and “Reviews” as the document type. These results were further restricted to documents in the English language. The final result was a database comprising 3964 articles (2764 original research articles and 604 reviews) spanning 1998 to 2024. The methodology employed and a further breakdown of the manuscripts, journals, and citations can be found in the Supporting Information.

**FIGURE 1:**
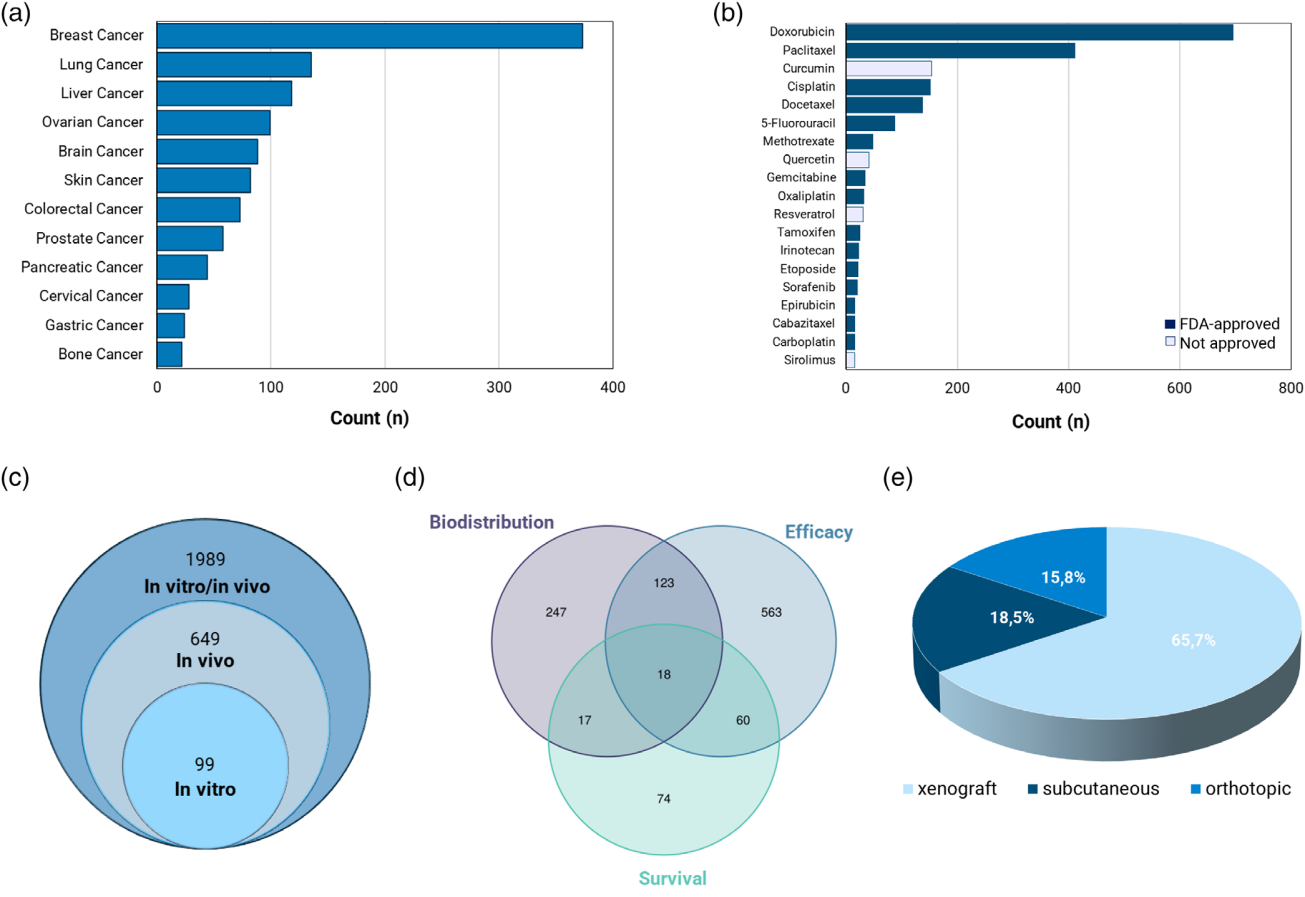
(a) An analysis of the “cancer types” referenced in the abstracts of the curated scientific articles (excluding reviews) shows the most prevalent cancers investigated in the polymeric nanomedicine domain. (b) A search through the abstracts of these scientific articles identified the most commonly referenced anti‐cancer therapeutics, broken down by whether those therapeutics have received FDA approval for the treatment of cancer. (c) Search and comparison of keyword groups within the abstracts of scientific articles showing the overlap between in vitro/in vivo studies, as well as, (d) within in vivo studies, the overlap of studies examining biodistribution, efficacy, and survival (and all combinations thereof). (e) Breakdown of abstracts referencing “orthotopic”, “subcutaneous”, and “xenograft” tumors within the in vivo studies.

To gain an overview of the most investigated types of cancers, a search was done through the abstracts of each article for cancer‐related words contained within a list (Supporting Information). These words were either types of cancers or cancers with the roots of blastoma, carcinoma, or sarcoma. Specific types of cancer were then grouped (e.g., “breast cancer”, “breast carcinoma”, and “mammary carcinoma” were grouped into “breast cancer”). From these results, it was possible to identify the types of cancers that appeared in at least 20 articles (Figure 1a), with breast cancer being the most investigated.

Similarly, a search was done for the top therapeutics delivered through polyester NPs. Figure 1b shows the top 20 drugs investigated in polymeric nanomedicine for treating cancer. It is clear that doxorubicin, by a large margin, is the most studied therapeutic—one could speculate that this is due to the “early” approval of Doxil® and the large body of work coming from this milestone as well as its easy fluorescence detection. More interesting is that several compounds listed (e.g., curcumin, quercetin, and resveratrol), while heavily investigated in the context of polymeric nanomedicine, are not yet approved in any form for treating cancer. A search of these compounds with “cancer” in the U.S. Clinical Trials database (www.clinicaltrials.gov) returned 87, 19, and 19 studies for curcumin, quercetin, and resveratrol, respectively. Another drug, sirolimus, has received FDA approval as a stent coating for preventing organ transplant rejection but is not currently approved for cancer treatment (although it is in clinical trials).

One goal of the literature analysis was to investigate the relationships between certain keywords and their overlap within the abstracts of the manuscripts. A search was conducted to construct a list of “in vivo” manuscripts, comprised of abstracts that contained the words “in vivo”, “animal(s)”, “mouse”, “mice”, or “pig(s)”. Likewise, a search was conducted for “in vitro” manuscripts containing the words “cell(s)”, “cell line(s)”, and “in vitro”. Then, using the digital object identifier (doi) number as a unique identifier, results were filtered and compared to find those studies that referenced either “in vitro” or “in vivo” work (Figure 1c). In a similar analysis, the so‐called “in vivo” database was further dissected to evaluate the overlap of studies that investigated “efficacy”, “survival”, and “biodistribution” OR “pharmacokinetics.” Of course, these are coarse evaluations that cannot fully or deeply investigate the merits of each study but provide a means to understand, at a glance, exactly how comprehensive the studies are (i.e., are in vivo studies that investigate the efficacy of some nanomedicine also reporting the survival). What is not possible with this type of analysis is to understand if future works follow up on certain aspects. That is, does a study investigating the biodistribution of particles within a tumor model follow with a study on nanomedicine efficacy and animal survival. There exists a large discrepancy between those articles referencing “efficacy” within the abstract (563 articles) versus those explicitly referencing “survival” (74 articles) or “survival and efficacy” (60 articles). This could be due to a lack of precision in delineating the two terms (e.g., considering survival a characteristic of an efficacious treatment). It could also result from the imprecision of such an approach, which relies on the explicit mention of words or word roots within the abstract and does not allow for a looser interpretation of the data contained within the manuscripts.

A second search was done within the “in vivo” database to understand the breakdown of types of in vivo tumor models: “orthotopic”, “subcutaneous”, or tumor “xenografts” (Figure 1d). A search of these three roots within the abstracts of all the “in vivo” scientific articles (excluding reviews) surprisingly yielded only 268 manuscripts of the 2638 manuscripts in the original “in vivo” database, that is, roughly 10%. This could be interpreted in several ways. One being that there is an abundance of studies within the in vivo subset that pertain solely to studies of nontumor biodistribution or nontumor toxicity of the NPs. This will certainly be the case for some studies. However, it is more likely that vague terminology is used within the abstract, such as referring only to “tumor models” or, for example, “MCF‐7 tumors” to cite a specific cell line. In the latter case, this terminology would give a clear idea of what kind of cancer is being investigated but lacks more detailed information on the “type” of tumor model (e.g., tumor xenograft, mammary fat pad model, subcutaneous tumor model, etc.).

These data reflect an interesting approach in which snapshots can be taken of a large number of manuscripts to provide details as to the types of research and some broad ideas about the research workflow within a field (or subfield). Moreover, this approach reflects a necessity to provide precise information within the abstract and keywords of articles to obtain reliable data in knowledge mapping.

## 3 PREPARATION OF POLYESTER NANOPARTICLES FOR THE DELIVERY OF CHEMOTHERAPEUTICS: AN OVERVIEW

NPs made of unmodified polyesters are not well suited for delivering chemotherapeutics due to poor colloidal stability and immune recognition, which limit their capacity to deliver drug cargo at the tumor level. Core‐shell NPs, with a hydrophilic shell surrounding the lipophilic polyester core, fit much better for this purpose. The shell promotes “in the bottle” stability during fabrication and storage and, if appropriately tailored, guides nano‐biointerface interactions.

Devising core–shell NPs involves selecting the polymer type (chemistry and architecture) and implementing fabrication conditions to attain the appropriate topological distribution of surface functional elements and drug payload. Three main strategies can be adopted for this purpose: (i) the non‐covalent adsorption of shell components on the pristine polyester nanocore; (ii) assembly of chemically modified amphiphilic polyesters; (iii) covalent attachment of specific moieties on the surface of a polyester nanocore (post‐modification).

The optimization of polyester NP properties in terms of size, surface features, drug loading extent, drug release rate, and shelf‐life is strictly related to the selection of appropriate excipients and pertinent fabrication conditions (Hernández‐Giottonini et al., 2020). Unfortunately, this is a case‐by‐case process in which physical–chemical features of the Active Pharmaceutical Ingredient (API; aqueous solubility, stability, and compatibility with excipients) play a major role. Readers are addressed to reviews reporting the fabrication of PLGA NPs (Operti et al., 2021; Swider et al., 2018) and PCL NPs (Grossen et al., 2017) and highlighting the main challenges in their industrial scale‐up (Operti et al., 2021). An overview of formulation approaches for polyester NPs and indications on how to solve the difficulties in the development path has been recently provided (Molavi et al., 2020).

Here, we recall polyester features relevant to NP preparation and summarize preparation methods to generate core‐shell NPs tailored for the delivery of chemotherapeutics. Furthermore, we highlight some poorly addressed aspects in pharmaceutical development that need urgent solutions to provide robust prototypes for advanced preclinical and clinical studies.

### 3.1 Properties of polyesters relevant to nanoparticle design

**FIGURE 2:**
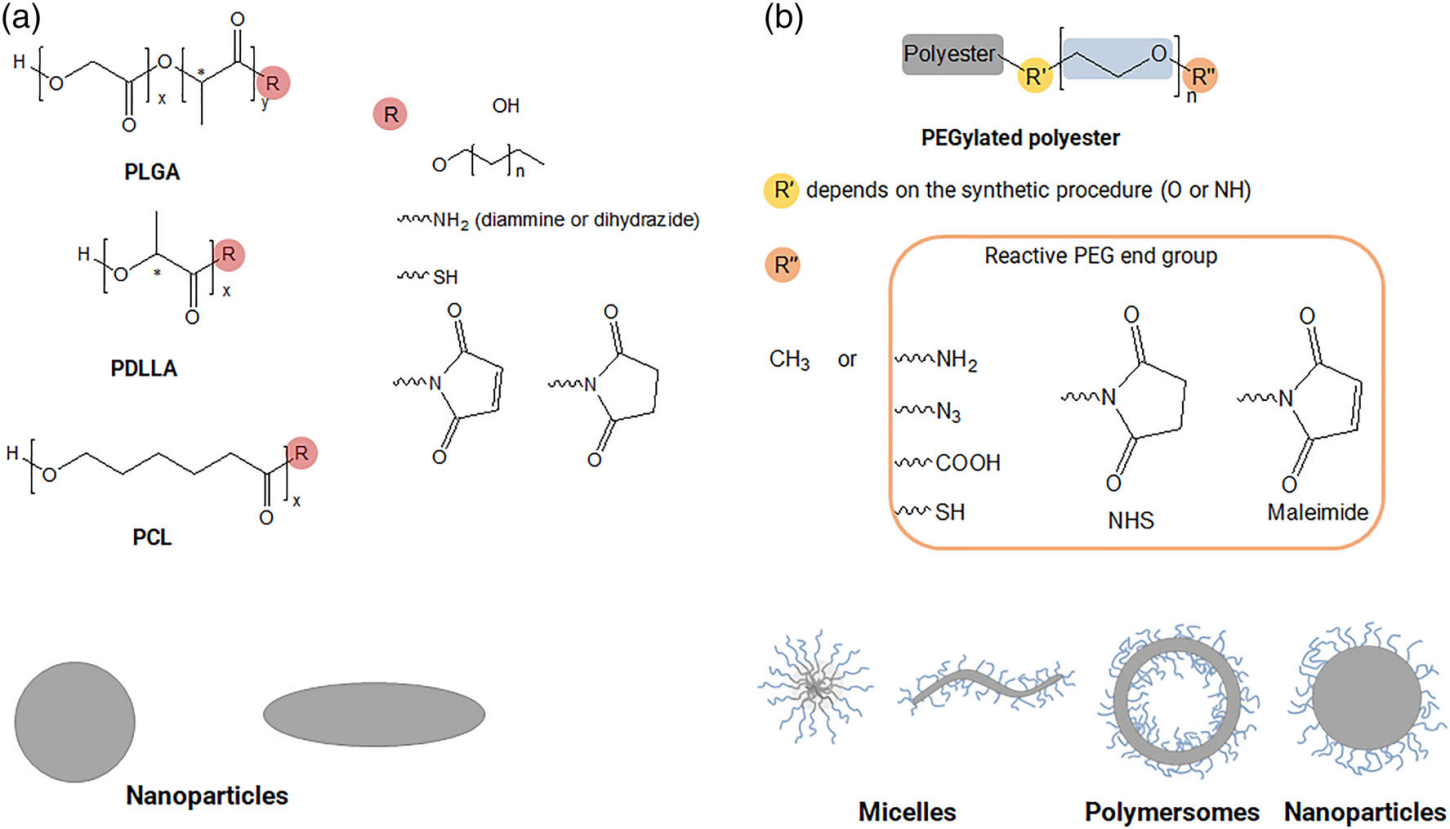
Chemical structure of polyesters (a) and PEGylated polyesters (b) and corresponding type of nanocarriers that can be obtained. In (a), R = OH for uncapped polyesters. In (b), the chemical bond between the polyester and PEG R′) can be an ester or an amide bond. PEGylated polyesters bear a –CH_3_ at PEG end or a variety of reactive groups for further modification (R″).

The structure of the polyesters most widely employed to prepare NPs are reported in Figure 2a. (PLA and PLGA) and poly(glycolide) acid (PGA) are members of the poly(α‐hydroxy acid) family with repeating lactic acid and lactic acid/glycolic acid units, respectively.

PLA has three stereoisomers: poly(l‐lactide) (PLLA), poly(d‐lactide) (PDLA), and poly(d,l‐lactide) (PDLLA). PLLA shares similar properties to PDLA and exhibits relatively high crystallinity, while PDLLA is amorphous. PLLA has been preferred in biomedical applications since the l‐lactic monomer is involved in cellular metabolism (Sheikh et al., 2015). All PLA are soluble in acetonitrile and methylene chloride, while they are only partially soluble in acetone and tetrahydrofuran. Crystalline PLLA cannot be dissolved in acetone, ethyl acetate, or tetrahydrofuran. Its glass transition temperatures are in the range of 45–65°C.

PLGA is another member of this family made of lactic and glycolic acid repeating units with an ordered or random structure. PLGA NPs can be prepared by a range of commercial polymer variants with different compositions (L:G ratios 50/50, 65/35, 75/25, and 85/15), MW (ranging from 10 to >100 kDa), and end‐group chemistry (capped with alkyl moieties or uncapped). Branched PLGAs with a star‐shaped structure centered on a glucose core are available but rarely employed to produce NPs. PLGAs are not soluble in water, but they are soluble in organic solvents. PLGA with a higher amount of lactic acid is dissolved by chlorinated solvents, such as dichloromethane or chloroform, or ethyl acetate, and by water‐miscible solvents, like acetone or tetrahydrofuran (Uhrich et al., 1999). The solubility profile is strictly related to polymer MW and composition, with the L:G ratio being the main determinant of polymer solubility. In general, L:G ratios higher than 50:50 make the polymer soluble in volatile and nonvolatile solvents (Garner et al., 2021; Park et al., 2019) but also in glacial acetic acid, providing the possibility to generate nanostructures in a wide range of experimental conditions. PLGA copolymers feature a glass transition temperature (*T*_g_) range from 45 to 55°C, above the physiological temperature (37°C).

PLA and PLGA are chemically degraded in the body to oligomers and monomers that are removed from the body by renal excretion of unmodified lactic and glycolic acids, and respiration (Kreb's cycle with the formation of carbon dioxide and water), which accounts for their excellent biocompatibility.

Amid poly(ω‐hydroxyalkanoate), PCL is a semicrystalline homopolymer available in a large MW range, soluble in chlorinated solvents or tetrahydrofuran, and partially soluble in class 3 solvents like acetone and ethyl acetate (Zhang et al., 2019). Chemical hydrolysis is again the dominant degradation mechanism.

In both cases, an additional contribution to chemical degradation in vivo is due to enzymatic hydrolysis by esterases and lipases (Almeida et al., 2019).

The biodegradability of polyesters depends on polymer composition, crystallinity, *T*_g_, MW, device geometry, pH, and production conditions (Alexis, 2005; Makadia & Siegel, 2011). Overall, the fastest degradation is for amorphous 50/50 L/G PLGA copolymers, un‐capped derivatives, and lower MW as evaluated on microspheres or implants taking ca. 1–2 months to resorb (Engineer et al., 2011; Gentile et al., 2014). PLGA oligomers become water‐soluble at ca. 1 kDa (Fredenberg et al., 2011). PCL is instead a slow‐degrading material (2–3 years for PCL membranes) that does not provoke acidification of the local environment (Shi et al., 2014). Depending on the degradation rate, a sustained release of an API incorporated in the polyester matrix in more or less extended time‐windows can thus be achieved (Birnbaum & Brannon‐Peppas, 2003). In general, PCL polymers provide a less controlled release rate with an initial drug burst (Niyom et al., 2019). Although few papers report the biodegradation behavior of polyester NPs, it is accepted that the PLGA NPs erosion pattern is triphasic (no erosion, fast erosion, and slow erosion) with the following loss of integrity (Jain et al., 2010).

Coupling polyesters with poly(ethylene glycol) (PEG) is nowadays considered the most acceptable option to produce stable dispersions of polyester NPs (Figure 2b). Since PEG is hydrophilic, hydrophobic polyester chains linked to PEG become amphiphilic and able to form nanostructures surrounded by a hydrophilic PEG shell, which is, in principle, helpful to increase the colloidal stability of NPs in aqueous media. PEGylated diblock copolymers with different molecular weights and hydrophilic/lipophilic balance are synthesized from monomethoxy‐PEG (mPEG) with different molecular weights (commonly 2–5 kDa) as macroinitiator of polyester chains. Alternatively, a preformed polyester chain can be coupled to PEG with suitable end groups. This means that the chemistry of the polyester‐PEG bond can be changed (see R′ in Figure 2), providing a variety of derivatives soluble in water or volatile solvents depending on overall polymer MW and PEG–polyester chain lengths. Unless otherwise specified, the mPEG‐terminated diblock copolymers of PLGA, PLA, and PCL are called PEG–PLGA, PEG–PLA, and PEG–PCL.

The chemistry of polyester and PEGylated polyesters has further expanded the opportunity to engineer the surface of NPs with different molecules (Chatterjee & Chanda, 2022). Advances in polymerization methods and post‐polymerization modifications to attach complementary functional pairs have expanded the opportunities for polyester modifications. Among various reactions, copper(I)‐catalyzed azide‐alkyne cycloaddition (CuAAC), Diels‐Alder cycloaddition, thiol‐ene/thiol‐yne, and Michael addition reactions have found wide applications in the field (Chatterjee & Chanda, 2022). The reactive moieties on the –OH end group (R″) commonly employed to link functional components covalently are illustrated in Figure 2.

### 3.2 Fabrication technologies

Polyester NPs can be obtained via bottom‐up techniques like emulsion–solvent evaporation after solubilizing the polymer in nonwater miscible solvents, nanoprecipitation, and dialysis, employing water‐miscible solvents. In general, the solubility profile of the polymer can be a limiting factor due to the need for toxic solvents rarely used in pharmaceutical manufacturing.

Dichloromethane is the most diffused solvent for PLA, PLGA, and PCL in the emulsion technique. As a class II solvent, residual dichloromethane in the product must be assessed and reduced as much as possible. Partially water‐miscible and less toxic solvents, such as ethyl acetate, can alternatively be employed for certain polymer types (Haque et al., 2018). The O/W single emulsion method is especially suited for lipophilic API and consists of preparing a drug–polymer solution that is then emulsified in an aqueous phase containing an emulsifier. The W1/O/W1 double emulsion method applies to water‐soluble API and involves the preparation of a primary W1/O emulsion (drug in W1 and polymer in O) that is added to an aqueous solution containing an emulsifier (W2). Emulsification is attained with high shear mixing or sonication to obtain tiny NPs. The solvent is then evaporated under stirring or dialyzed to solidify NPs. Controlling process parameters allows PLGA NPs to be synthesized over a broad size range with low polydispersity index (PDI) and high batch‐to‐batch reproducibility.

Nanoprecipitation (solvent displacement) is a one‐step process commonly employed since it is easy to realize without special equipment and the need for high energy or high shear (Fessi et al., 1989). In nanoprecipitation, the polymer is dissolved with the drug in a water‐miscible organic solvent or solvent mixture (such as acetone, acetonitrile, and tetrahydrofuran). The organic phase is then slowly added into an aqueous phase, and NPs are rapidly formed by the quick solvent diffusion and interfacial deposition of the polymer. Since many chemotherapeutics are poorly water soluble, this preparation technique is widely diffused in cancer nanomedicine production.

The preparation of relatively uniform and easily re‐dispersible spherical NPs often necessitates the incorporation of a stabilizer (poloxamers or polysorbates in nanoprecipitation; polyvinyl alcohol in emulsification methods) that remains adsorbed onto NP, contributing to the definition of NP chemical identity (Mundargi et al., 2008).

Generally, the costly large‐scale production, the lack of narrow and uniform size distributions of the particles, and the use of large amounts of organic solvent are critical in these processes. Since these technologies are used only for small batch preparations, scaling up the production can alter the formulation characteristics, which is a main drawback (Operti et al., 2021). The diffusion of microfluidic and millifluidic systems that can manipulate fluids within small channels (Hung et al., 2010) and the flash nanoprecipitation by confined impinging jet mixer (Johnson & Prud'homme, 2003) have represented a cornerstone in the large‐scale fabrication of polyester NPs. They offer the opportunity to parallelize many devices, making continuous synthesis and scale‐up possible for clinical studies (Caggiano et al., 2023; Liu, Yang, et al., 2022). In microfluidic production, different chip geometries and formulation components can be combined to achieve various sophisticated PLGA NPs architectures (Rezvantalab & Keshavarz Moraveji, 2019) with improved control over particle properties in comparison with bulk methods (Mares et al., 2021; Streck et al., 2019). These process technologies allow more precise control of the size and polydispersity of NPs, ensuring batch‐to‐batch reproducibility (Karnik et al., 2008; Li & Jiang, 2018; Rhee et al., 2011), and are expected to accelerate the translation of polymeric NPs to a clinical setting, as they did for lipid NPs. The reader is addressed to extensive reviews on the production of polyester NPs by microfluidic (Li & Jiang, 2018) and flash nanoprecipitation (Saad & Prud'Homme, 2016) for a detailed description.

Spherical geometry can be altered to form anisotropic structures (elongated NPs) by including spherical NPs in water‐soluble films that are then stretched (Champion et al., 2007), whereas needle‐shaped and cylindrical NPs can be produced by soft‐lithography methods as particle replication in nonwetting templates (PRINT; Bowerman et al., 2017; Chu et al., 2013; Rolland et al., 2005).

### 3.3 Surface functionalization

#### 3.3.1 Layering strategies

**FIGURE 3:**
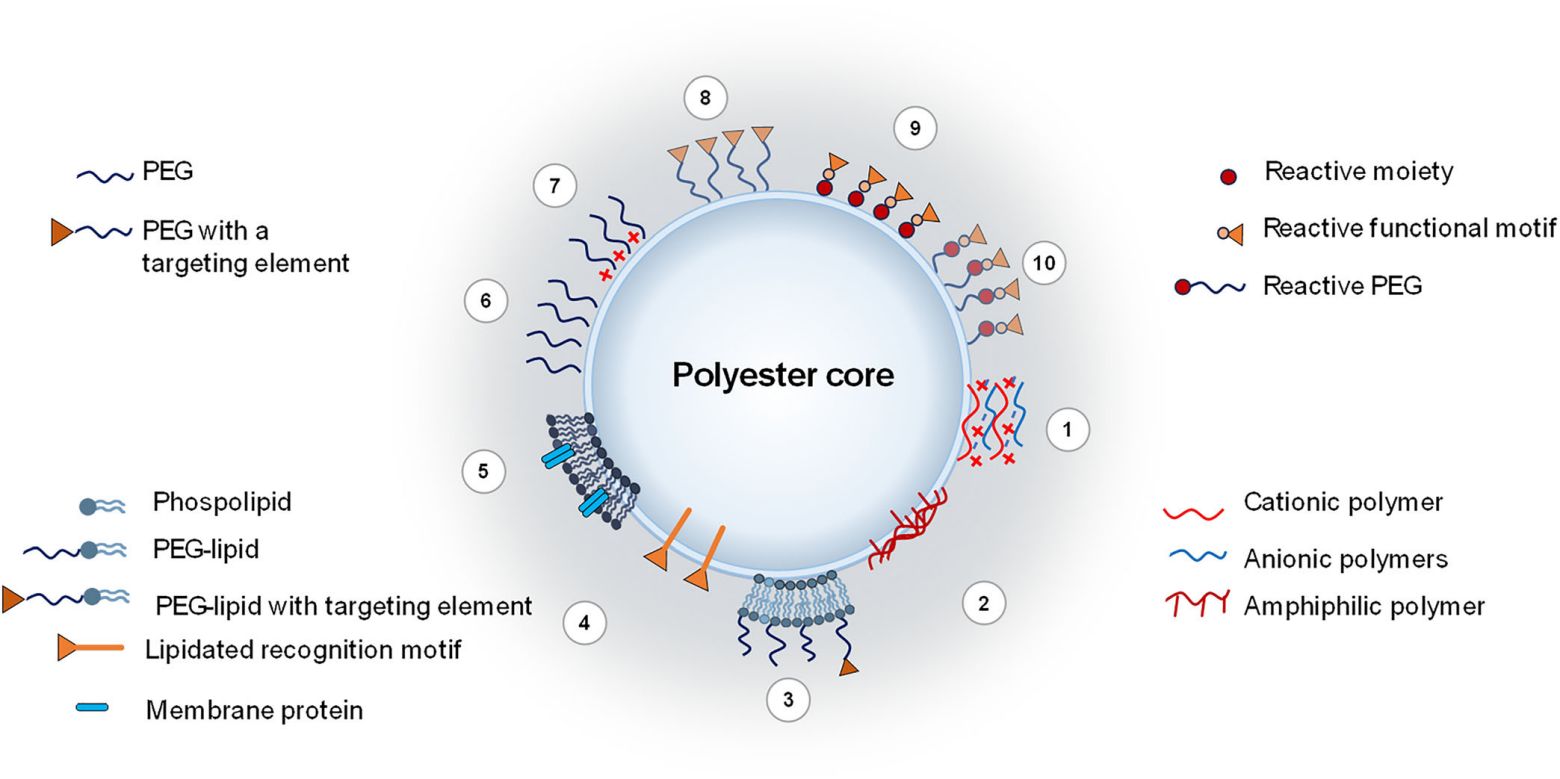
Overall view of the strategy currently employed to obtain core–shell NPs. Layering strategy: (1) multilayer coating; (2) integration of an amphiphilic graft‐polymer; (3) phospholipid coating (a bilayer is reported as representative example); (4) integration of a lipidated recognition motif; (5) camouflage with biological membranes) from cells or extracellular vesicles) on a polyester core. Assembly of PEGylated polyesters: (6) PEGylated NPs; (7) PEGylated ionizable (positive) NPs (in this case mixtures of PEGylated and amine‐functionalized polyesters are used); (8) Functionalized PEGylated NPs. Post‐modification of NPs (9) made of reactive polyesters or (10) made of reactive PEGylated polyesters with a reactive functional motif. Post‐modification via streptavidin–biotin binding is also feasible.

Surface modification of the pristine polyester NPs can be achieved by mixing the polyester with other components (polymers, surfactants, or lipids) having a comparable solubility profile or through noncovalent adsorption of the surface modifier on preformed polyester nanocore (Figure 3, strategies 1–5). For instance, PLGA/Poloxamer blends were proposed to modify the hydrophilicity of the polyester matrix to facilitate the entrapment of fragile macromolecules like growth factors and finely tune their release rate, preserving their structural integrity (Csaba et al., 2005; Parajó et al., 2010).

By exploiting the negative charge of NPs made of non‐end capped polyesters, the adsorption of cationic polymers such as chitosan oligomers or polyethyleneimine is encouraged, and a charge switch to positive values occurs. Although the cytotoxicity of the cationic polymer is alleviated, positive NPs are unstable in pharmaceutical vehicles undergoing aggregation (Maiolino, Russo, et al., 2015) and may elicit an immune response, fueling new surface engineering strategies.

Multilayer coating technology stems from the observation that polymers with opposite charges can be sequentially deposited on a polymer nanocore through charge interactions (Park et al., 2003). For pharmaceutical applications, PLGA nanocores surrounded by a certain number of layers of biocompatible polymers were achieved by alternatively soaking NPs in polymer solutions containing positively or negatively charged polymers (Morton et al., 2013; Romero et al., 2013). The type and number of layers affected the release feature and helped achieve NPs with antifouling properties. A main caveat with this strategy is the lengthy fabrication procedures that require multiple steps of alternating coatings and centrifugation, which makes scale‐up difficult. To simplify the method we have demonstrated that a double layer of the cationic polymer polyethyleneimine (bridging layer) and the anionic polymer hyaluronan (external layer) is stable enough to attain coated NPs entrapping a combination of chemotherapeutics in both the core and the shell with specificity toward CD44 overexpressing cancer cells (Maiolino, Moret, et al., 2015; Maiolino, Russo, et al., 2015; Russo et al., 2016).

The surface modification of polyester NPs with lipids is another appealing strategy to functionalize NPs, merging the unique attributes of liposomes and polymeric NPs in a very flexible design (Bose et al., 2016; Hadinoto et al., 2013; Mandal et al., 2013; Mukherjee et al., 2019). These hybrid lipid–polymer NPs conceptually developed 20 years ago (De Miguel et al., 2000) have gained a renewed interest in drug delivery since they offer the opportunity to control both the core and shell features precisely (Zhang et al., 2008) and deliver drug combinations (Fraix et al., 2020; Kemp et al., 2016; Rawal & Patel, 2019; Wang, Alshaker, et al., 2017). They consist of a polymer nanocore useful to entrap, protect, and release the drug payload, whereas the surrounding monolayer or bilayer of phospholipids imparts specific surface features to the nanocarrier. Among the lipids used for the surface modification of PLGA NPs, lecithin, and synthetic lipids with different chemistry (phosphatidylcholine‐PC, methoxyPEG‐1,2‐distearoyl‐sn‐glycero‐3‐phosphoethanolamine (DSPE‐PEG), cholesterol) are commonly employed. The use of 1,2‐dioleoyl‐3‐(trimethylammonium) propane chloride salt (DOTAP) or DC‐Chol was also proposed to achieve positive NPs (Bose et al., 2016; Yu et al., 2018). These hybrid lipid‐polyester NPs have demonstrated potential to deliver small interfering RNA (siRNA) to glioblastoma cell lines, setting the stage for using the nanoplatform to silence genes relevant to glioblastoma (Rinaldi et al., 2024).

One‐step fabrication is feasible when lipids and polyester are soluble in a common solvent such as methylene chloride and employing emulsion methods. Although the lipid components are added in the organic phase, they migrate on the NP surface due to their amphiphilic features and form a layer surrounding polyester NPs (Conte et al., 2022; d'Angelo et al., 2018). It is also feasible to achieve polyester nanoprecipitation in a water phase containing predispersed lipids. Lipids spontaneously form a lipid monolayer covering the polyester nanocore in this case. Hierarchically controlled assembly of PEGylated lipids on a PLGA core can generate PEGylated hybrid NPs with a reduced tendency to aggregation and burst release (Ghitman et al., 2020; Sivadasan et al., 2021). The lipid shell‐polymer core architecture can be beneficial in delivering drug combinations. We have devised a strategy to generate hybrid lipid‐polymer NPs carrying a chemotherapeutic in the PLGA core and a second therapeutic in the lipid shell to avoid any spectral communication between the therapeutic species (Fraix et al., 2020). To this purpose, a PLGA acetone solution containing the chemotherapeutic was nanoprecipitated in an aqueous dispersion of preformed lipid vesicle loaded with the second chemotherapeutic and then dried to give hybrid lipid–polymer NPs. In the two‐step method, a polymeric dispersion of NPs is used to hydrate the dried lipid film or mixed with the preformed lipid vesicles under vortexing, sonication, or extrusion at a temperature higher than the gel‐to‐liquid transition temperature of lipids.

PEGylated lipids bearing a targeting element can provide surface‐functionalized NPs by single‐step self‐assembly and nanoprecipitation, as reported for folate‐modified PLGA NPs (Wang et al., 2010; Zheng et al., 2012). Spontaneous migration of amphiphilic peptide toward the NP surface during nanoprecipitation has helped display recognizing elements on the NP surface (Conte et al., 2023; da Silva Filho et al., 2021).

Surface functionalization using cell membranes derived from cells is an emerging strategy at the interface between biology and pharmaceutical technology introduced to impart polyester NPs with many unique characteristics associated with natural cell membranes (Luk & Zhang, 2015; Pasto et al., 2019). A large variety of cell types, including red blood cells (RBCs), platelets, white blood cells, macrophages, cancer cells, and stem cells, have been employed as sources of cell membranes to coat synthetic NPs (Chugh et al., 2021; He et al., 2020). The coating procedure includes the multiple steps of (i) isolating and purifying cell membrane fragments, (ii) fabrication of the inner core, and (iii) coating the inner core with purified cell constituents. Some key points of the coating technology should be considered for effective translation. The isolation of cancer cell membranes is a complex process, and current membrane extraction techniques can hardly guarantee the integrity of the tumor cell membrane. Furthermore, cancer cell membrane coating is commonly achieved through sonication and extrusion processes after mixing cancer cell membrane vesicles and NPs. The coating efficiency is sometimes largely compromised, and heterogeneous populations of NPs without membrane coating and some membrane vesicles without a NP core are obtained (Liu et al., 2021). The advent of microfluidic technologies is expected to simplify the coating process and guarantee batch‐to‐batch reproducibility (Liu et al., 2019). The reader is addressed to recent reviews discussing the fabrication methods of cell membrane‐coated NPs, highlighting the possible issues encountered along the coating process (Fang et al., 2018; Imran et al., 2023; Liu et al., 2023).

#### 3.3.2 Assembly of amphiphilic PEGylated polyesters

The assembly of PEGylated polyesters gives rise to corresponding core‐shell NPs where a polyester core is surrounded by a PEG shell unmodified (methoxy end groups are exposed) or modified with a functional moiety (often a targeting element) (Figure 3, strategies 6–8). PEG‐polyester NPs obtained by nanoprecipitation and emulsion‐solvent evaporation give monodispersed spherical NPs without needing aqueous phase stabilizers. Depending on polymer properties and experimental conditions adopted, PEG‐PCL and PEG–PLA can also form perfect core‐shell micelles, filomicelles, or polymersomes (Shenshen Cai et al., 2007; Loverde et al., 2011; Zhang et al., 2014). Filomicelles are worm‐like core‐shell structures with a larger core volume per carrier and almost twice higher drug loading capacity than spherical micelles. Polymersomes are vesicles consisting of a hydrophobic polymer bilayer surrounding an aqueous core and prepared by self‐assembling PEG‐polyester block copolymers of specific amphiphilicity in an aqueous solution. These nanovesicles can be loaded with hydrophobic molecules in their bilayer and hydrophilic molecules in their aqueous core (Liu et al., 2012). Similar to liposomes, solvent evaporation, and thin‐film hydration are the most broadly used methods for preparing filomicelles and polymersomes. The main factors that influence the self‐assembly process and the geometry of the corresponding nanosystem include the chain length, the molecular weight fraction of the hydrophilic segment, the ratio of the hydrophilic and hydrophobic segments, and the nature and length of the hydrophobic segments (Discher & Ahmed, 2006; Geng et al., 2007; Loverde et al., 2011; Zhang et al., 2014). Hydrophilic volume fraction (*fEO*) lower than 25% in linear amphiphilic copolymers leads to aggregation and solid particle formation, while *f*_*EO*_ between 25 and 40% produces polymersomes (Blanazs et al., 2009). Micelles and hollow tubes are prepared at *f*_*EO*_ values higher than 50% and 40–50%, respectively. The structure of the assemblies can be predicted to some extent from the packing parameter (p) that relates to the sizes of both blocks.

In general, PLA–PEG diblock copolymers tend to form star‐like micelles and, in some cases, polymersomes, whereas PEG–PCL can also give filomicelles and polymersomes. Conversion of polymersomes into filomicelles and finally into spherical micelles has been observed upon PLA–PEG degradation (Ahmed et al., 2006). By proper synthetic strategies, one PEG end group can be modified with small ligands or peptides that, once correctly displayed on NPs, are expected to promote the selective accumulation of the drug cargo in the target. Functionalized PEG–polyesters can be used alone or mixed with bare PEGylated polyester to achieve different densities of surface ligand exposure. However, the entanglement of the ligand‐bearing PEG chains in the NP core, especially for lipophilic targeting ligands, may limit its surface location a designed density, making targeting elusive (Abstiens et al., 2019; Venuta et al., 2018). The addition of cyclodextrins able to complex the lipophilic targeting ligand during NP formation can encourage the exposure of molecules such as folate and improve uptake in cancer cells overexpressing the Folate receptor‐α (Conte et al., 2016).

#### 3.3.3 Post‐modification

Surface‐modification of PEGylated NPs with a functional element can be achieved via covalent post‐insertion on preformed particles (Figure 3, strategies 9–10). Post‐modification has been devised for large molecules like antibodies, targeting proteins, or aptamers since synthetic procedures to modify polyesters rarely guarantee the integrity of the targeting element and sufficient reaction yields. The recognition elements are anchored to the NP surface through mild chemical reactions. Post‐modification of PEGylated polyester NPs has been exploited with monoclonal antibodies (Acharya et al., 2009; Kocbek et al., 2007; Liu et al., 2010; Rosalia et al., 2015), transferrin (Yue et al., 2011), and aptamers (Cheng et al., 2007; Guo et al., 2011; Yu et al., 2011). Finally, post‐modification of PEGylated lipids coating PLGA NPs with aptamers was feasible too (Aravind et al., 2012).

Due to several issues, the post‐modification strategy, which is highly suited for inorganic NPs, hardly applies to polyester NPs. The orientation and density of the targeting molecule during the coupling reaction are poorly controlled, and the release of drug cargo, the degradation of the polymer matrix, and reagent penetration inside NPs can take place (Chatterjee & Chanda, 2022; Gessner & Neundorf, 2020; Nicolas et al., 2013). Furthermore, the purification from unreacted reagents and by‐products can be challenging for degradable materials, finally impairing the safety and integrity of the functionalized NPs.

### 3.4 Fabrication challenges

#### 3.4.1 Tuning critical attributes

Size is a critical feature of spherical NPs that governs PKs, tumor penetration, and cell uptake. NPs in the 50–200 nm range are usually preferred for intravenous (IV) administration to avoid obstruction of the tiny capillaries. Polymer composition, MW and concentration, type, and concentration of stabilizers, organic solvent type, and solvent/water phase ratios are the main critical formulation parameters to manipulate the size (Hernández‐Giottonini et al., 2020). An increase in MW and concentration of PLGA are associated with an increase in the mean diameter of resulting NPs, independently of the fabrication technique. This is because these features affect the viscosity of the organic phase, thereby decreasing the net shear stress and increasing the particle size. It is demonstrated that solvent diffusion into the external phase slows down with increasing viscosity of the organic phase and provokes the formation of large polymer aggregates (Beck‐Broichsitter et al., 2010). During nanoprecipitation, a faster nucleation rate results in smaller‐sized NPs (Huang & Zhang, 2018; Lepeltier et al., 2014). The other critical factor is the presence and concentration of the emulsifier, which plays a key role in separating the polymer‐containing phase from the dispersion medium (Hernández‐Giottonini et al., 2020). While extensive literature is available on the effect of the operating conditions on the final size distribution of PLGA NPs, very few works have focused on PCL NPs (Badri et al., 2017).

Actual drug loading and encapsulation efficiency of NPs strictly depend on the drug solubility and fabrication technique. Each drug requires an ideal combination of polyester type, solvent, and fabrication conditions, drug entrapment, and release kinetics being the main properties affected. Most chemotherapeutics are lipophilic, soluble in the polyester solvent, and easily loaded in NPs via single emulsion or nanoprecipitation methods. To achieve high loading of hydrophilic drugs, specific formulation strategies are implemented. In fact, the tendency of these drugs to leak into the external water phase is the main issue. In this case, the emulsification method typically provides increased drug loading compared to the nanoprecipitation method. Playing with drug ionization, ion‐pair excipients, salts, and water‐miscible solvents (ethanol) added in the aqueous phase, as well as pH adjustment, can limit the leakage of the drug in the external aqueous phase and increase the encapsulation efficiency. The use of uncapped polyesters for positively charged API represents another viable solution. In general, polyester NPs exhibit drug loading <20%–30%, which might prove problematic for less potent drugs.

The loading capacity of the NPs is affected by size. Larger‐sized PLGA NPs (>300 nm) are generally associated with increased drug loading capacity, whereas PLGA NPs with a relatively small size range (50–100 nm) display lower loadings, hardly higher than 20%. Several studies on PEG‐PCL‐based filomicelles for anticancer drug encapsulation showed that higher drug loading contents and encapsulation efficiency could be achieved compared to spherical counterparts (Cai et al., 2007; Ridolfo et al., 2020).

NP degradation rate and drug release kinetics are affected by NP size, with a tendency for smaller PLGA particles to degrade faster, resulting in a relatively rapid drug release compared to larger PLGA particles (Bennett et al., 2016). Polymer crystallinity can impact the loading efficiency and release rate of the drug cargo. For IV NPs, burst release is especially detrimental during blood circulation since a significant amount of drug is released before reaching the target site. Potential ways of mitigating burst release include alterations to drug−polymer interactions, polymer chemistry, spatial arrangement of drugs in the polymer matrix, and coating with a polymer or a lipid layer (Aryal et al., 2013; Bose et al., 2016; Lu et al., 2019).

In a recent study, prediction graphs of drug‐loading capacity and entrapment efficiency of PLGA NPs by the Gaussian Process, a machine learning (ML) algorithm, was proposed to eliminate the trial‐and‐error discovery method and save laboratory time (Noorain et al., 2023).

#### 3.4.2 Control of surface features

Surface features dictate the chemical identity of NPs and are recognized to modify nano‐biointerface. The presence of residual stabilizers in the final formulation is generally overlooked and difficult to quantify, jeopardizing the interpretation of biological performance.

In the case of PEGylated NPs, the precise control of PEG density on the surface is a key aspect of the fabrication process since it is the main determinant of NP in vivo behavior. The use of PEG‐polyesters, indeed, does not ensure that PEG chains are quantitatively displayed on the NP surface since PEG chains can accommodate in the lipophilic core due to the peculiar solubility profile of PEG, which is soluble in both aqueous and organic solvents (Xu et al., 2015). The rate of phase separation of PEG‐PLGA NPs, faster with nanoprecipitation than emulsion techniques, was demonstrated to affect PEG surface exposure (Xu et al., 2015). Thus, full control of the amount of PEG on the number of PEG chains covering the NP surface is challenging to attain. As a result, the actual PEGylation extent of NPs is much lower than expected. Since the threshold for maximum achievable PEG surface density may vary depending on the type of NPs and formulation methods, this aspect needs to be considered case by case.

It is worth noting that the quantitation of PEG on the NP surface is hardly carried out, making the impact of the extent of PEGylation of NPs on biological behavior poorly rationalized. The most relevant techniques used to assess qualitatively or quantitatively PEG chain coverage‐density, conformation, and layer thickness on polymeric NPs are fully described in Rabanel et al. (2014). ^1^H‐NMR is considered the most robust and straightforward technique for evaluating the PEG amount on NPs and deriving the PEG surface density.

The surface topology critically affects the target ability for NPs functionalized with a recognition element. The amount of ligand displayed by NPs of PEGylated diblock polyesters is strictly related to the physical–chemical properties of the surface‐functionalizing agent. Poorly water‐soluble small ligands can remain segregated in the NP core due to their preferential solubility in the organic solvent, as we found in the case of folate‐functionalized PEG‐PCL NPs (Venuta et al., 2018). This drawback is expected to be much less relevant for hydrophilic recognition elements. We purposely used HPβCD during nanoprecipitation of mPEG‐PCL and folate‐PEG‐PCL to increase the surface density of PEG and the targeting ligand, taking advantage of the complexation between HPβCD with PEG chains or folate‐PEG (Venuta et al., 2018). This study inspired us to develop a rotaxanated folated‐PEG(aCD)‐*b*‐PCL copolymer to produce targeted NPs, demonstrating increased selectivity for KB cells (Dal Poggetto et al., 2020).

The stabilizers employed in NP formulation can remain anchored to the NP surface. Polyvinyl alcohol (PVA) is the most commonly used stabilizer for making polyester NPs using emulsion techniques. The interpenetration of PVA into the hydrophobic emulsion droplets, while contributing to their stabilization, results in its entrapment into the core matrix of the NPs (Carrio et al., 1995). Thus, washing out residual PVA from the NP surface is very difficult. The amount of PVA associated with NPs depends on its chemistry and amount employed, the polyester chemistry, the formulation procedure, and the NP size. As much as 13 wt% of PVA residue was reported for PLA NPs (Zambaux et al., 1998). Considering numerous reports studying polyester NPs prepared by the emulsification method using PVA of various MW and degrees of hydrolysis, it is likely that the biological outcomes could have been influenced by the surface residual PVA (Suk et al., 2016). Small molecule emulsifiers, such as cholic acid, sucrose esters, and dioctyl sulfosuccinate sodium, were found to produce stable emulsions without affecting the PEG surface coating and proposed as an alternative to preparing PEG‐PLGA NPs (Xu et al., 2015). d‐α‐Tocopheryl polyethylene glycol 1000 succinate (vitamin E TPGS or TPGS) has also been proposed as a stabilizer for PEGylated NPs (Sun et al., 2014).

#### 3.4.3 Processing issues

Purifying polyester NPs to eliminate surfactant and unloaded drugs is essential and usually carried out using centrifugation and dialysis at the laboratory scale. To perform the entire process in continuous mode, all the unit operations need to be integrated with the capability of recycling streams and purging impurities as required. The foremost exploited continuous downstream purification processes are based on membrane separation techniques (tangential‐ or cross‐flow filtration) and continuous flow centrifugation, a standard in the pharmaceutical industry. It is crucial to face this aspect in the early development phase to anticipate possible issues envisaging a formulation scale‐up.

Finding an appropriate sterilization method is crucial in manufacturing since most polyester NPs delivering chemotherapeutics are parenteral products. Each preparation must be validated on a case‐by‐case basis as NPs can be affected differently by the sterilization method, depending on their components, formulation, and/or preparation method (Ragelle et al., 2017). Aseptic manufacturing remains the best option in continuous production processes, where production is isolated from the surrounding environment and operators. From a technological standpoint, sterilizing the final formulation remains an open issue for parenteral products based on nanotechnologies. Terminal sterilization is always preferred over aseptic processing due to production costs lower than aseptic production. Gamma‐irradiation is the terminal sterilization method for PLGA formulations (Jiang et al., 2005) successfully employed for biodegradable PLGA microspheres intended for parenteral use (Desai et al., 2013). The ionizing radiation on biodegradable polyesters, however, causes a radiation dose‐dependent decrease in polymer molecular mass by radiolytic chain scission and formation of reactive radicals that accelerate polymer degradation rate (Hausberger et al. 1995) and change release kinetics (Mohr et al., 1999). Furthermore, drug stability under irradiation can be affected (Bozdag et al., 2005; Mohr et al., 1999; Montanari et al., 2001). It is relevant that some protective sugars added as diluents/cryoprotectants for freeze‐drying the product as glucose or mannitol can alleviate this issue (Galante et al., 2016). An alternative for polyester NPs can be sterile filtration, which is feasible in principle for NPs of a specific size and low polydispersity, simplifying the overall production flow under GMP conditions (Masson et al., 1997).

#### 3.4.4 Long‐term stability

For pharmaceutical applications and due to polyester degradation in an aqueous environment, NPs dispersions must be dried to ensure long‐term stability and shelf‐life. This operation is challenging for colloidal formulations in general and scarcely addressed in formulation development (Abdelwahed et al., 2006; Fonte et al., 2016). Freeze‐drying and spray‐drying are the most popular techniques for producing a dry product. The challenge is to retain NP physicochemical properties and avoid aggregation phenomena that are not reversible. After reconstitution, NPs should possess the same size distribution profile, PDI, loading, and release rate, which are key factors that impact their biological performance. Furthermore, low residual moisture content and good long‐term stability are required to avoid a decrease of polymer *T*_g_ below room temperature (Passerini & Craig, 2001).

Optimizing the formulation, process, and storage conditions are crucial in the case of freeze‐drying. During freezing, a separation of phases into ice and a cryo‐concentrated suspension occurs. The cryo‐concentrated phase is constituted by NPs and other excipients like cryo‐ and lyo‐protectants, buffers, surfactants, and even unloaded drugs (Abdelwahed et al., 2006). Although considered a gentle drying process, the high concentration of NPs in the cryo‐concentrated phase and the pH change when buffers are used may cause their aggregation or fusion. Furthermore, the ice crystallization may induce mechanical stress on NPs, destabilizing them. Desorption of loosely entrapped drugs has been reported as well (De Chasteigner et al., 1996).

Short‐chain saccharides (mannitol, trehalose, sucrose, and glucose) routinely employed for the freeze‐drying of biopharmaceuticals are also used to minimize NP instability during both the freezing steps (cryoprotectants) and drying step (lyoprotectants) (Abdelwahed et al., 2006; Trenkenschuh & Friess, 2021). The water replacement theory and the vitrification concepts, evoked to explain the stabilization of proteins upon freeze‐drying, can, in principle, explain how sugars stabilize colloidal systems. Hydrogen bonding is considered most effective when sugar molecules tightly fit the irregular colloid surface, which requires their amorphous state (Grasmeijer et al., 2013). In the water replacement theory, sugar molecules are supposed to replace the hydrogen bonds of water at the surface of a colloid, maintaining the molecular structure upon drying (Carpenter & Crowe, 1989). The vitrification theory (or particle isolation hypothesis) assumes that colloids are immobilized in a rigid, amorphous, glassy sugar matrix, drastically slowing their diffusion, aggregation, and fusion (Mensink et al., 2017). Consequently, below the glass transition temperature (*T*_g_), the amorphous matrix is in a rubbery state, and kinetic immobilization is lost. In analogy to protein therapeutics, both mechanisms are expected to play a role (Grasmeijer et al., 2013).

The selection of an adequate cryo‐ or lyo‐protectant and freezing rate for colloids is not straightforward, and it may depend on both the formulation properties and the lyophilization cycle (Fonte et al., 2016).

The impact of formulation and use of additives on the properties of polyester NPs has been recently reviewed (Trenkenschuh & Friess, 2021). The different constituents of formulations, such as the type of polymer and surfactant, the chemistry of NP surface, and the nature and concentration of cryo‐ or lyo‐protectant used, may influence those properties and then have important effects in the different stresses caused by lyophilization (Fonte et al., 2016). PVA remaining on polyester NPs also after repeated washing steps is reported to stabilize NPs during freeze‐drying (Berton et al., 1999; Fonte et al., 2016; Murakami et al., 1999), whereas Pluronic® F68 has the opposite effect (Quintanar‐Guerrero et al., 1998).

Optimization of the freeze‐drying process often implies an empirical approach with trials and errors. Therefore, it is crucial to perform systematic studies by assessing the physical–chemical properties of formulations and understanding the engineering principles inherent to lyophilization. DoE can be very useful in optimizing the different phases of the freeze‐drying process, as reported in the case of solid lipid NPs (Elbrink et al., 2023). Nevertheless, Artificial Intelligence (AI) tools, such as Neurofuzzy Logic systems, have been proposed to establish a suitable lyophilization procedure using carbohydrates as cryoprotectants rationally. This approach is expected to gain a better understanding of the impact of lyophilization conditions (cryoprotectant type and concentration or freezing speed) on lyophilized nanocarrier properties and also to assess the physicochemical phenomena driving sugar effectivity (Rouco et al., 2021).

Continuous freeze‐drying technologies are now being developed for bulk products and unit doses. Spin‐freeze‐drying and continuous spray freeze‐drying in aseptic conditions are innovative drying technique that promises to revolutionize the conventional batch‐wise process of single‐unit freeze‐drying (Adali et al., 2020; Van Bockstal et al., 2017).

Spray‐drying is another process widely applied in the pharmaceutical field to obtain discrete microparticles that can be employed as powders or reconstituted in an appropriate vehicle before administration (Broadhead et al., 1992). This one‐step process consists of nebulizing a feeding solution that is dried by a hot gas stream (air or nitrogen, depending on drug stability). Adding bulking excipients in the feeding liquids, selected based on their biocompatibility with the intended administration route, allows production powders with satisfying flow and redispersion properties and increases process yield. The optimization of SD conditions (feeding liquid composition and rate, inlet and outlet temperature, bulking agents type, and concentration) is critical to preserve drug integrity and ensure satisfactory yields. Readers are addressed to a recently published book chapter discussing the spray‐drying process for pharmaceutical powder production (Vehring et al., 2020).

Spray‐drying can be extended to feeding liquids containing preformed drug‐loaded NPs, thus becoming a general drying method. The final formulations are powders of the bulking agent(s) where NPs are dispersed (nano‐in‐micro or nano‐embedded microparticles). Drying NP‐containing feeding liquids is not trivial at all. It is generally agreed that NPs accumulate at the droplet surface during drying (Vehring et al., 2020). The distance between single NPs decreases as the droplet shrinks, and aggregation of the particles may occur depending on the nature of their mutual interactions. The morphology of such particles can be altered by adding a surfactant to modify these repulsive or attractive forces (Vehring et al., 2020).

Spray‐drying has found an extensive application in manufacturing powders embedding polyester NPs intended for inhalation withstanding specific aerodynamic features to reach the target tissue (Jensen et al., 2010; Lababidi et al., 2020). Mannitol, lactose, and leucine are the bulking agents most often employed to produce SD powders. Maltodextrins of a specific grade (DE38) helped ensure prompt redispersion of PLGA NPs (Magri et al., 2019), although their use is limited to oral products. The evolution of sterile spray‐drying processing is underway and, in principle, valid for producing parenteral products based on nanotechnologies.

### 3.5 Design of experiment in the development of polyester NPs

The design of the experiment (DoE) is a suitable approach to optimize the nanoformulation and define the formulation space. DoE is becoming widely used as a tool for developing and optimizing experimental procedures with fewer observations while still providing the desired information on the correlation between the experimental and the response variables. The obtained model can then predict future observations within the original design range (Mäkelä, 2017). Another benefit of DoE is a deep knowledge of the correlation between the experimental factors and the properties of produced NPs, which can elucidate how NP physicochemical properties (critical attributes) affect biological performance. Some of the latest applications of DoE in the formulation of nanocarriers used for drug delivery across many different applications have been recently revised (Rampado & Peer, 2023).

DoE workflow is multistep consisting of (i) defining clearly the process, including a careful selection of the Critical Process Parameters (CPPs) to be investigated and the CQAs that will be measured; (ii) the selection of a specific design to define the amount of information that can be acquired; (iii) the running of the planned experiments minimizing the experimental variability; (iv) the data analysis through software to select the optimal factors settings according to the goals, and allow for the addition of new runs to improve the design. After modeling, it is also possible to run a set of control experiments at a given CPP level within the experimental space to check the robustness of the model.

DoE needs the selection of Critical Quality Attributes (CQAs) that are, by definition, physical, chemical, biological, or microbiological properties or characteristics that should be within an appropriate limit, range, or distribution to ensure the desired product quality. CQAs are critical limits that will bring the needed results to the product when kept under appropriate control. In parallel, DoE takes into account Critical Process Parameters (CPPs), namely those factors or settings that are considered critical for the final quality of NPs that can be quantitative (e.g., temperature, concentration of components in the formula, stirring speed, flow rates in microfluidics systems), qualitative (e.g., using different lipids or polymers with different features in the formulations) or both (e.g., a molar ratio of the different NPs components).

The robustness of DoE is closely related to the quality of CPP and CQA data, which is arguably the most challenging step in implementing Quality by Design (QbD). CQAs should be accurately and precisely measured using robust instruments and techniques that need to be previously validated. One of the prevailing challenges in nanomedicine development that is recognized to limit their clinical potential is indeed the uncertainty on well‐established quality checkpoints from the early development stage (Taha et al., 2020). The lack of guidelines for evaluating CQAs and corresponding technical specifications of nanomedicines represents a bottleneck when aiming at clinical translation. The indications of MIRIBEL, a list of standards and protocols to provide experimental reporting to improve reusability, quantification, practicality, and quality of published data (Faria et al., 2018), can represent a guide to measure CQAs for nanomedicines. In this respect, particle size, colloidal stability, drug loading capacity and stability, and drug release behavior are critical since they dramatically affect their physicochemical and biological performance. In some software, it is possible to create a hierarchy of CQAs, defining the most important ones for the objective of the study.

An increasing number of formulation studies are based on DoE, also in the case of polyester NPs (Diwan et al., 2020; Diwan et al., 2021; Esteruelas et al., 2022; Gonzalez‐Pizarro et al., 2018; Jena et al., 2022; Ramalho et al., 2019; Vardhan et al., 2017). In general, the DoE procedure is employed for formulation optimization and is hardly intended to link CQAs to biological performance, which is an aspect that deserves more attention in the future.

## 4 TUMOR HOMING OF POLYESTER NANOPARTICLES

**FIGURE 4:**
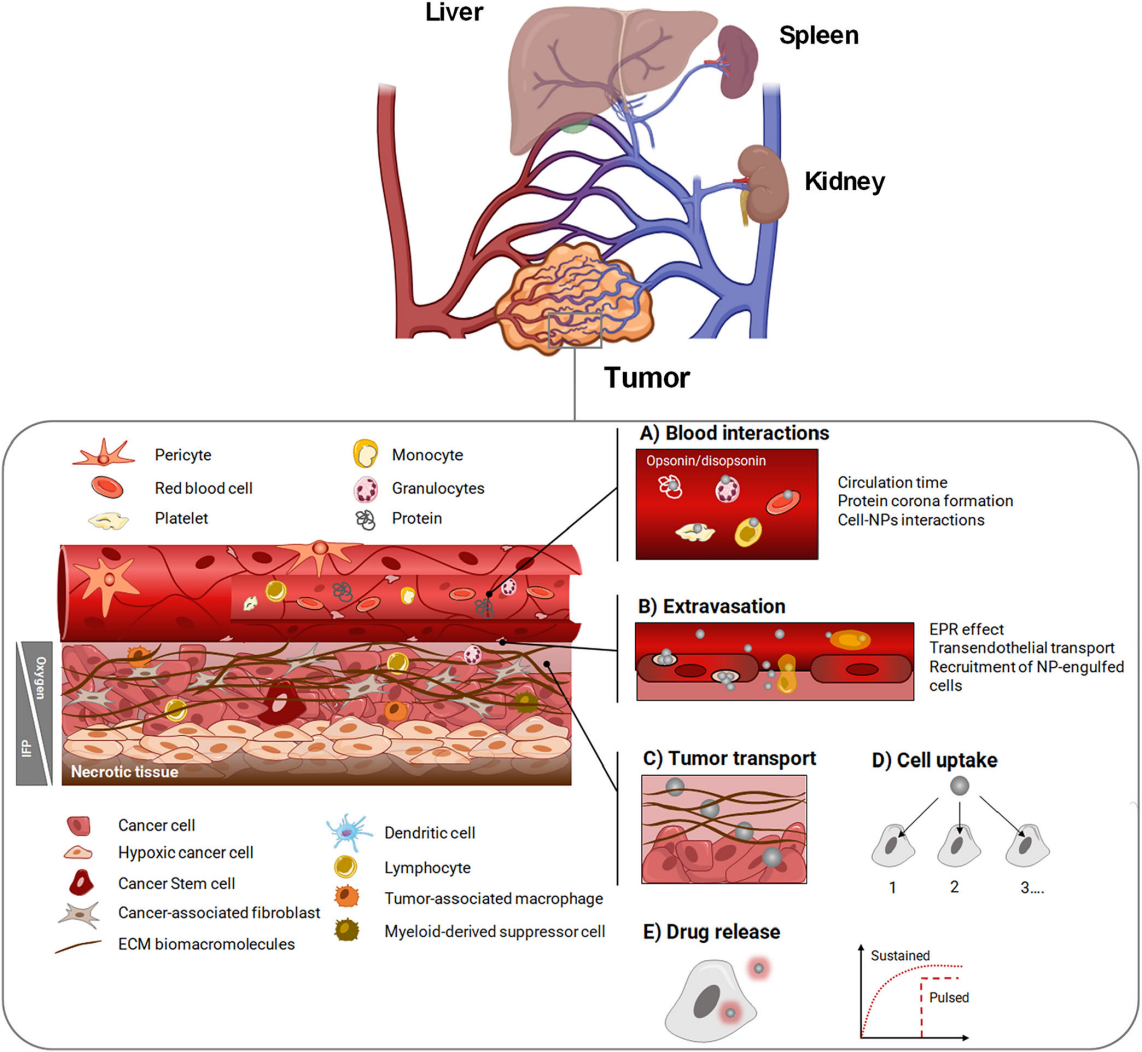
Schematic illustration showing the fate of NPs after i.v. administration. Liver, spleen, and kidney are the main organs where NPs accumulate. The interaction of NPs with noncellular and cellular components in the blood (a), that is protein corona formation and interaction with/engulfment in circulating cells, are determinant for organ distribution. Upon extravasation through different mechanisms (b), tumor transport rate of nanoparticles through dense ECM (c) and uptake of nanoparticles in diverse cell subtypes (d) can alter local drug biodistribution and redirect the drug payload toward specific tumor‐resident cells. Drug release from NPs can be extracellular or intracellular and programmed at specific rate (e). The figure is not exhaustive, and excludes cell subsets, such as eosinophils, innate lymphoid cells, and mast cells, that may also play a role.

The pathophysiological characteristics of solid tumors significantly influence the effectiveness of chemotherapy. Abnormal angiogenesis and lymphangiogenesis, irregular sluggish blood flow, higher interstitial fluid pressure compared to normal tissues, hypoxia, and the presence of an immunosuppressive environment are the most relevant pathophysiological aspects of solid tumors that collectively cause the physicochemical composition of the tumor microenvironment (TME) to differ from that of the typical interstitium in normal tissue. IV administration of chemotherapies is the most widely used and effective route to achieve fast and consistent dose distribution and reaches remote body compartments. However, attacking cancer from the systemic circulation is not easy since transport to and throughout the tumor is complex, even for a free small‐molecule chemotherapeutic, making the available local dose limited and difficult to foresee. Unlike small drugs or antibodies, IV nanotherapeutics have a poorly predictable journey in the body (Figure 4). The chemotherapeutic dose reaching the pharmacological target closely depends on the PKs and tissue biodistribution of the nanotherapeutics as driven by nanocarrier interaction with cellular and noncellular elements in the blood, premature drug release in the circulation and interaction with the TME (Anchordoquy et al., 2017; Blanco et al., 2015; Chauhan, Stylianopoulos, et al., 2011; Danhier et al., 2010). The interplay of all these aspects affects the therapeutic response and must be taken into account when developing a novel generation of NPs withstanding advanced design criteria, as discussed in the following sections.

### 4.1 The hallmarks of solid tumors

#### 4.1.1 Dysfunctional vasculature

Angiogenesis is a hallmark of cancers where new blood vessels are formed to supply oxygen and nutrients to the growing tumor. Tumor blood vessels, aberrant in structure and function and heterogeneous in size, create a chaotic and tortuous network with increased permeability that, in theory, should enable nanocarrier extravasation (De Bock et al., 2011; Nagy et al., 2009). These features facilitate protein transport into the interstitial tumor space, increasing local pressure, local RBC concentration, and blood viscosity. Lymphatic vasculature near tumors is denser than normal tissue, and lymphatic vessels can develop intratumorally (Alitalo & Detmar, 2012). Damage to lymphatic vessels and blocking of lymphatic drainage increases interstitial fluid accumulation (Stylianopoulos et al., 2018). These vascular abnormalities, combined with pressure from the TME with a high quantity of fibers and cells, induce a mechanical force that can compress or even collapse intratumoral vessels and severely reduce the blood flow into and from tumors (Heldin et al., 2004; Jain, 1987; Padera et al., 2004). High intrafluid pressure (IFP) makes tumor cells pump out of tumor tissues, facilitating metastasis seeding (Munir, 2022; Sindhwani et al., 2020). Lacking the arteriole–capillary–venule hierarchical organization typical of normal tissue, tumor perfusion is poor. In advanced human tumors, a suppressed blood flow often results in the formation of fibrin clots or thrombi (Islam et al., 2022; Young et al., 2012). Combining increased IFP with poor perfusion decreases oxygen and nutrient supply, the acidic and hypoxic microenvironment typical of solid tumors is generated, and cancer cells are pushed to a more aggressive phenotype (epithelial to mesenchymal transition) and to colonize other tissues (Jain, 2014). Emerging evidence also highlights the central role of tumor‐associated blood and lymphatic vasculature in thwarting immunosurveillance mechanisms and antitumor immunity (Schaaf et al., 2018).

The lymphatic system plays a crucial role in human cancer since 80% of solid tumors metastasize via the lymphatic system. Cancer stimulates lymphangiogenesis, and throughout cancer progression, lymphatic vessels located proximally to the tumor undergo extensive remodeling that favors and enables cancer metastasis through this route (Karaman & Detmar, 2014).

In the classical view of metastasis, cancer cells reach the sentinel node, the lymph node immediately downstream of the tumor that receives lymph proximally from the tumor area, spread to other regional lymph nodes and more distant nodes, reach systemic circulation, and form organ metastasis (Alitalo & Detmar, 2012). Lymphatic involvement is an essential indicator of disease progression and outcome, and there is a poorer prognosis for patients with cancer after their cancer has metastasized to the lymphatic system. Removal of metastasized lymph nodes is recommended to attempt to stop further cancer spread throughout the lymphatics or into other tissues and organs (Trevaskis et al., 2015).

#### 4.1.2 The complexity of tumor microenvironment

The complex nature of TME hosting cell populations of different origins embedded in a hydrogel‐like matrix is nowadays considered the main barrier to drug and NP transport to the target cell. The extracellular matrix (ECM) in solid tumors is so abundant that it represents up to 60% of the tumor mass. It is composed of biomacromolecules typically classified into three categories according to their functions: (i) structural proteins (collagen and elastin) organized into a fibrillar network that provides tensile strength; (ii) connexins (including fibronectin, laminin, and tenascin) that provide adhesive binding sites thereby facilitating the process of cell adhesion, spreading, migration and even differentiation; (iii) Proteoglycans and their grafted glycosaminoglycans, including HA, chondroitin sulfate, heparin, heparin sulfate, and keratan sulfate, forming a hydrogel‐like network with unique biophysical properties such as high compressive strength, viscoelastic effects, and streaming potential (Lu et al., 2012). The primary source of ECM components are cancer‐associated fibroblasts (CAFs), the major component of the stromal cells in tumor sites, that remain perpetually activated and promote the accumulation of collagenous ECM (desmoplasia; Conti et al., 2008).

Poor penetration and uneven distribution of chemotherapeutics in solid tumors are some of the most critical detriments to clinical efficacy. The presence of a dense and stiff ECM, the high IFP, and the poor tumor perfusion hamper the transport of free drugs to the deep tumor regions. Drug penetration is inversely related to the distance from vessels, and slow diffusion becomes the sole transport mechanism. Even free chemotherapeutics cannot always reach the tumor core and remain primarily at the edge of tumors, leading to partial effectiveness, resistant phenotypes, and metastasis (Primeau et al., 2005; Stylianopoulos & Jain, 2013). The “binding‐site barrier hypothesis,” formulated for antibodies (Juweid et al., 1992) and extended to small drugs, evokes that tumor transport is delayed due to interaction with tissue elements. Lack of cell exposure to the drug in tumor areas with poor drug penetration is likely to lead to pseudo‐resistance (Trédan et al., 2007).

Tumor microenvironments often harbor diverse cell populations, including, besides cancer cells, cancer stem cells, immune cells, fibroblasts, endothelial cells, and others. Their interplay and interaction with ECM are the basis of chemotherapy resistance mechanisms.

Cancer stem cells (CSCs) are a small subset of cells populating tumors (0.01%–2% of the total tumor mass) that share some properties with normal stem cells, including the capacity for self‐renewal and differentiation into multiple cell types found in the tumor mass (Ayob & Ramasamy, 2018). CSCs have the ability to initiate and sustain tumor growth, resist conventional cancer treatments like chemotherapy and radiation, and potentially cause cancer relapse or metastasis. CSCs have specific markers on their surface, along with general antigens and receptors (Walcher et al., 2020), which are organ‐specific, for example, CD44^+^/CD24 in breast cancer, CD133 in brain tumors, and epithelial‐specific antigens in pancreatic cancer. In addition to these stemness‐associated markers, epithelial cell adhesion molecule (EpCAM) is expressed on most CSCs.

TME is also populated by myeloid and lymphoid cells, including mononuclear cells and polymorphonuclear cells that migrate from the bloodstream and infiltrate tumors, regulating their growth. The term “tumor‐associated macrophages” (TAMs) refers to diverse cell populations derived from circulating monocytes but also of embryonic origin located in different intratumoral regions. M1‐like macrophages typically have pro‐inflammatory and anti‐tumor properties, whereas M2‐like macrophages often exhibit immunosuppressive and pro‐tumorigenic characteristics (Movahedi et al., 2010). The balance and polarization of TAMs between these different phenotypes can influence the TME dynamics and impact the tumor behavior and response to therapy (Pan et al., 2020). Dendritic cells populating tumors play a crucial role in initiating anti‐tumor immune responses by capturing tumor‐associated antigens and presenting them to T cells, thus activating and priming the immune system to recognize and target cancer cells (Gerhard et al., 2020; Wculek et al., 2020). This function is impaired in those tumors, evading immune recognition and suppressing immune responses (immune tolerance). Tumor‐associated neutrophils (TANs) are another relevant cell population with a complex role that varies depending on the specific context of the tumor, its stage, and the interactions with other immune and tumor cells (Wu & Zhang, 2020). Like macrophages, neutrophils can display different phenotypes and functional properties, exerting anti‐tumor activities or contributing to tumor progression (Masucci et al., 2019). Neutrophils inside tumors are generally associated with poor prognosis (Shen et al., 2014).

Tumor‐infiltrating lymphocytes (TILs) consist mainly of T cells (including CD4+ helper T cells and CD8+ cytotoxic T cells) and, to a lesser extent, B cells and natural killer (NK) cells. The presence of a higher number of infiltrating lymphocytes, particularly cytotoxic CD8+ T cells, within the TME, is associated with better prognosis and improved responses to some cancer treatments like immunotherapy (Badalamenti et al., 2019; Brummel et al., 2023).

Myeloid‐derived suppressor cells (MDSCs) are a heterogeneous population of immature myeloid cells that exert immunosuppressive functions, inhibiting T‐cell activation and proliferation, suppressing NK function, and promoting the expansion of Treg (Veglia et al., 2021). Their presence in tumors can dampen anti‐tumor immune responses and contribute to immune evasion by the tumor (Pittet et al., 2022).

#### 4.1.3 Tumor heterogeneity

The relative abundance of resident cells, the microenvironmental differences between primary and metastatic sites, the spatial variations in the microenvironment of an individual tumor, the cell plasticity, the cell–cell interactions, the probabilistic cell fate decisions, and the defects in gene expression are considered critical primary sources of tumor heterogeneity (Harris et al., 2019). Heterogeneity is partly responsible for why some patients who initially respond well to chemotherapy eventually relapse, frequently with new tumors that no longer respond to the therapy.

Characteristics of the TME include hypoxia, acidity, increased lactate and reduced glucose concentrations, secretome changes, and recruitment of stromal and immune cells (Boedtkjer & Pedersen, 2020). It is generally recognized that hypoxic areas overlap with acidic regions (Chiche et al., 2010). However, heterogeneous distribution of acidic areas has been observed outside hypoxic regions such as the tumor–stroma interface. Due to the dynamics of TME during tumor growth, the acidic status of the tissue considerably changes over time and in location (Gillies et al., 2018). The solid tumor stroma hypoxia contributes to increased collagen deposition and increased stiffness of ECM. It has been well‐established that cancer cells are under a higher degree of basal level oxidative stress than normal cells, reflected by an increased presence of reactive oxygen species (ROS) generated as a byproduct of cellular respiration and aerobic metabolism. Oxidative stress is a double‐edged sword to cancer cells and can exert either pro‐tumor or anti‐tumor effects. The delivery of antioxidants has been considered beneficial in attenuating oxidative stress and reducing the tumor‐promoting effects of ROS. Although successful in preclinical studies, this chemopreventive strategy failed to demonstrate real benefits in patients (Group, 1994) and is sometimes paradoxically linked to tumorigenesis in specific cancer types (Klein et al., 2011). In an opposite approach, the increase of ROS level in the tumor through pro‐oxidants or antioxidant inhibitors has been attempted to promote cancer cell death (Aboelella et al., 2021). The increase of ROS level in the tumor is at the basis of photodynamic therapy, a clinically approved localized treatment where a photosensitizing agent is activated by light of suitable wavelength to produce mainly gaseous singlet oxygen with cytotoxic, immunostimulatory, and antiangiogenic properties (Gunaydin et al., 2021; Li et al., 2020).

### 4.2 The challenges in tumor homing of intravenous NPs

#### 4.2.1 Circulation time and blood interactions

NP circulation time is considered critical for achieving extensive accumulation in solid tumors. It is closely related to the escape from circulating and organ‐residing cells of the mononuclear phagocyte system (MPS) and clearance in the kidney (Zhu et al., 2022).

The circulation time of nanocarriers is impacted by their interaction with plasma proteins and generation of a protein corona (nano‐bio interface), changing the chemical identity of the nanocarriers and imparting them a new biological identity (Lundqvist et al., 2008; Monopoli et al., 2012). The process is highly variable, dynamic, and strictly dependent on the physicochemical properties of the nanocarrier and the host circulating proteins.

Only advances in proteomics have allowed us to decipher this process and detect the complex mixture of protein adsorbed onto nanocarrier surface (Baimanov et al., 2019; Cedervall et al., 2007; Schöttler et al., 2016). The inner protein layer (hard corona), which is irreversibly bound to the nanocarrier surface, plays a thoughtful role in governing the downstream biodistribution of IV nanomedicines (Baimanov et al., 2019). The composition of the hard corona is initially dominated by the most abundant plasma proteins (albumin and fibrinogen; Vu et al., 2019). In the long run, proteins with low blood levels but high nanocarrier affinity and slow adsorption kinetics (apolipoproteins) instead play a major role (Cedervall et al., 2007). The type, amount, and conformation of the proteins adsorbed on the NP surface are key factors regulating the fate of the nanomaterial both in vitro and, ultimately, in vivo. Albumin, apolipoprotein, immunoglobulins, transferrin, fibrinogen, complement C3, haptoglobin, and α‐2‐macroglobulin are some of the most abundant proteins in the protein corona (Li, Wang, et al., 2021).

Surface chemistry and topography, charge, size, and shape are the main determinants of the multifaceted process of protein corona formation (Mahmoudi et al., 2023). For instance, lipid NPs target hepatocytes (Akinc et al., 2010) since they adsorb different isoforms of ApoE, an endogenous lipid‐based transporting protein. The protein pattern surrounding nanomaterials is disease‐specific and dependent on alterations of the human proteome, such as those occurring in several cancer types (Corbo et al., 2017; Hajipour et al., 2015).

Corona proteins behaving as opsonins mediate nanocarrier recognition by MPS and rapid clearance from circulation, whereas dysopsonins prolong the circulation time (Papini et al., 2020). Immunoglobulins and complement proteins (C3b/iC3b) cause the opsonization of NPs that predisposes them to recognition by circulating monocytes and mononuclear phagocytes, such as tissue‐resident macrophages, including hepatic Kupffer cells and rapid nanocarrier clearance (Moghimi et al., 2023). Nano‐bio interface controls not only nanocarrier PKs but also dynamically influences subsequent responses by the immune system (Corbo et al., 2016). The uncontrolled liberation of some complement mediators (C3a, C4a, and C5a) can induce an uncontrolled activation of complement cascade, leading to proinflammatory responses and anaphylaxis (complement activation‐related pseudoallergy (CARPA); Lee et al., 2015; Moghimi et al., 2023; Szebeni, 2014). Furthermore, the role of complement can be exacerbated in some diseases. In cancer, the local production and increased activation of complement in the tumor microenvironment that is associated with the modulation of antitumor immune responses can even promote tumor growth in both mouse models and cancer patients (Reis et al., 2018). The impact of complement activation and generation of complement effectors in the context of cancer has been recently reviewed (La‐Beck et al., 2021).

The interaction between nanocarriers and cellular constituents of human blood is often overlooked in the literature. However, it has implications not only for controlling nanomedicine biodistribution and therapeutic efficacy but also for safety reasons. For instance, neutrophils make up 60%–70% of white blood cells (WBCs) in human blood, unlike in mice, where they make up only 20%–30%. Thus, neutrophils are the primary phagocytes encountered by IV‐injected nanomedicines in humans and, in principle, involved in their PKs behavior. Regarding toxicity, some NPs can, in principle, induce hemolysis, increase erythrocyte aggregation, or decrease erythrocyte deformability, preventing them from performing their primary functions in hemostasis (Pan et al., 2018). The generation of pro‐thrombotic surfaces for platelet adhesion can also occur (de la Harpe et al., 2019). Interaction of nanocarriers with platelets can begin a cascade of signals that eventually lead to fibrin cross‐linking and clot formation and, in parallel, interfere with the essential functions of leukocytes (Lazarovits et al., 2015).

In cancer patients, the basal hematological profile can be very scattered and variable along both chemotherapy and radiotherapy regimens, complicating the overall picture. For instance, in the case of breast cancer patients (*n* = 267), RBC parameters, such as RBC count and mean cell volume, hematocrit, and hemoglobin levels, are significantly reduced from the initiation to the completion of breast cancer treatment. In contrast, the red cell distribution width (RDW) is increased considerably from pretreatment to posttreatment (Aynalem et al., 2022).

#### 4.2.2 Extravasation

The presence of a dysfunctional capillary bed with leaky endothelium and the absence of adequate lymphatic drainage in TME is at the basis of the Enhanced Permeability and Retention (EPR) effect, the mechanism historically evoked to explain extravasation of nanocarriers in solid tumors (Maeda, 2015; Matsumura & Maeda, 1986).

The molecular and functional heterogeneities of tumor tissues are important issues because the EPR effect is strictly dependent on tumor blood flow since no blood flow suggests a poor EPR effect or no EPR effect at all (Barua & Mitragotri, 2014; Fang et al., 2020; Maeda, 2021; Munir, 2022). Weakly perfused vessels and vessels even collapsed or obstructed blood by tumor‐associated fibroblasts or pericytes that adhere tightly to vascular walls, leading to a poor EPR effect and therapeutic failure (Fang et al., 2020; Nel et al., 2017; Prabhakar et al., 2013). A meta‐analysis of preclinical data suggested that a median of ca. 0.7% of the injected nanocarrier dose is accumulated in solid tumors. Further studies indicated larger values (Cheng et al., 2020) that, in any case, are higher than those found for many IV chemotherapeutics dominating the clinic (Rosenblum et al., 2018).

The mode of NP navigation in the blood and the lateral drift to endothelial walls (margination dynamics) further impact extravasation. Blood flow in the microvasculature is laminar, and nanocarriers, especially those spherical, follow the flow streamlines, making contact with the vascular wall unlikely (Gentile et al., 2008). Unique nanocarrier geometry can favor association with vessel walls, enabling extravasation through the fenestrated vasculature of tumors (Cooley et al., 2018; Decuzzi et al., 2009; Jurney et al., 2017; Lagarrigue et al., 2023), which makes the particle aspect‐ratio another parameter to consider for optimizing biodistribution and efficacy. Some strategies to enhance tumor accumulation of anticancer nanomedicines using EPR effect enhancers are on the way (Ding et al., 2019; Fang et al., 2020; Zi et al., 2022). Vascular disruption to facilitate extravasation of nanocarriers by using physical strategies (hyperthermia, radiation, or ultrasound) or chemicals (Combretastatin A‐4 phosphate, antiplatelet antibodies), as well as vascular normalization, has been proposed (Xiao et al., 2017).

The traditional EPR concept has also extended, evoking immune cell involvement, an active transport pathway comprising transcytosis in tumor‐associated endothelial cells, and the endothelial gap junction (Moghimi & Simberg, 2018). Neuropilin‐1 triggering of transcytosis has been postulated in orthotopic mouse models (Leng et al., 2017; Liu, Lin, et al., 2017). A study on gold NPs in the Zombie mouse model—tumor‐bearing mice perfused with a tissue fixative to deactivate any cellular activity while preserving vessel architecture—demonstrated that transcytosis prevailed on EPR or gap junction transport (Sindhwani et al., 2020). Whether such mechanisms also occur in human tumors is still to be proved.

The importance and the existence of the EPR effect in human patients have been heavily debated in the last 10 years (Danhier, 2016; Nichols & Bae, 2014; Zi et al., 2022) despite the evidence that it exists throughout different species (Gawali et al., 2023; Wu, 2021). Unfortunately, few studies systematically analyze the EPR effect and nanomedicine accumulation in human tumors. Tumor vessel functionality and gene expression often differ for different cancer subtypes, and vessel phenotype can be markedly heterogeneous within a single tumor (Kalyane et al., 2019; Lugano et al., 2020), making the extent of nanomedicines extravasation unpredictable. Nanocarrier transport through endothelial fenestrations is thus expected to be highly variable in patients. The implementation of strategies to stratify patients, predict response to treatment, and monitor the outcome is nowadays considered one of the prevailing challenges for the clinical application of anticancer nanomedicines (Bhatia et al., 2022; Dasgupta et al., 2020; Golombek et al., 2018). In a proof‐of‐concept study, a mathematical model was applied to evaluate chemotherapy concentration at the tumor site and its association with therapy response in colorectal cancer patients with liver metastases based on systemic drug pharmacokinetics, patient features, and tumor characteristics (Anaya et al., 2021). To inform the model, the authors estimated the local chemotherapeutic drug concentration at the tumor site using routinely measured clinical parameters (imaging, clinical and biologic parameters/markers, and chemotherapy regimen). They stratified the patients into responders and nonresponders to chemotherapy, enabling patient‐specific treatment protocols. In principle, extending this concept to nanomedicines may help predict therapeutic outcomes.

In a recent study, a nanoparticle biodistribution coefficient (NBC) was introduced to avoid the bias of drug release (Kumar et al., 2023). The analysis of literature data demonstrated that nanomedicines, independently of their composition, are initially found in the blood, liver, and spleen, then rapidly cleared from the blood and distributed to the intestine, and finally primarily accumulated in the liver and spleen. When dealing with polymeric NPs (size 10–190 nm), rapid uptake in the liver (32.1 %ID/g), spleen (13.3 %ID/g), tumor (3.2 %ID/g), and kidney (4.8 %ID/g) within the first 6 h occurs, followed by redistribution in liver (14.1 %ID/g) and spleen (9.4 %ID/g) followed by kidney (2.7 %ID/g) and GI tract (3.5 %ID/g) at later time points (Kumar et al., 2023). No final indication of the size effect of polymeric NPs could be withdrawn in the same study. Although tumor accumulation can be enough to treat small lesions (Anchordoquy et al., 2017), a crucial question to answer pertains to the fate of the nanocarrier and its cargo in TME and where the drug eventually interacts with the pharmacological target. In many studies, the extension of drug circulation time did not necessarily result in increased antitumor efficacy, whereas it was generally beneficial to reduce side effects (Table 2).

**TABLE 2:** Main studies reporting the pharmacokinetics of polyester NPs.

Polymer^a^	Drug	Preparation method	Nanocarrier (size)	Model/drug dose	PK parameters	Further details	Reference
PLGA (LA: GA = 75/25, MW = 10,000)	Paclitaxel (PTX)	Emulsification in an aqueous phase containing PVA or 2‐HP‐β‐CD	NPs PTX/PVA (164 nm) PTX/2‐HP‐β‐CD (149 nm)	KM mice PTX = 20 mg kg^−1^	*t*_1/2_α (h) Taxol® = 0.32 ± 0.10 PTX/PVA NPs = 0.20 ± 0.03 PTX/2‐HP‐β‐CD NPs = 0.27 ± 0.02 *t*_1/2_β (h) Taxol® = 1.43 ± 1.34 PTX/PVA NPs = 2.75 ± 1.43 PTX/2‐HP‐β‐CD NPs = 9.23 ± 3.58 AUC_0–∞_ (ng·L^−1^·h) Taxol® = 6383.63 ± 3022.91 PTX/PVA NPs 7092.40 ± 1520.21 PTX/2‐HP‐β‐CD NPs = 16466.73 ± 5189.89	Other PK parameters reported (*C*_max_, MRT, CL)	(Ma et al., 2023)
PLGA (MW = 30–60 kDa)	Methotrexate (MTX)	Emulsification in an aqueous phase containing PVA	NPs (164 nm)	Rats MTX = 5 mg kg^−1^	*t*_1/2_ (h) MTX = 1.56 ± 0.56 MTX‐NPs = 4.94 ± 0.15 AUC_0–∞_ (ng·mL^−1^·h) MTX = 375.46 ± 114.61 MTX NPs = 728.17 ± 84.37	Other PK parameters reported (*V*_d_, CL) Oral PK is also evaluated	(Jang et al., 2019)
PEG_2000_–PLGA (RG502H)	Betulinic acid (BA)	Emulsion in AP with PVA	NPs (148 nm)	BALB/c mice BA 10 mg kg^−1^	*t*_1/2_ (h) BA = 1.12 ± 0.28 BA‐NPs = 8.08 ± 1.23 AUC_0–∞_ (ng·mL^−1^ h) BA = 11,841 ± 1751 BA‐NPs = 19,232 ± 3582	Other PK parameters reported (*C*_max_) Increased efficacy of NPs versus free drug (Ehrlich ascites carcinoma (EAC) cells xenograft in Swiss)	(Saneja et al., 2017)
PEG_2000_–PDLLA_4000_	Docetaxel (DTX)	Thin‐film hydration	Micelles (25 nm)	Sprague–Dawley rats DTX 5 mg kg^−1^ DTX‐micelles 2.5–10 mg kg^−1^	*t*_1/2_ (h) DTX = 9.28 ± 1.8 DTX‐micelles 8.5 ± 1.35 AUC_0–∞_ (ng·mL^−1^ h) DTX = 972.25 ± 88.17 DTX‐micelles = 1140.22 ± 170.57	Other PK parameters reported (*C*_max_, *V*_d_, MRT, CL) The increase of micelle dose decreases *t*_1/2_	(Guo, Qin, et al., 2022)
PEG_2000_–PDLLA_4000_	Docetaxel (DTX)	Thin‐film hydration	Micelles (25 nm)	Beagle dogs DTX 1 mg kg^−1^	*t*_1/2_ (h) DTX = 19.45 ± 2.78 DTX‐micelles= 14.34 ± 8.04 AUC_0–∞_ (ng·mL^−1^ h) DTX = 913.21 ± 179.20 DTX‐micelles = 449.49 ± 115.9	Other PK parameters reported (*C*_max_, *V*_d_, MRT, CL)	(Guo, Qin, et al., 2022)
PEG_2000_–PLA_2400_ or PEG_2000_–PLA_2400_ PDLLA_10000_ (95:5)	TM2	Thin‐film hydration	TM2‐micelles (ca. 30 nm)	Male Sprague–Dawley rats TM2 = 8 mg kg^−1^	*t*_1/2_ (min) TM2‐micelles = ca. 30 min for both formulations TM2 = 3.2 ± 2.1 PEG_2000_–PLA_2400_= 3.2 ± 2.2 PEG_2000_–PLA_2400_ PDLLA_10000_ (95:5)= 7.2 ± 2.3 AUC (μg·L^−1^·h) TM2 = 2857.7 ± 990.6 PEG_2000_–PLA_2400_= 9648.3 ± 722.8 PEG_2000_–PLA_2400_ PDLLA_10000_ (95:5) = 13422.3 ± 2810.3	Enhanced core viscosity improves PK performance Other PK parameters reported (*C*_max_, MRT)	(Guo, Zhang, et al., 2022)
PEG_3000_–PLA_2000_ or Fol–PEG_3000_–PLA_2000_/PEG_2000_–PLA_2000_	Curcumin (CUR)	Thin film hydration	Micelles (ca. 70 nm)	Rats CUR = 5 mg kg^−1^	*t*_1/2_ (h) CUR = 0.09 ± 0.01 CUR‐micelles = 0.29 ± 0.02 CUR‐folate micelles = 0.32 ± 0.02 AUC_0–∞_ (μg L^−1^ h) CUR = 109.3 ± 5.9 CUR‐micelles = 834.5 ± 43.6 CUR‐folate micelles = 864.4 ± 124	Other PK parameters reported (MRT, *C*_max_)	(Yang et al., 2014)
PEG_5000_–PLA_5000_	Doxorubicin (DOX)	Nanoprecipitation	Micelles (20 nm)	BALB‐C mice DOX = 2.5 mg kg^−1^	*t*_1/2_ (min) DOX = 2.1 ± 0.6 DOX‐micelles = 12.4 ± 0.6 AUC (μg·min mL^−1^) DOX = 44.4 ± 4.6 DOX‐micelles = 908 ± 86	Other PK parameters reported (MRT, CL, *V*_ss_)	(Jiang et al., 2022)
PEG_5000_–PLA_2000_	Vorinostat (VOR)	Nanoprecipitation	Micelles VOR‐micelles 1:10 (81 nm) VOR‐micelles 1:15 (89 nm)	Rats VOR =10 mg kg^−1^	*t*_1/2_ (h) VOR = 0.61 0.61 ± 0.15 VOR‐micelles (1:10) = 54.46 ± 18.45 VOR‐micelles (1:15) = 112.71 ± 19.52 AUC_0–*∞*_ (μg h mL^−1^) VOR 2.48 ± 0.98 VOR‐micelles (1:10) = 6.77 ± 1.76 VOR‐micelles (1:15) = 39.01 ± 3.42	Other PK parameters reported (F, CL, CL renal and nonrenal, MRT, *V*_ss_) Drug:polymer ratio affects PKs PK after oral administration (50 mg kg^−1^)	(Mohamed et al., 2012)
PEO_5000_–PCL_10000_ PEO_5000_–PCL_16000_ PEO_5000_–PCL_16000_/PCL_30000_	Fluorescent dye (DiR)	Nanoprecipitation from different solvents	PEO_5000_–PCL_10000_ = worm (111 nm) and spherical NPs (70 nm) PEO_5000_–PCL_16000_ = spherical NPs (106 nm) PEO_5000_–PCL_16000_/PCL_30000_ = spherical NPs (313 nm)	Kras; p53 mutant allograft and autochthonous murine model of pancreatic ductal adenocarcinoma (hypoperfused)	*t*_1/2_ (h) PEO_5000_–PCL_10000_ worm = 6.6 ± 1.9 PEO_5000_–PCL_10000_ sphere = 16.6 ± 3.5 PEO_5000_–PCL_16000_ = 10.9 ± 1.0 PEO_5000_–PCL_16000_/PCL_30000_ = 12.9 ± 1.9	Relative tissue distribution and tumor penetration distance are independent of nanocarrier size or shape PoC with C_16_‐oxaliplatin(IV) prodrug	(Tao et al., 2018)
X‐PEG_5000_‐*b*‐PLGA_15800_ X = trastuzumab and panitumumab	Paclitaxel (PTX)/everolimus (EVER)	Nanoprecipitation	NPs (101 nm)	Healthy BALB/c female mice PTX = 15 mg kg^−1^ EVER = 7.5 mg kg^−1^	*t*_1/2_ (h) PTX = 1.88 EVER = 1.02 PTX/EVER‐NPs PTX = 5.17 EVER = 3.78 AUC_0–*∞*_ (μg·mL^−1^·h) PTX = 172.09 EVER = 84.88 PTX/EVER‐NPs PTX = 3671.59 EVER = 1814.83	Other PK parameters reported (*C*_max_, CL_tot_, *V*_d_) Dual‐NPs resulted in only marginal improvements in tumor growth inhibition and survival in MDA‐MB‐231‐H2N tumor‐bearing mice relative to the free‐drug combination. Reduction of PTX‐neurotoxicity	(Houdaihed et al., 2019)

Abbreviations: CL, drug clearance; MRT, mean residence time; *V*_ss_ = volume at steady state.

^a^ PEG is methoxy‐terminated unless otherwise specified.

### 4.3 Linking polyester nanoparticle design to biological behavior

#### 4.3.1 PEGylated nanoparticles

**FIGURE 5:**
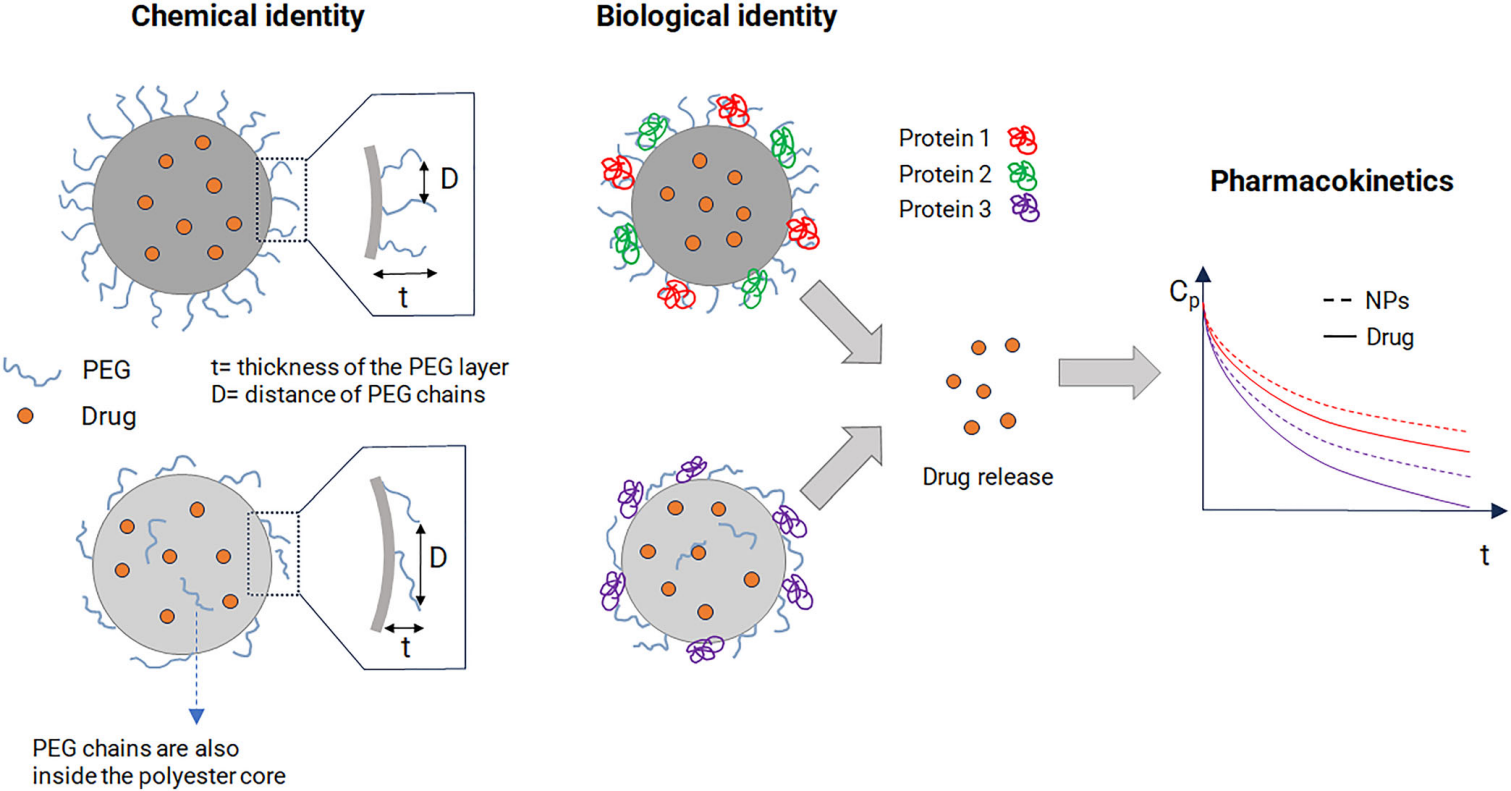
The chemical identity of PEGylated NPs is dependent on the PEG density on the surface which results in a distance between PEG chains (D) and PEG shell thickness (t). PEG density impacts protein corona formation and PK parameters, finally guiding NPs in specific body compartments. In parallel, premature drug release during circulation is expected to decrease NP‐enabled tumor accumulation.

The design of IV NPs is usually devoted to mitigating MPS recognition, making the nanovehicle invisible to the host's physiological clearance mechanisms and long‐circulating. Coating the surface of NPs with PEG is the gold standard for obtaining “stealth” NPs that avoid fast opsonization and MPS uptake. After the seminal work by Gref and coworkers who introduced the first PEGylated PLGA NPs (Gref et al., 1994), it became clear that PEGylation significantly increased the circulation time of NPs while reducing liver uptake compared to otherwise identical non‐PEGylated PLGA NPs. Subsequent studies showed that the half‐life of ~150 nm PLA–PEG NPs increased as PEG molecular weight increased (Bazile et al., 1995). These pioneering studies paved the way for using PEGylated NPs to deliver chemotherapeutics to solid tumors based on the concept that long‐circulating NPs have a higher probability of extravasating in tumors via the EPR effect. PEG with a molecular weight between 2 and 5 kDa with high density on the NP surface is considered suitable to shield the NP surface from opsonin adsorption, reduce recognition by the MPS, and extend circulation time (Gref et al., 2000). As illustrated in Figure 5, the distance between PEG chains (density of surface PEG) and thickness of the shell layer affects protein corona composition and, together with drug release during circulation, impacts PKs of PEGylated NPs.

We have collected the half‐life and AUC of the drug (free and loaded in different types of polyester NPs; Table 2). Although numerous studies have explored the preclinical biodistribution of NPs, most have concentrated on assessing the drug PK rather than the NP PK per se after single‐dose administration. Given that drugs may be released from NPs during circulation, the PK profile of the drug can highly differ from that of the NPs alone.

The topographical control of surface PEG opens new opportunities in long‐circulating cancer nanomedicines that deserve attention and further investigation. Hetero PEG brush surfaces composed of higher and lower molecular weight PEG chains demonstrated lower protein adsorption than homo PEG 2 and 5 kDa due to the increased PEG density and layer thickness (Matsumoto et al., 2014). A mushroom‐type PEG conformation regime prevails at low PEG density, whereas at high PEG density, the brush conformation regime allows chain extension and effective protein repulsion. PEG‐PCL NPs with varied surface PEG lengths (7.7–24.5 nm) but other unified properties, such as PEG density (ca. 0.16 and 0.4 PEG/nm^2^), particle size (100 nm), zeta‐potentials and shape, showed different biological behavior (Wang, Du, et al., 2018). One NP variant (indicated as 5000 T/F) exhibited a dramatically extended circulation time in plasma and higher accumulation in tumor tissue, as well as enhanced uptake in tumor cells. This prototype formulation delivering docetaxel as a model drug could significantly inhibit the growth of an MDA‐MB‐231 tumor xenograft in mice and prolong animal survival. PEG‐PLGA NPs with a hierarchical PEG shell consisting of a primary layer of PEG 5 kDa and a second layer of PEG 2 kDa covalently linked to the first PEG layer highlighted the role of topographical PEG distribution on NP circulation time and accumulation in the liver (Zhou et al., 2018). Surprisingly, the reduction of liver sinusoidal endothelial cell uptake rather than macrophage uptake was sufficient to dramatically extend NP blood circulation time.

In line with all nanomaterials, IV polyester NPs acquire a typical protein corona once in contact with blood, which strictly depends on their physicochemical properties. Protein corona can unpredictably change NP outcomes, including function, uptake, biodistribution, immunological responses, and toxicity, making the biological identity of NPs often elusive (Mahmoudi et al., 2023). In a recent overview of “nanoparticle protein corona” literature, only 68 of the 1073 most relevant published papers refer to PLGA, PLLA, or PCL NPs (Hajipour et al., 2023). Since protein corona compositional information is expected to guide therapeutic NP design, as in the case of lipid NPs, prospectively controlling its formation on appropriately tailored NPs is paramount.

PEGylated NPs were found to acquire mainly complement proteins in human plasma, not prominent on bare particles, resulting in their preferential uptake by neutrophils in human blood (Kelley et al., 2018). Strategies for recruiting NP‐engulfed neutrophils in the tumor were explored to accomplish higher drug levels at the tumor level. For instance, locoregional tumor treatment with CXCL1‐loaded hydrogels was found effective in accumulating PLGA NPs in an immunocompetent c57bl/6 mouse model xenografted with B16/F10 cells using neutrophils as “trojan horse” (Hao et al., 2020).

Few studies have bridged PEG shell features, size, and protein corona composition to the in vivo behavior of polyester NPs. A smart tool to gain insight into these aspects is building libraries of NPs with different PEG densities and comparable sizes, mixing the pristine polyester and PEGylated copolymers with various PEG and polyester lengths. For instance, in vivo protein corona formation on NPs prepared from mixtures of mPEG‐PLGA and PLGA (PEG MW 5000 Da) with different sizes (55–140 nm) and PEG densities (10–50 PEG chains per 100 nm^2^) was found to change PKs (evaluated with ^14^C‐labeled NPs) after IV injection in healthy mice (Bertrand et al., 2017). The authors identified a PEG density threshold (20 PEG chains per 100 nm^2^) below which blood clearance is rapid, with further PEGylation beyond this value unable to significantly prolong the circulation times (measured for 6 h). The PEG density threshold corresponded to a distance between PEG chains of 2.5 nm and a PEG layer thickness of 10.6 nm, which provides dense brush conformation. This PEG density threshold remained similar for NPs with 55, 90, and 140 nm diameters, and the clearance trend was also confirmed in rats.

These findings suggested that the surface makeup of the NPs, and not their dimension, is responsible for removal from the bloodstream. Interestingly, instead of complement activation, apolipoproteins, and mainly apolipoprotein E, accountable for the trafficking of lipids in the bloodstream, were found to impact NP clearance. For this protein, NPs with higher PEG densities appear to have a lower relative abundance of adsorbed ApoE. Overall, the findings strongly support the direct involvement of interactions with the low‐density lipoprotein receptor (LDL‐R) in driving the PKs of NPs.

The impact of size on the extravasation of PEG‐NPs was evaluated in comparative studies with lipid‐polymer NPs of lecithin/DSPE‐PEG‐PLGA having comparable zeta potential (ca. −50 mV) and different mean sizes (39, 68, and 116 nm; Zhao et al., 2014). In a xenograft tumor model with low permeability (BxPC‐3 cells subcutaneously injected in Female BALB/c nude mice) and median diameter of vessel pores in the sinusoids around 110 nm, only variants smaller than 100 nm could easily pass through the vessel pores. The study also demonstrated that the smallest NPs were eliminated from the tumor through lymphatic drainage after the diffusion from capillaries. Due to relatively shallower diffusion, NPs with intermediate size displayed longer permanence in the tumor tissue, which could be relevant considering the sustained release of drug payload for polyester NPs.

The shape of PEGylated NPs affects circulation time and the extent of extravasation inside tumors. Self‐assembled PEG‐PCL filomicelles of block copolymers were proposed first 17 years ago by the group of Discher (Geng et al., 2007). They found that flexible filomicelles up to 8 μm long were not rapidly cleared and instead appeared to circulate longer than spherical nanoassemblies composed of similar PEG‐based diblock copolymers (Geng et al., 2007). In a first study, worm‐like micelles of PEG‐PCL 5 and 6.5 kDa, respectively, were superior to their spherical counterpart in delivering paclitaxel to A549 cancer cells (S. Cai et al., 2007). Fluorescent filomicelles were then found to persist in the blood circulation for at least 24 h, a value larger than that for spherical micelles, and accumulated less in major organs relative to more permeable tumors (Christian et al., 2009). Paclitaxel‐loaded filomicelles were found to nearly double the maximum tolerated drug dose in normal mice compared to spherical micelles. In tumor‐bearing mice, the higher drug dose produced more remarkable and sustained tumor shrinkage and cell apoptosis (Christian et al., 2009). The filomicelles could also load and release a drug combination of paclitaxel and retinoic acid that durably represses carcinomas in the liver and prolong survival (Nair et al., 2018).

Moving from mice to humans, the only data available on PKs of polyester NP stem from Genexol®‐PM, an injectable formulation of paclitaxel based on PEG–PLA (2 and 1.75 kDa) micelles that gained the first clinical approval in South Korea. Although over a dozen clinical studies have demonstrated that Genexol®‐PM exhibits a higher anticancer effect and lower toxicity than free Taxol® (a micelle formulation containing ethanol and the PEGylated surfactant polysorbate 20), the precise benefit of having PEG in the formulation was not clear. Genexol®‐PM shows shorter half‐lives but higher MTD than Taxol®. Despite a higher dose, Genexol®‐PM demonstrated superior response rates, a similar dose‐limiting toxicity profile, and reduced acute hypersensitivity reactions compared to Taxol® in patients with nonsmall cell lung, gastric and breast cancers. The fast clearance of the chemotherapeutic from the bloodstream was considered advantageous in limiting the adverse side effects of myelosuppression. These data unequivocally demonstrate that a quick disassembly in the blood of the nanocarrier provides only limited benefit. In 2015, Triolimus, a polymeric micelle formulation developed by Co‐D Therapeutics comprised of PEG–PLA micelles (M_N_ of PEG = 4200 and M_N_ of PLA = 1900) of ca 40 nm encapsulating a combination of PTX, the mTOR inhibitor rapamycin, and the Hsp90 inhibitor tanespimycin (17‐AAG) which exert synergistic activity (Shin et al., 2011), was granted orphan drug designation for the treatment of angiosarcoma (https://co-drx.com/pipeline) and is currently in late‐stage preclinical evaluation for the treatment of breast cancer (BC), NSCLC, and angiosarcoma.

A final aspect to consider is the hemocompatibility of NPs in humans, which requires more extensive evaluation than simple hemolysis tests (de la Harpe et al., 2019). It has been pointed out that peripheral blood immune cells (lymphocytes, NK cells, granulocytes, and monocytes) have a different sensitivity to polyester NPs. For instance, PEG‐PLGA NPs suppressed the proliferative function of lymphocytes and the killing activity of NK cells. Still, they stimulated phagocytic activity of granulocytes and monocytes and the respiratory burst of phagocytes (Tulinska et al., 2015). Cationic PLGA NPs, instead, induced a three‐fold increase of leukocyte elastase by interacting with cell membranes and elevating Ca^2+^ influx that led to degranulation and oxidative stress (Hwang et al., 2015). Even slight changes to existing and well‐studied NPs can result in an entirely new biological profile that must be evaluated case by case to draw a more accurate and complete picture of the impact of a specific engineering strategy (Vishnevskiy et al., 2021). Such an understanding goes beyond the borders of hemocompatibility to provide insightful information on NP behavior in humans.

#### 4.3.2 Functionalized NPs

One of the drawbacks of PEGylated NPs is that cell internalization is impaired, which decreases the delivery of the drug payload to intracellular targets. Functionalization of NPs with molecules recognizing a specific receptor overexpressed in the tumor tissue has been widely practiced for increasing drug selectivity and treatment efficacy. The presence of one or multiple surface recognition elements on NPs is expected to encourage their receptor‐mediated intracellular transport, promoting, at least in theory, specific NP accumulation in the target cell (Rosenblum et al., 2018). Upon interaction of ligand‐displaying NPs with the receptor, the uptake of NPs is activated through different mechanisms (phagocytosis, clathrin‐ or caveolae‐mediated endocytosis) depending on the targeting element and the cell type (Rennick et al., 2021). An array of ligands has been exposed on the surface of NPs to bind receptors on tumor cells, tumor microenvironment components, or tumor vasculature. Biotin, folic acid, aptamers, antibodies, and peptides are ligands most frequently used (Rezvantalab et al., 2018).

NP functionalization with ligands of surface receptors displayed by endothelial cells of defective tumor vasculature (i.e., RGD peptides to target integrin family) has been proposed to facilitate NP accumulation at the tumor site (Danhier et al., 2009; Graf et al., 2012; Sheth et al., 2021). Tissue homing of NPs was demonstrated for PLGA NPs bearing alendronate on the surface, which showed bone tropism and proposed for spatiotemporally controlled delivery of therapeutics to bone metastasis (Swami et al., 2014; Thamake et al., 2012). Analogously, zoledronate‐anchored PEG‐PLGA NPs showed prolonged blood circulation half‐life, reduced liver uptake, and significantly higher retention at the bone site (Ramanlal Chaudhari et al., 2012).

Due to the involvement of the lymphatic system in cancer metastasis formation, targeting lymphatics is considered an option to avoid cancer cells spreading in the body (Cote et al., 2019). Standard chemotherapies currently in place do not allow therapeutic concentrations to be reached in the lymphatic system, which is the further consequence of making the lymphatic system a reservoir for cancer cells. A study in breast cancer patients showed that carboplatin administered intravenously has low nodal accumulation (Chen et al., 2004). Analogously, small molecules administered subcutaneously have minimal lymphatic uptake (Supersaxo et al., 1990).

Targeting to lymphatics was attempted with PEG‐PLGA NPs (ca. 90 nm and slightly negative zeta potential) conjugated to a tumor lymphatics‐homing peptide (LyP‐1), showed a significantly higher distribution in metastatic lymph nodes compared to nontargeted NPs (Luo et al., 2010).

The modification of PLGA‐PEG with sulfates to actively target P‐selectin‐expressing cancers enhances the efficacy of combined therapies (Khoury et al., 2024). By targeting P‐selectin, which is expressed on activated endothelial cells at the tumor site and tumor cells, the NPs showed improved accumulation in 3D spheroids and P‐selectin‐expressing tissues, such as BRAF‐mutated melanomas and BRCA‐mutated breast cancers. This targeted approach significantly enhances in vivo efficacy and safety of the free drugs, advancing the co‐delivery of various anticancer drug combinations to P‐selectin‐expressing tumors. Moreover, this strategy shows promise for treating brain metastases, particularly those associated with triple‐negative breast cancer and other cancers prone to metastasizing to the brain.

Besides the need for a careful selection of the receptor to target and the ligand to display, the design of nanoplatform for precision delivery requires an appropriate method to functionalize NP for controlling the optimal number of ligand copies to display and, in parallel, both size and surface charge (Veiga et al., 2023). Geometry also plays a significant role in controlling the number of targeting moieties that nanocarriers can display at their surface and, consequently, the extent of internalization in targeted cells. Antibody‐decorated filomicelles based on PEG‐PCL copolymer, which recognize distinct endothelial surface molecules, adhered to endothelium with high specificity both in vitro and in vivo (Shuvaev et al., 2011). Analogously, anisotropic nanocarriers of PLGA, such as rods, elliptical disks, and barrels obtained by the membrane stretching method, were proposed for efficient antibody immobilization and delivery to breast cancer cells (Barua et al., 2013) and to encourage accumulation in endothelial cells (Kolhar et al., 2013).

When moving to animal studies, IV‐targeted NPs can become covered by a protein corona that may hide the targeting ligand and result in no relevant benefit in terms of biodistribution (Theek et al., 2014). The coupling of targeting ligands, particularly at high ligand densities, can also potentially reintroduce nonspecific interactions with blood components that PEG coatings are meant to reduce and elicit immune responses (Alkilany et al., 2019). Therefore, upon surface modification, circulation time is often decreased due to increased opsonization and recognition by the MPS.

For this ideal concept to be functional, diseased cells would have to express a unique receptor absent in healthy cells, which, unfortunately, is not always the case. It has been shown that receptor expression on the tumor cells can change over time. Next to pathway switching between different receptors, alternative receptors can be upregulated on the tumor cells (Morgillo & Lee, 2005). Additionally, the receptor expression between patients can be highly heterogeneous, translating into likely different therapeutic efficacies (Seoane & De Mattos‐Arruda, 2014). For some targeting agents, the abundance of endogenous ligands might compete with receptor interactions of targeted nanomedicine, stressing the importance of cell target choice.

BIND‐014 is the only targeted polyester nanoplatform tested in the clinic. Developed by BIND therapeutics, BIND‐014 is a PLA–PEG NP entrapping docetaxel decorated with S,S‐2‐[3‐[5‐amino‐1‐carboxypentyl]‐ureido]‐pentanedioic acid to target prostate‐specific membrane antigen (PSMA). A multicenter open‐label, phase II clinical trial of 42 chemotherapy‐naive patients with progressing castration‐resistant prostate cancer patients with metastasis demonstrated that the treatment with BIND‐014 (60 mg m^−2^) is active and well‐tolerated (Autio et al., 2018). It was suggested that patients likely to benefit from this treatment could be identified before treatment is initiated since antitumor activity might be related to PSMA expression levels on circulating tumor cells (CTCs). Clinical trials were discontinued primarily due to strategic decisions made by the company.

#### 4.3.3 Nanoparticle transport in tumor microenvironment and delivery to lymphatics

Improving NP transport in tumor tissues and overcoming TME barriers is crucial for cancer therapy. In vivo studies have demonstrated that most NPs are restricted near the perivascular region and trapped in the tumor ECM instead of diffusing successfully into the deep tumor area. Some major strategies to enhance the therapeutic efficacies of nanomedicines include degrading ECM components by physical (ultrasounds, hyperthermia) and biochemical methods (bromelain, MMP, hyaluronidase, and collagenase) or inhibiting its formation (Li, Zhang, et al., 2022). However, disruption of ECM helps relieve the solid stress by reopening the collapsed tumor vessels but has little or no effect on their leakiness. Similarly, vascular normalization is ineffective for the distribution of nano‐chemotherapeutics. Moreover, it cannot reduce solid stress and decompress collapsed tumor vessels due to rigid ECM or proliferating cells (Stylianopoulos & Jain, 2013).

In general, high penetration depth depends on the size (Wang et al., 2015), shape (Chauhan, Popović, et al., 2011), surface charge (Han et al., 2018), and rigidity (Guo et al., 2018) of NPs.

In a very insightful study, surface charge was found to critically affect tumor penetration and therapeutic efficacy of PEG–PLA NPs loaded with docetaxel. NPs were prepared by assembling PEG–PLA copolymers with varying lipid components (neutral, positive, and negative head groups) to modulate surface charge (Wang, Zuo, et al., 2016). The authors elegantly showed how positive NP variants accumulated in cancer cells upon in vivo treatment, penetrated deeply inside tumor tissue, and, although slightly inferior in blood circulation and tumor accumulation as compared to their neutral and anionic counterparts, were more effective in inhibiting tumor growth in subcutaneous MDA‐MB‐231, CT26, and BX‐PC3 tumor xenografts. Preferential tumor accumulation and desirable interstitial penetration were also demonstrated for PEG‐PLGA NPs coated with chitosan oligosaccharides (Wang, Chen, et al., 2016).

In a recent paper, the influence of tumor ECM physical properties on NP diffusion in human gastric cancer was rationalized using a simulation model based on ECM features (He et al., 2023). The authors developed an evaluation matrix to estimate and predict therapeutic effects from a transport perspective by providing a diffusion score according to ECM conditions. The evaluation matrix can be prospectively used in a personalized medicine approach to help clinicians evaluate NP efficiency early based on the tumor ECM physical trait.

Regarding drug release kinetics, the benefit of nanomedicines with sustained release of drug payload, as polyester NPs, on tumor penetration has not been evaluated in the long run.

#### 4.3.4 Critical aspects in animal studies

Looking at the whole picture, it is unequivocal that PEGylation is useful for technological reasons, that is, to avoid using surfactants during NP fabrication and reduce NP tendency to aggregate upon drying procedures. Nevertheless, while PEGylation of polyester nanocarriers is a design strategy that is somewhat effective in preclinical models, its actual utility in the clinic is still a matter of debate (Sheffey et al., 2022). A reflection on the interpretation of results achieved thus far is due since they will guide the design of the next generation of polyester NPs and their translation in the clinic.

Tuning surface PEG density and thickness by changing the PEG molecular weight of the amphiphilic block copolymer is not trivial at all. This strategy often assumes that PEG chains are quantitatively located on the NP surface, which is not always true. This has led to approximate and sometimes conflicting conclusions, especially in cancer applications.

The targeting ability of NPs is generally verified in vitro by comparing the uptake of bare vs targeted NPs in 2D cell cultures. Often carried out with protein‐deprived media, the impact of protein adsorption on the NP surface on ligand–receptor interactions is overlooked. Interestingly, the expression extent of the targeting receptor can favorably increase moving from 2D to 3D models, as demonstrated for the Cluster of Differentiation 44 (CD44) receptor in OVCAR8 (a 1500‐fold increase from 2D to 3D spheroids) (Singh et al., 2021).

The integrity of the surface PEG layer on biodegradable NPs during circulation is another fundamental aspect overlooked in the literature since the hydrolysis of the PEG‐polyester linkage (Figure 1) can play a role in protein corona composition and PKs. Although it is unclear if polyester NPs are chemically stable when circulating in the blood, some data on short PEG–PLA (mPEG_2000_–PLA_2500_) injected in rats show that most of the PEG polymers are eliminated from plasma within 48 h and 86% of PEG–PLA polymers are metabolized into PEG and then excreted in urine (Meng et al., 2018). In the case of PEGylated lipid‐PLGA NPs, recent findings suggest that the lipid‐PEG layer can gradually dissociate from the NP surface in the presence of serum albumin, resulting in a spontaneous carrier de‐PEGylation in the blood (Zhu et al., 2017).

Fundamental to the discovery and development of anticancer therapies is the ability to model tumor growth, recapitulate the principal elements of human disease, and demonstrate measurable effects of an anticancer drug. In the last two decades, there has been a shift to models based on human tumor xenografts implanted in immunocompromised mice, whether athymic mice (lacking T cells), SCID mice (lacking T and B cells), or Beige mice (lacking natural killer lymphocytes), the reason being that human tumors will be more predictive of the activity of new drugs in the clinic (La‐Beck & Gabizon, 2017). Whereas this is likely for low molecular weight drugs, for more complex systems such as nanomedicines, the risk of overlooking a critical interaction with the immune system may override any advantage that a human tumor model may offer over a syngeneic tumor.

The current animal models are considered not entirely suitable for studying the extent of tumor accumulation of nanotherapeutics and predicting the efficacy of nanomedicines in clinical cancers (Fang et al., 2020; Gawali et al., 2023; Maeda & Khatami, 2018). For example, the vascular permeability of human tumors is exaggerated in xenograft models (Fang et al., 2011), and this is considered at least one of the reasons why nanocarriers have excelled in preclinical models, while they have yet to find full clinical success in humans (Subhan et al., 2021; Zi et al., 2022). An extended cutaneous vascular network contributes to the high vascular density that increasingly promotes NP accumulation in mouse xenografts (Moghimi & Simberg, 2018). Besides high vascularization, mice models of solid tumors are genetically homogeneous, whereas cancers seen in clinical settings are highly variable in size and completely different in genetic backgrounds. Furthermore, tumor location (xenograft vs orthotopic) influences NP tumor accumulation, which is lower in immuno‐deficient mice compared to immuno‐competent mice (Subhan et al., 2021). A recent study demonstrated that the accumulation of IV micelles in an orthotopic, immunocompetent 4 T1 model was dose‐dependent and highly predictive of therapy outcome (Biancacci et al., 2022), thus unequivocally linking nanocarrier extravasation with anticancer efficacy. However, there is likely no ideal mouse model, and selection should consider the clinical immune characteristics of the host, the type of cancer, and the goal of the study. In general, better in vitro and ex vivo human cancer models should be established and exploited in order to mimic the clinical scenario following the quest for alternatives for animal experimentation that will also include the human immune system. These models are being developed as they are required in order to follow the Modernization Act 2.0 while they include perfusable vasculature and the full TME together with the immune cells and 3D extracellular matrix (Pozzi et al., 2021).

Biodistribution studies on prototypes selected based on in vitro results are often conducted in healthy animals before efficacy studies in tumor models. This usually jeopardizes the possibility of drawing general conclusions about the impact of PEGylation on NP accumulation in the diseased area. Equally, the bias of low complement levels in rodents compared to humans (Ong & Mattes, 1989) possibly explains why PEGylation generally works well in reducing phagocytosis in mice (Kelley et al., 2018). Furthermore, animal models such as immunodeficient mice bearing human xenografts do not recapitulate the effect of immune blood cells on NPs PKs. Thus, the impact of PEGylation under these conditions does not directly extrapolate to humans (Sheffey et al., 2022). The bottom line is that more attention should be paid to the distinct properties between humans and rodents when testing anticancer nanomedicine.

A comment on the methodologies to evaluate biodistribution in rodents is equally due, as recently pointed out (Skotland et al., 2022). The widespread fluorescence‐based whole‐body imaging of fluorescent NPs assumes that the fluorescent tracker represents NPs and that the fluorescence intensity is linearly related to the number of NPs and/or the tracker accumulated in the region of interest. Unfortunately, this is a bias in many studies, as demonstrated in an insightful paper on the biodistribution of DiR‐loaded PEGylated PLGA NPs (Meng et al., 2018). The authors highlight how fluorescence quenching, dequenching, and signal saturation arise at increasing dye content and local NP concentration, leading to conflicting and sometimes misleading interpretations of tumor accumulation. In general, using physically entrapped fluorescent tags should be done carefully since extensive leakage of the dye or NP‐cell membrane direct dye exchange can occur (Meng et al., 2018). The accurate determination of the fluorescent dye in the tissue, the use of Förster Resonance Energy Transfer (FRET) NPs, and the copolymer labeling can be orthogonally combined to provide robust results (Chen et al., 2019).

Besides fluorescence imaging, more advanced tools are needed to evaluate the PK of NPs. In this respect, novel methods to separate PEGylated NPs from the blood and overcome the limitations of the current isolation methods are vital. In a recent article, an immunoprecipitation method using PEG‐antibodies was proposed as a robust strategy to assess the fraction of free and “nanomaterial‐associated” (i.e., encapsulated) drug in vivo (up to 24 h; Hacene et al., 2021). Another alternative is the use of chemiluminescence probes instead of fluorescent ones that are masked by endogenous and autofluorescence (Blau et al., 2023).

### 4.4 Advances in nanoparticle design and delivery strategies

#### 4.4.1 Surface functionalization with cell recognition motifs

As mentioned above, research efforts in tumor‐targeted delivery are generally fixated on the concept that surface functionalization of the NPs with a motif recognizing an overexpressed receptor on cancer cells is an effective tool to improve tumor homing of the drug and increase its efficacy. The strategy is expanding to TME targeting (Adityan et al., 2020; Swetha et al., 2023; Yang et al., 2023) with promising results with nanocarriers engineered to target TAMs, especially in the context of immunotherapies (Li, He, et al., 2022; Xiang et al., 2021), CAFs (Li, Liu, et al., 2021; Wu et al., 2021), and CSS (Yang et al., 2020).

**FIGURE 6:**
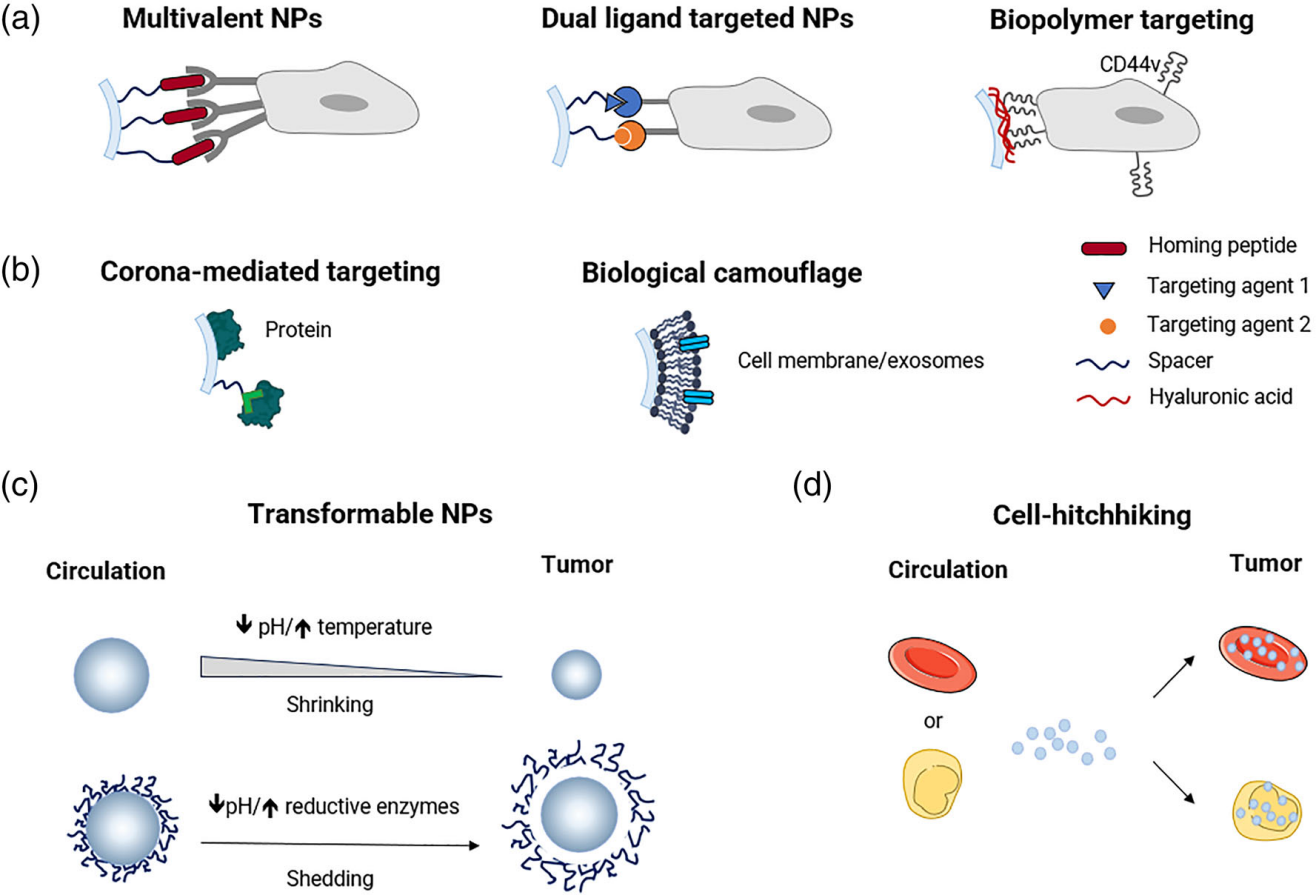
Advanced design of NPs for the delivery of chemotherapeutics. (a) Surface modification with low‐affinity binding ligands (i.e., peptides), dual ligands and biopolymers (hyaluronic acid); (b) controlled surface adsorption of a target protein and coating with cell membranes and exosomes of different origin; (c) NPs undergoing change of size or shell shedding in response to biological stimuli in the TME; (d) blood cell hitchhiking of NPs (surface adsorption or internalization). MSCs can be loaded with NPs ex vivo and then i.v. injected.

An excellent review has recently discussed new theoretical models and experimental methods that provide a quantitative view of cell targeting (Woythe et al., 2021). The authors focused on the advancements in multivalency theory that enable the rational design of super‐selective NPs. Particles with a higher ligand density can participate in more simultaneous ligand‐receptor bonds (Figure 6a). This leads to a more considerable permutation entropy contribution to their binding affinity and, thus, a stronger dependence on the density of receptors on the target. Therefore, high‐valence particles are more selective for surfaces with high receptor density while having low binding affinity for low‐receptor‐density surfaces. In general, greater flexibility affords multivalent particles to potentially have more simultaneous ligand‐receptor bonds with their target, enhancing their selectivity. Multivalent NPs functionalized with low‐affinity peptides can be helpful for targeting purposes due to multiple advantages (Murar et al., 2023; Seyyednia et al., 2021; Sun et al., 2018). Targeting peptides can be identified through powerful technologies like phage‐display, rationally designed by computational modeling, and easily manufactured by solid‐phase peptide synthesis (Fosgerau & Hoffmann, 2015). Peptide water solubility is high, vital to preserve the amphiphilic diblock nature of PEG‐polyester polymers and promote the correct location of the targeting element on the NP surface. As mentioned above, targeted NPs can confine their activity to perivascular regions of a tumor (binding site barrier) and paradoxically lose targeting ability in a biological environment due to interaction with proteins. This drawback is, however, less probable in the case of peptides due to their hydrophilicity. In one study, PLA–PEG copolymers consisting of carboxylic acid‐terminated short (2 kDa), medium (3.5 kDa), or long (5 kDa) PEG blocks and constant PLA blocks (10 kDa) were synthesized, and a cNGR peptide recognizing ανβ3 integrins covalently attached to the carboxylic acid function of the PEG chain. Short PEG_2k_ linkers encouraged the formation of colloidal clusters and cooperative binding to integrin receptors. In contrast, longer PEG chains (3.5 and 5 kDa) increased the chance of entangling and cloaking the ligand (Abstiens et al., 2019).

Peptides containing a CendR motif (RXXR) located at a C‐terminus can target NRP‐1, a nontyrosine kinase transmembrane receptor upregulated in several clinical disorders, including cancer (Jensen et al., 2015; Kadonosono et al., 2015). CendR peptides activate an endocytotic/exocytotic transport pathway that mediates penetration into the tumor matrix (Ruoslahti, 2017; Teesalu et al., 2009). iNGR peptides contain a cryptic tissue penetration CendR motif, a vascular homing motif, and a protease recognition site. iNGR initially binds the CD13 receptor on the endothelial cells and, upon proteolytic cleavage, forms a truncated peptide (iNGRt) that is recognized by NRP‐1 and undergoes the CendR pathway (Ruoslahti, 2017). We have adsorbed an iNGRt‐palmitoylated peptide on the surface of amine‐bearing PCL NPs, demonstrating its potential to selectively target and kill MDA‐MB231 triple‐negative breast cancer cells in vitro and in vivo (Conte et al., 2023). Peptide display can also be exploited to enhance the activity of peptides, as we demonstrated for an antiangiogenic peptide (aFLT1) covalently linked to the NP surface (Conte, Moret, et al., 2019). Since PEG functionalization with peptides can be challenging for cyclic and large peptides, alternative straightforward functionalization strategies for the noncovalent display of amphiphilic peptides have been proposed (Conte et al., 2023; da Silva Filho et al., 2021).

Due to the poor efficacy of single‐ligand nanomedicines, dual‐ligand targeting of polyester NPs is another promising option (Zhu et al., 2018) (Figure 6a).

As a multivalent biopolymer, coating NPs with Hyaluronan (HA; Figure 6a) holds great promise in targeted delivery. HA binds the CD44 variant isoforms (CD44v) abundantly expressed on the surface of cancer cells and tumorigenic cancer stem cells (Chen et al., 2018). HA can freely explore different conformations to maximize binding interactions with its target, promoting CD44 clustering (Rios de la Rosa et al., 2018; Rios de la Rosa et al., 2019). A significant relationship exists between increased CD44 expression and clinical indicators such as tumor grade and tumor cell differentiation in cancer patients (Chen et al., 2018). Apart from CD44, HA also interacts with CD44‐like receptors such as HA‐mediated motility receptor (RHAMM) and lymphatic vessel endothelial receptor‐1 (LYVE‐1), which are also overexpressed in tumor cells (Misra et al., 2015). Since tumor cells internalize faster than nontumor cells HA‐decorated NPs, this is promising as it suggests that nanocarriers may have limited off‐target effects (Rios de la Rosa et al., 2019). Inspired by layer‐by‐layer technology, we developed double‐coated NPs (dcNPs) with a PLGA core (negative), a bridging layer of polyethyleneimine (positive), and a low‐molecular‐weight HA coating (negative). dcNPs could be freeze‐dried, were highly stable in pharmaceutical vehicles and in the presence of plasma or FBS, accumulated selectively in CD44‐overexpressing A549 (Maiolino, Russo, et al., 2015), HCT‐116 (Russo et al., 2016), MDAMB‐231 cells (Maiolino, Moret, et al., 2015) and in CSSs from different breast cancer cell lines (Gaio et al., 2020). Competition experiments in the presence of free HA demonstrated that cell uptake occurred via receptor‐mediated endocytosis. HA‐coated PLGA nanoparticulate entrapping docetaxel (DTX‐HPLGA) exhibited prolonged circulation time, a remarkably high accumulation in the lung of nude mice, and effective antitumor activity in an orthotopic human lung cancer model in nude mice, resulting in significantly improved survival rates and reduced systemic side effects as compared to free DTX (Wu et al., 2017). HA‐decorated DOTAP–PLGA hybrid NPs delivering the HSP90 inhibitor 17AAG were found to enhance the therapeutic efficiency of the free drug in a Luc‐HT29 subcutaneous xenograft and AOM/DSS‐induced orthotopic tumor model (Pan et al., 2021). Furthermore, when administered for 7 consecutive days, spleen/body weight, cytokine‐expression levels, H&E staining of vital organs, and blood hemo and biochemical analysis demonstrated their biocompatibility.

Another approach to target cancer cells relies on the high affinity of cationic materials toward cancer cells. The elevated glycolysis in different cancer cell lines leads to a higher level of lactate secretion than in normal cells, generating negative cell membranes (B. Chen et al., 2016). Pioneering studies have shown that PEGylated cationic liposomes demonstrate superior effectiveness in targeting tumor versus normal vascular networks (Campbell et al., 2002). The issue with cationic NPs is their propensity to adsorb a large amount of negatively charged serum proteins, resulting in increased particle size followed by agglomeration or precipitation in the circulation, as well as stimulation of a robust immune response, making easy their fast clearance in the blood circulation system.

Recently, we explored the impact of amine groups on the properties of PEGylated NPs prepared from blends of an amino‐terminated PCL and mPEG‐PCL of different PEG lengths (Am/PEG‐NPs) or bare mPEG‐PCL NPs (PEG‐NPs; Conte, Dal Poggetto, et al., 2019; Esposito et al., 2018). We found that the shell thickness of Am/PEG‐NPs (positive charge) was increased compared to the PEG‐NPs variant (slight negative charge), likely due to the PEG chain extension operated by amine groups. Both Am/PEG‐NPs and PEG‐NPs changed their chemical identity in human plasma, becoming neutral due to protein adsorption. However, Am/PEG‐NPs internalized less than the corresponding PEGylated counterpart in human monocytes (Conte, Dal Poggetto, et al., 2019). Am/PEG‐NPs accumulated in the lung, whereas the variant with adsorbed HA (shielding amine groups) accumulated mainly in the liver in a metastatic lung mice model (IV injection of B16F10 murine melanoma cells; Esposito et al., 2018), demonstrating that the HA coating of Am/PEG‐NPs alters their biodistribution. Amine‐NPs functionalized with an iNGR peptide were efficient in targeting NRP1‐overexpressing MDA‐MB‐231 cells despite adsorbing proteins, exerting comparable cytotoxicity as free docetaxel, and attenuating free drug cytotoxicity toward human fibroblasts. When tested in mice bearing a subcutaneous MDA‐MB‐231 tumor, a slight but significant increase in antitumor activity and mice survival as compared with the nonfunctionalized variant and docetaxel in the vehicle of Taxotere® was observed (Conte et al., 2023). Even though the biological behavior of positively charged NPs has been somewhat contradictory, only further studies examining coherent libraries of positive NPs can establish their real potential as cancer nanomedicines. Besides, their easy preparation and the large availability of ionizable lipids to generate amine surfaces can strive for novel delivery concepts.

#### 4.4.2 Biological camouflage

Corona‐mediated targeting is an innovative approach where specific blood proteins are adsorbed on NPs to redirect them to specific locations in the body (Figure 6b). Strategies for the chemical and in situ immobilization of ApoE on the surface of NPs with different compositions can thus offer a tool to reroute nanocarriers to alternate receptors (Hartl et al., 2023). On some occasions, the adsorption of specific proteins can be intentionally promoted to recruit plasma proteins with targeting ability toward particular body compartments. It is also possible to precoat NPs with specific proteins to achieve a more precise functionalization and biological behavior. In this case, the exposure of functional binding motifs of adsorbed proteins is a critical requirement to take advantage of their recognition properties (Mahmoudi et al., 2023). In a recent example, surface modification of liposomes with a short peptide promoted the display of receptor‐binding domain of exchangeable apolipoproteins, allowing brain targeting (Zhang et al., 2019).

Cell membrane‐cloaked (or coated or camouflaged) NPs (cm@NPs) are an emerging class of nanoplatforms that integrate the functionality of biological materials with the flexibility of synthetic materials to achieve effective navigation and interfacing in complex biological systems. They consist of a synthetic core camouflaged by a cell membrane layer, which confers cell‐mimicking properties to NPs and mainly immune escape and tropism for the cell native tissue (Figure 6b). Nevertheless, cell membranes can be an antigen source and impart immunostimulatory features to the nanoplatform they cloak (Fang et al., 2023). Given the complexity of mass transport in the vascular compartment, it is not surprising that “biomimetic camouflage” strategies are gaining popularity (Fang et al., 2023; Li et al., 2018; Mohammadi et al., 2019; Yoo et al., 2011).

Red blood cells (RBCs) cm@NPs of PLGA are one of the first reported cell membrane‐coated systems conceived to escape macrophage recognition and body clearance and achieve long‐circulation (Hu et al., 2011). RBCs are homologous with the autologous immune cells and are cleared by the immune systems until damaged or dead (Elward & Gasque, 2003). Due to abundant self‐markers (CD47 proteins on the surface of healthy RBC acting as “do not eat me signal”), RBCs are recognized as a self‐component and circulate for about 40 days in mice and 3 months in the human body (Oldenborg et al., 2000). Thus, RBC cm@NPs escape from the recognition of the immune system, greatly extending their circulation half‐life (Hu et al., 2011). Since RBCs do not have a nucleus and lack organelles, RBC membrane vesicles are relatively easy to obtain by removing the hemoglobin content under hypotonic conditions and, after purification, are placed on the NP cores. RBC cm@NPs of PLGA loading doxorubicin (DOX) exhibited significantly increased inhibition of tumor growth compared with free DOX in an EL4 cell xenograft in C57BL/6 mice and excellent immunocompatibility (Luk et al., 2016). When the RBC membrane coats anisotropic NPs, superiorities in prolonged circulation time and reduced elimination from the body are found over those with spherical shapes (Ben‐Akiva et al., 2020). The implications of RBC‐coated NPs on interactions with immune cells recognizing CD47 receptor (SIRPa–CD47 innate immune checkpoint, for instance) will be a crucial point to address in the perspective of further development of the technology. CD47 is indeed overexpressed on the surface of various cancer cells and correlates with poor prognosis for several cancer types (Huang et al., 2022). Particle anisotropy was hypothesized to synergize with the biomimetic RBC membrane coating to achieve a more favorable PK. The rate of systemic elimination upon IV administration was reduced in the case of prolate ellipsoidal geometry (Ben‐Akiva et al., 2020). Although RBCs enable extended circulation time, they lack specificity and active targeting capacity for solid tumors, which would limit their application in cancer. To overcome this drawback, targeting ligands covalently linked to phospholipids can be post‐inserted in the RBC membrane to recognize target receptors efficiently (Fang et al., 2013). This strategy was employed to modify RBC membranes with DSPE‐PEG‐Man to target therapeutic cargo in hepatocellular carcinoma (Han, Bi, et al., 2022).

The half‐life of RBC cm@NPs in preclinical models is still far less than that of the original natural cells. Why this happens has not been understood yet. An aspect that can be relevant is the mode of cell membrane integration on the NP surface. The RBC membrane undergoes a series of processes, including co‐extrusion and sonication that can degrade the protein components displayed by cm@NPs. Finally, although translating RBC cm@NPs to human use remains to be investigated, large‐scale synthesis of the particles should be feasible given the existing infrastructure for blood collection and transfusion and the maturing fabrication technologies for polymeric NPs.

Cell membrane coating technology to engineer PLGA NPs has significantly expanded to the use of membranes derived from other cells as platelets and white blood cells (Han, Bártolo, et al., 2022; Kang et al., 2017; Parodi et al., 2013; Wang et al., 2020). Extensive studies showed that the hemostatic properties of platelets crucially promote the metastatic progression of cancer in many different ways, contributing to tumor angiogenesis, assisting tumor survival in the bloodstream, and promoting tumor cell and vascular interactions (Gay & Felding‐Habermann, 2011; Yeini & Satchi‐Fainaro, 2022). The recognition and interaction between CTCs and platelets have drawn wide attention since they play a major role in the metastatic process. When tumor cells enter the bloodstream, they face challenges such as shear stress and immune response that can lead to their destruction. Metastatic cancer cells need platelets to aggregate around them to help cancer cells survive in the blood and spread to new tissues. While tumor cells harness such binding interactions to gain advantages for their metastasis, platelet cm@NPs are a versatile platform for broad cancer targeting and CTC detection (Wang et al., 2020). In the context of chemotherapy, platelet cm@NPs have shown prolonged circulation (Chi et al., 2019) reduced cellular uptake by macrophage‐like cells, no particle‐induced complement activation in autologous human plasma, enhanced targeting capabilities to injured vessels of tumors (Hu et al., 2015; Li, Chu, et al., 2021; Rao et al., 2017) and improved toxicity and efficacy as compared with the free chemotherapeutic (Chi et al., 2019). Advances in this delivery technology have been recently revised (Han, Bártolo, et al., 2022).

Leukocytes are white blood cells larger than RBCs that can do amoeboid movement, which enables them to easily migrate to and from the blood vessels to the extravascular tissues. Tumor cells produce various cytokines and chemokines that attract leukocytes (Chow & Luster, 2014), which makes leukocytes promising vehicles for tumor targeting because of their inflammatory chemotaxis. Evidence showed that neutrophils and monocytes possessed both a CTC and niche‐targeting property by the intrinsic cell adhesion molecules on membranes, such as the selectin family of proteins (Swierczak et al., 2015). A biomimetic vesicle, the leukosome, constituted by proteins derived from the leukocyte membrane integrated into a synthetic phospholipid bilayer through a combined bottom‐up and top‐down approach, was proposed as an alternative to the use of cell ghost (Maiolino, Moret, et al., 2015). Neutrophil membrane‐coated NPs can act for cancer metastasis prevention and therapy, targeting both CTCs and the premetastatic niche (Kang et al., 2017; Krishnamurthy et al., 2016). T‐Lymphocyte cm@NPs of PLGA have also shown enhanced localization at the tumor site after low‐dose irradiation, in analogy to cytotoxic CD8+ T cells, likely due to upregulation of the expression of adhesion molecules in tumor vessels (Zhang et al., 2017).

Coating with macrophage cell membranes confers extended circulation properties to NPs and enhances their tumor‐targeting ability through the identification function of macrophage membranes (Xuan et al., 2015). Upon fulfilling its tumor‐homing and RES evasion mission, the macrophage‐membrane coating can be shed via morphological changes driven by extracellular microenvironment stimuli (Zhang et al., 2019). Besides homing to inflammation sites, macrophages could also actively bind to cancer cells via interactions on membrane receptors (Qian & Pollard, 2010). Macrophage cm@NPs are expected to possess the capability to cross vascular barriers and molecular recognition ability on metastatic tumor cells through functional proteins residing on the membranes (Cao et al., 2016).

Cancer cell membranes also show potential in biological camouflage (Chen et al., 2016; Fang et al., 2014; Jin et al., 2019; Kroll et al., 2017; Yang et al., 2018). Cancer cells possess various beneficial properties, such as limitless replicative potential, immune escape, and homologous targeting abilities (Hanahan & Weinberg, 2011). Cancer cm@NPs are expected to recapitulate some of the biological complexities of the cancer cell membrane and provide a one‐step solution to transferring some of the bioactive functions of the cell membrane to the carrier (bioinspired tumor self‐targeting). Disguised as autologous cells through the CD47 protein, cancer cm@NPs escape immune surveillance (Jia et al., 2021) and prolong circulation time. The display of homologous adhesion molecules (CD326) is considered relevant to favor accumulation in tumor tissue, although the mechanism of homotypic affinity is not completely clear. Remarkably, self‐recognition of the same cancer cell lines leads to the self‐targeting of the homologous tumor in vivo, which occurs even when competing with a heterotypic tumor (Zhu et al., 2016). Cancer cm@NPs targeting various tumors is achieved by adjusting the cell membrane source (Li et al., 2023; Liao et al., 2020). Tumor cells readily agglomerate, forming a solid mass owing to the presence of specific proteins (focal adhesion proteins, integrin, focal adhesion kinase, and RHO family proteins; Janiszewska et al., 2020; Maziveyi & Alahari, 2017). Downregulation of these proteins is involved in the translocation of cells to secondary metastatic sites. PLGA NPs coated with cell membranes from U87‐CXCR4 (a U87 cell line stably transfected with human CXCR4) disrupted the migration of cancer cells toward fibroblasts and significantly reduced metastatic burden (Jin et al., 2019). In an immunization protocol, these cancer cm@NPs migrated to proximal draining lymph nodes and induced the proliferation of CD8+ and CD4+ cytotoxic T‐lymphocyte populations in spleens and LNs. If the same binding occurs in vivo, is still a matter of debate. Several studies report as control uncoated NPs, which is insufficient to draw conclusive evidence.

It is worth highlighting that tumor cells cultured in the laboratory differ from in vivo tumor cells. Hence, the immune escape ability of tumor cell membrane‐coated NPs is sometimes limited (Liao et al., 2020). Integrating multiple cell membranes into hybrid membranes represents a promising approach to enhance the immune escape and the therapeutic outcome. Coating of PLGA NPs with a 143B‐RAW hybrid membrane (from human osteosarcoma cell line 143B and RAW264.7 murine macrophages) delivering paclitaxel had chemotactic effects on premetastatic niches. NPs exhibited superior targeting efficacy to the tumor site, inhibited tumor growth significantly, and showed lower toxicity than free paclitaxel in xenograft mice (Cai et al., 2022).

In cancer immunotherapy, cm@NPs are also expected to display membrane‐bound tumor‐associated antigens that, together with immunological adjuvants, can be efficiently delivered to professional antigen‐presenting cells (APCs) to promote anticancer immune responses. Core‐shell PLGA NPs, which carry the full array of cancer cell membrane antigens and incorporate an immunological adjuvant as Monophosphoryl lipid A (MPLA), promoted a tumor‐specific immune response for use in vaccine applications (Fang et al., 2014). In another example, an anticancer nanovaccine made of PLGA loaded with CpG oligodeoxynucleotide 1826 (CpG), a nucleic acid‐based immunological adjuvant stimulating TLR9, and a cancer cell membrane shell derived from B16‐F10 mouse melanoma cells resulted in a potent antitumor immune response in vivo and exhibited substantial therapeutic effects when combined with additional immunotherapies such as immune checkpoint blockades (Kroll et al., 2017). Murine melanoma cell line B16‐OVA cm@NPs loaded with the toll‐like receptor 7 agonist imiquimod (R837) and functionalized with mannose (post‐insertion of a mannosylated lipid) as APC‐recognition element demonstrated excellent efficacy to delay tumor development as a prevention vaccine and outstanding therapeutic efficacy to treat established tumors in combination with checkpoint‐blockade (Yang et al., 2018). Another example of a dendritic cell‐targeted mannosylated PLGA‐based nanovaccine showed that it synergizes with αPD‐1/αOX40 stimulates T‐cell infiltration into tumors and inhibits cancer progression (Conniot et al., 2019).

#### 4.4.3 Transformable NPs

Transformable nanocarriers are defined as systems that change their features along their journey in the body (Souri et al., 2022). Properties such as size, shape, and surface charge that are considered crucial in affecting PK, biodistribution, and efficacy, can optimize each delivery stage, overcoming the biological barriers imposed by the administration route (Figure 6c). The strategy generally requires ad hoc synthesized polyester block copolymers. In one of the first examples of transformable polyester NPs, a charge switchable system based on zwitterionic diblock copolymer of PCL (PCL‐*b*‐P(AEP‐g‐TMA/DMA)) showed prolonged circulation time in physiological conditions, as a result of the reduced nonspecific protein absorption of zwitterionic polymer, accumulated in the relatively acidic tumor tissue and then changed to positively charged for facilitated cellular uptake and enhanced therapeutic effect (Yuan et al., 2012). Shrinkable NPs of PLGA–PEG–PNIPAM responsive to pH and temperature decrease in size from 220 to 54 nm due to polymer degradation (Wang et al., 2019). In the planetary strategy, small nanocarriers are attached to the surface of large NPs by responsive binders, which can fall off in the presence of internal or external stimuli and then rapidly penetrate deep into the tumor. In one example, a small dendrigraft of poly‐l‐lysine (DGL, ~30 nm) linking doxorubicin was connected to PEG‐PCL micelles through a matrix metalloproteinases‐2 responsive peptide (Cun et al., 2018). These nanocarriers accumulate in the tumor and, under the influence of MMP2, release the drug‐bearing dendrigraft, reaching a high depth of the tumor.

Amid strategies to solve the “PEG dilemma”, PEG shedding after the arrival of the NPs in the TME has enormous potential (Fang et al., 2017; Kong et al., 2019) (Figure 6c). Different approaches have been used to design PEG‐sheddable NPs, such as degradation of the coating material (Lee et al., 2005) and cleavage of a chemical bond between the stabilizing polymer and its anchor (Romberg et al., 2008). In some recent strategies to achieve PEG shedding, NPs prepared with polymers where PEG is linked to the polyester with cleavable linkers that respond to specific stimuli as pH drop typical of chronic inflammation sites, reductive environments of tumors and high levels of metalloproteases have been reported. To exploit the drop of pH in the tumor microenvironment, NPs of PLGA‐grafted hyaluronic acid (HA‐g‐PLGA) were coated with a layer of methoxypoly(ethylene glycol)‐*b*‐poly(histamine methacrylamide) (mPEG‐*b*‐PHMA) copolymer via hydrophobic interactions (Wang et al., 2014). Upon the arrival of NPs at tumors, the PEGylated polymeric layer is conceived to detach from the NP surface due to the transition of PHMA blocks from hydrophobic to the hydrophilic state by extensive protonation of the imidazole groups in the weak acidic microenvironment of tumors. The naked HA shell promoted the cellular uptake of NPs by CD44‐presenting cancer cells and TAMs that shuttle them to the hypoxic regions where drug payload is delivered. Shedding of PEGylated NPs in response to high levels of metalloproteases is achieved by inserting protein‐cleavable short peptide sequences between PEG and polyester segment (Wang et al., 2014). To take advantage of the high GSH content, we synthesized a PEG–SS–PLGA polymer and prepared corresponding NPs demonstrating their superior accumulation in the core of HCT‐116 and A549 cancer cell spheroids as compared with the nonsheddable counterpart (Conte et al., 2018). This redox‐responsive PEG‐sheddable NPs delivering a siRNA/docetaxel combination showed higher cytotoxicity in A549 lung cancer cells than the PEGylated variant and were found effective in reducing the luminescence of A549‐luc lung tumor in mice after intratracheal administration (Conte et al., 2021). As mentioned, hybrid lipid‐polymeric NPs comprising PEGylated lipids undergo spontaneous PEG shedding in the blood (Zhu et al., 2017), which can be helpful to induce progressive PEG shedding during circulation.

It is worth underlining that in a significant number of reported dePEGylation strategies, PEG‐coated NPs are first taken up by target cancer cells, after which the low pH, reductive, and protease‐rich environment of the late endosome/lysosome trigger intracellular dePEGylation and drug release (Kong et al., 2019). However, these systems do not overcome the “PEG dilemma”, and the minimal uptake of PEGylated NPs remains a major drawback.

Very recently, coating of PEG‐PLGA NPs with imidazole‐based zwitterionic liquids has been proposed as a new potential platform to tune protein adsorption and increase NP affinity to RBC over platelets and white blood cells compared to unmodified PEG–PLGA control (Dasanayake et al., 2024).

#### 4.4.4 Cell‐hitchhiking

Hitchhiking is a concept of NP cruising on the surface of blood cells (Ding et al., 2018; Habibi et al., 2020; Stephan & Irvine, 2011). In this strategy, NPs leverage the intrinsic tropism of some circulating cells for tumors. Once hitchhiked by the cells, the therapeutic payload is offered long circulation and tumor homing (Figure 6d). RBCs, white blood cells, and stem cells are the most widely exploited for cell hitchhiking of NPs (Singh & Mitragotri, 2020). Some cells can also be genetically modified to add additional features (i.e., secretion of specific chemokines), potentially impacting tumor regression.

Total control over the NP loading of cells is a fundamental aspect of this technology. Ex vivo phagocytosis of NPs is the most direct approach for preparing cell‐based carriers, especially for immune cells with high phagocytic activity (Xue et al., 2017). For cells lacking phagocytic activity, NPs can be (1) adsorbed on NPs on the cell membrane, (2) covalently attached to the cell membrane, and (3) internalized ex vivo (Liu, Gao, et al., 2022).

Depending on the physical properties of NPs, cells can be attached to NPs through hydrophobic interactions, hydrogen bonding, and van der Waals forces. Positively charged NPs can be adsorbed onto anionic‐charged circulatory cells by ionic interactions. In this case, NPs are stably anchored on the cell surface under static conditions. Still, this binding is reversible, and NPs are disassembled when transporting cells due to physiological shear forces in blood circulation.

Antigen–antibody interaction, supramolecular host‐guest complexation, or covalent coupling can provide a more secure association. Ligand‐receptor interactions can be advantageously leveraged to stably adsorb NPs, as demonstrated in seminal works for CD44 receptors (Doshi et al., 2011; Swiston et al., 2008). In addition, avidin–biotin interactions were employed to obtain RBC‐PLGA NPs. Streptavidin–PEG_2000_–PLGA NPs entrapping vincristine were first anchored to RBCs, which were then endocytosed by monocytes/macrophages and recruited at the tumor level (Feng et al., 2021). NPs have also been stably coupled via covalent linkage with cells (Stephan et al., 2010; Wayteck et al., 2016). Advances in click chemistry in cancer cells via metabolic glycoengineering (Lee et al., 2019; Takayama et al., 2019) can open new avenues for applying the technology and ensuring cell integrity.

RBCs can load NPs through temporary permeabilization of the cell membrane by hypotonic hemolysis and subsequent isotonic resealing (Sun et al., 2015). RBC‐hitchhiking NPs (ex vivo adsorbed) transfer from RBCs to the first organ downstream of the intravascular injection (Brenner et al., 2018) and can, in principle, target nanocarriers to any organ by selecting the catheter injection site and increasing the local drug concentration by orders of magnitude (Brenner et al., 2021).

Monocyte hitchhiking was proven effective across several animal models and human glioblastoma, encouraged by the inflammatory response after partial postsurgical resection of glioma (Wu et al., 2018; Xue et al., 2017). Among the cells attracted to the TME, mesenchymal stem cells (MSCs) are considered another promising candidate (Aramini et al., 2022; Fan et al., 2023; Roger et al., 2010). Given the tropism of mesenchymal stem cells (MSCs) for brain tumors, for instance, a proof of principle of the concept in brain delivery was provided (Roger et al., 2010). MSCs, as cell hitchhikers of NPs, can relocate to regions of tissue injury and inflammation, such as tumors (Durymanov et al., 2015; Wang, Gao, et al., 2018). MSC engineering to produce proinflammatory cytokines but also to display homing factors can further encourage NPs homing at the tumor level and become a powerful therapeutic tool in cancer treatment (Choi et al., 2023; Golinelli et al., 2020; Spano et al., 2019).

Although cellular hitchhiking‐based delivery systems show prospects, they still face several challenges. Cell therapies are expensive, and the impact of cell modification can provide unexpected side effects. Complex and lengthy procedures impede the clinical application of such cell‐driven drug delivery technologies. Studies are limited to small animals, and it is uncertain if a translation to humans is feasible.

### 4.5 Alternatives to intravenous administration

The local administration of chemotherapeutics dates back 60 years and was proposed since the discovery of chemotherapy for cancers confined in a region of the body to avoid the barriers associated with transport processes (Budker et al., 2014). Local cancer treatment follows the concepts of (i) regional delivery solely to the specific affected organ or part of the body and (ii) intratumoral/intralesional delivery with precise administration. The support of a visualization technique is generally needed to determine where to place the injection needle.

**FIGURE 7:**
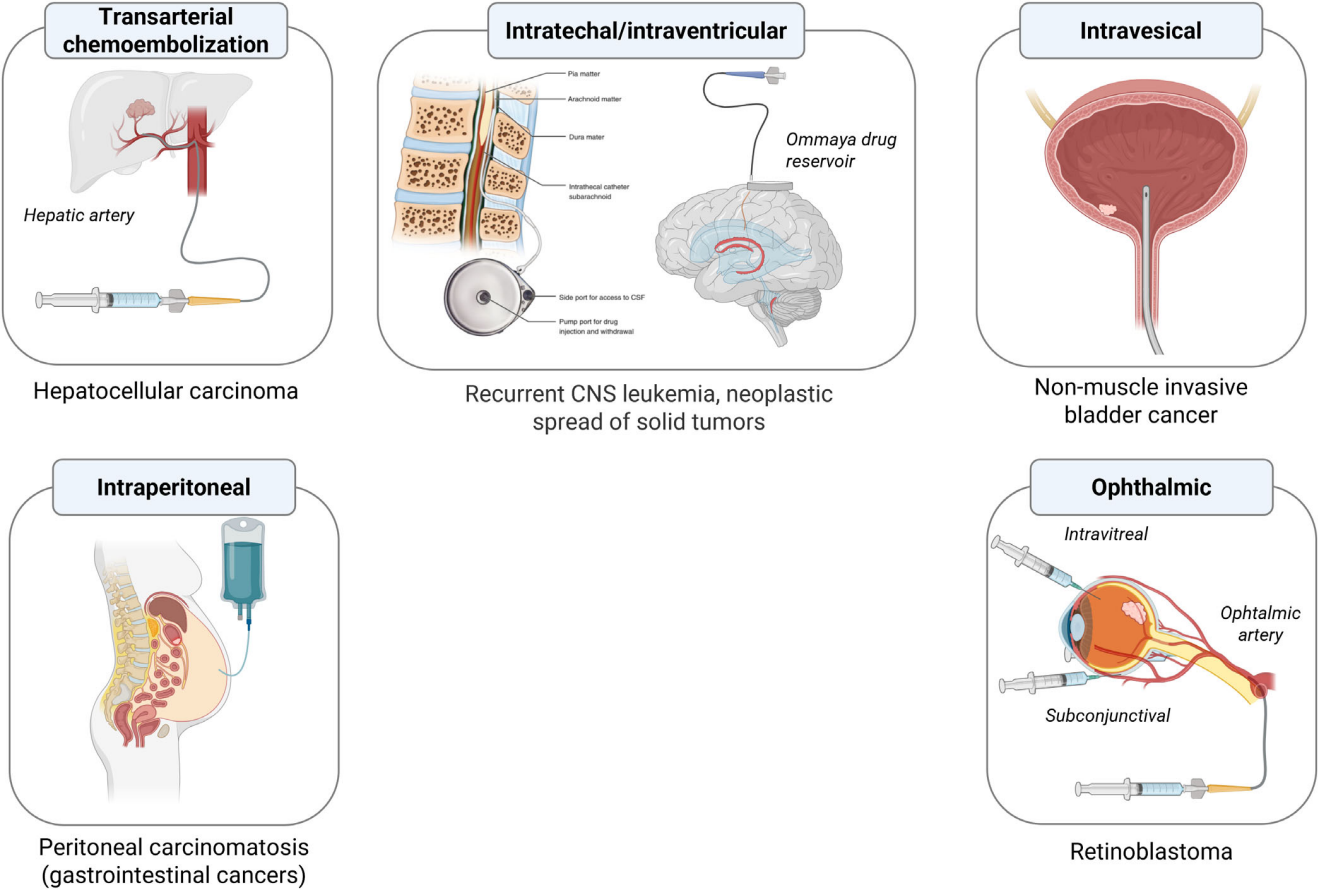
Loco‐regional administration routes are currently employed for the delivery of chemotherapeutics that can be exploited to administer NPs.

Loco‐regional therapy is possible across various clinical indications and has the great advantage of localizing drug payload, mainly at the tumor site, reducing off‐target and dose‐limiting toxicity (Figure 7). Transcatheter arterial chemoembolization (TACE) still plays a vital role in the treatment of hepatocellular carcinoma (Cardarelli‐Leite et al., 2020), intraperitoneal (i.p.) chemotherapy is particularly relevant in cases of peritoneal carcinomatosis (gastrointestinal cancers) and advanced ovarian cancer (Armstrong et al., 2006; Goodman et al., 2015; Shariati et al., 2020), intrathecal and intraventricular administration, that is, the direct delivery of chemotherapy into the cerebrospinal fluid, target malignancies involving the central nervous system (Gomes, 2022; Triarico et al., 2019), intravesical chemotherapy of superficial bladder cancer and nonmuscle‐invasive bladder cancer to help prevent recurrence or progression, topical treatment of skin cancer (Cullen et al., 2020). Intra‐arterial, intravitreal, and subconjunctival chemotherapy is an option in retinoblastoma (Gupta & Meena, 2020). Intratumoral injection of gemcitabine in pancreatic cancer patients was technically feasible, but therapeutic benefits have not been evaluated yet (Levy et al., 2017).

The persistence of the chemotherapeutic at the tumor site is often a critical aspect to address (Vogelbaum & Aghi, 2015), and the invasiveness of some local administration routes is also an essential aspect to consider. It is not coincidental that carmustin wafers (Gliadel®) were the first polymeric implant with sustained release (5 days in vivo) to be approved for glioblastoma.

With the advent of cancer immunotherapy, intratumoral delivery has been revamped and expanded tremendously (Melero et al., 2021). The approval of talimogene laherparepvec, an injectable modified oncolytic herpes virus being developed for intratumoral injection for localized melanoma, has prompted companies to initiate clinical programs to test intratumoral immunotherapies also for other cancer types (Melero et al., 2021). Although intralesional injection cannot directly target inaccessible metastasis and deep‐seated tumors, local delivery of immunotherapies initiates a host immune response and abscopal effects that induce the regression of distant untreated lesions (Terracciano et al., 2022).

Intratumoral delivery of monoclonal antibodies (Blanco et al., 2023) and CAR‐T cells (Sagnella et al., 2022) is nowadays considered an up‐and‐coming area in immunotherapy. Currently, 51 registered clinical trials are exploring locoregional CAR‐T cell delivery in solid tumors, of which 8 have been completed (Sagnella et al., 2022). The majority of these locoregional delivery trials have targeted tumors of the central nervous system (CNS) and liver. Nevertheless, cancers involving serosal surfaces, such as epithelial ovarian cancer with its transcoelomic pattern of spread, the pleura as in malignant mesothelioma, or locally resident head and neck cancers are theoretically more suited to local therapy than tumors with early metastatic spread, such as breast cancer (Sagnella et al., 2022).

Recently, some frontline chemotherapeutics have been found to additionally promote antitumor immunity by increasing tumor immunogenicity, improving T‐cell infiltration, or depleting the immunosuppressive populations (Kroemer et al., 2013; Li, Liu, et al., 2021). Some chemotherapeutic agents, such as cisplatin, induce immunogenic cancer cell death, including pyroptosis (Wang, Gao, et al., 2017) and ferroptosis (Zhang, Sui, et al., 2020), as well as release immunostimulating molecules. In vivo preclinical data shows that INT230‐6, an intratumoral formulation comprising a cell permeation enhancer (8‐((2‐hydroxybenzoyl)amino)octanoate) and a combination of cisplatin and vinblastine sulfate (Intensity Therapeutics), saturates and kills injected tumors and induces an adaptive (T‐cell mediated) immune response that attacks not only the injected tumor but also undetected micro‐metastases. The penetration enhancer facilitates the dispersion of the two drugs throughout injected tumors and enables increased diffusion into cancer cells. Animals become permanently immunized against the type of cancer that INT230‐6 eliminates. A nonrandomized Phase I/II clinical trial evaluating the intratumoral administration of escalating doses of INT230‐6 is being conducted in patients with several types of advanced refractory cancers (*n* = 110; NCT03058289). A phase II, randomized, multicenter, parallel design, window of opportunity trial evaluating intratumoral INT230‐6 in up to 90 patients with early‐stage breast cancer is currently open to recruitment (NCT04781725).

Nanotechnologies are expected to revolutionize local chemotherapy, although this field has not been largely explored. NPs of mPEG–PCL, COOH–PEG–PCL, or their mixture with a size of 50 nm and zeta potential of −6 (neutral), −19 (partially charged), and −37 mV (fully charged) were able to passively target the proximal (neutral variant) or distal (partially charged variant) lymph nodes after subcutaneous administration proximal to the site of the tumor (Doddapaneni et al., 2015). NPs of mPEG–PLA and mPEG‐PLA/PLA mixtures with size about 200 nm were subcutaneously injected in healthy mice and taken up by dendritic cells and macrophages in the draining lymph nodes, demonstrating their targeting potential (Widmer et al., 2018). PLGA NPs (163 nm, −18.5 mV) loaded with methotrexate were found to accumulate drug cargo in axillary and mesenteric lymph nodes after IV administration in Sprague–Dawley male rats (Jang et al., 2019).

Nanotax® and NanoDoce® are nanoformulations in Phase II clinical trials for intratumoral delivery in different cancer types (Rossi et al., 2020). Abraxane®, a nanoformulation of paclitaxel, was tested in patients with advanced malignancies that are primarily confined to the peritoneal cavity in a phase I trial, demonstrating a significant pharmacological advantage and promising clinical activity (Cristea et al., 2019). The efficacy of i.p. administration can be further enhanced by Pressurized Intraperitoneal Aerosol Chemotherapy (PIPAC), a novel delivery technique consisting of the administration of an aerosolized drug using a high‐pressure injector and atomizer that enhances drug penetration in tumor tissue due to the elevated i.p. pressure. This delivery modality has benefits in patients with peritoneal metastasis due to gynecological and gastrointestinal cancers. The specific development of i.p. However, NPs to deliver chemotherapeutics through peritoneal infusion/injection or PIPAC is in its infancy, with some preclinical results (Dakwar et al., 2017; Shariati et al., 2020).

Inhaled chemotherapy offers a promising alternative for certain lung cancers and metastatic respiratory diseases (Abdelaziz et al., 2018). Lung tumors or metastases can be exposed to the drug topically or through absorption into the bloodstream and drainage into the lymphatic system, leveraging favorable drug concentration gradients (Kosmidis et al., 2019; Wauthoz et al., 2021). While evaluated since 1968, limited clinical trials have explored inhaled cytotoxic chemotherapy (Celikoglu et al., 2008; Chou et al., 2013; Lemarie et al., 2011; Zarogoulidis et al., 2012). These trials highlighted advantages such as reduced systemic side effects but raised concerns about severe toxicities, especially in the pulmonary tract (Sardeli et al., 2020). Formulating drugs for inhalation remains challenging due to nebulizer limitations, leading to substantial aerosol loss due to exhalation and dose remaining in the device (Lavorini et al., 2019). The aerosolization of the medicine and possible operator exposure necessitates infrastructural requirements and hospital‐based administration (Darwiche et al., 2013). As nebulizers require an aqueous liquid formulation, some clinical trials have used the medicine used for the IV route directly, overlooking any rational development of the formulation. Furthermore, few studies have evaluated the impact of tumors (size, localization, complete, or partial conduit obstruction) and/or respiratory function on the deposition of aerosol particles in patients bearing a lung tumor. Despite these challenges, technological advances in pulmonary delivery offer opportunities for inhaled cytotoxic chemotherapy. Dry powder inhalers (DPIs) show promise in delivering large drug dosages, limiting drug exhalation, and avoiding air contamination. The powders to deliver need precise engineering to ensure efficient aerosolization, proper lung deposition, and release of the desired drug dose. Enhancing lung residence to avoid repeated administrations while preserving acceptable lung tolerance is crucial in formulation strategies (Darwiche et al., 2013) since both the drug and the excipients in the formulation might compromise pulmonary function. As novel excipients, polyesters necessitate rigorous evaluation for local tolerance profiles (Healy et al., 2014).

Inhaled cytotoxic chemotherapy may find applications in conventional treatment for selected patient subpopulations, considering tumor characteristics, clinical stage, cancer histology, lung capacity, and drug type. In combination with immune checkpoint inhibitors in advanced‐stage patients (unresectable‐stage III and stage IV NSCLC) and extensive‐stage SCLC, inhaled chemotherapy can become an alternative to systemic chemotherapy. Neoadjuvant or adjuvant chemotherapy in resected early‐stage NSCLC and treatment of pulmonary metastases (osteosarcoma) present intriguing possibilities (Wauthoz et al., 2021).

PEG–PLGA NPs loaded with paclitaxel with an average diameter of 70 nm were able to diffuse 100‐fold faster than similarly sized PLGA NPs, resulting in a delay of tumor growth Following Local Administration in a Malignant Glioma model (Nance et al., 2014). While the convection‐enhanced infusion of small molecules and NPs in the tumor mass has often led to contradictory results (Jahangiri et al., 2017; Song et al., 2017; Vogelbaum & Aghi, 2015), polymeric in situ forming implants, patches, and fibers continue to be actively explored in preclinical studies for the sustained release of a variety of chemotherapeutic (Talebian et al., 2018), and immunotherapeutic molecules (Wang, Wang, et al., 2018). Embedding drug‐loaded nanocarriers in thermosensitive hydrogels can improve NP local retention and extend the drug therapeutic window (Brachi et al., 2020). A conformable polymeric implant in the form of a micrometer‐sized mesh (μMESH) of poly(lactic‐co‐glycolic acid) laid over a water‐soluble poly(vinyl alcohol) pillars embedding taxane‐loaded PLGA NPs have been proposed as intracranial therapy against glioblastoma, suggesting that the sustained delivery for several weeks from a peritumoral position of small doses of potent chemotherapeutic molecules could halt the progression of aggressive brain tumors (Di Mascolo et al., 2023). Overall, loco‐regional chemotherapy alone or combined with immunotherapies via nanocarrier delivery holds great promise in expanding the therapeutical arsenal against difficult‐to‐treat cancers.

Oral chemotherapy is considered a game‐changer due to its convenience, and improved patient compliance nowadays limited to a narrow class of chemotherapeutics (e.g., tyrosine kinase inhibitors, etoposide, topotecan, temozolomide, vinorelbine, capecitabine). The oral delivery of most anticancer drugs is hindered by their low bioavailability, with only a tiny portion of the drug reaching the systemic circulation when administered orally. For instance, paclitaxel, docetaxel, and doxorubicin have demonstrated oral bioavailabilities of approximately 1%, less than 10%, and less than 5%, respectively (Kuppens et al., 2005). This limited availability is primarily due to the substantial first‐pass metabolism by the liver microsomal enzyme cytochrome P‐450, as well as their expulsion by the overexpressed plasma membrane transporter, including P‐glycoprotein (P‐gp) efflux pump. The CYP3A subfamily in the enterocytes is responsible for drug metabolism at the gastrointestinal tract wall (luminal metabolism), limiting bioavailability (Eisenmann et al., 2022). An oral PTX formulation (DHP107) composed of PTX, monoolein, tricaprylin, and Tween 80, developed by Daehwa Pharmaceutical Co., was approved in South Korea in September 2022 for the treatment of gastric cancer (Liporaxel®). Upon oral administration, it is postulated that this formulation interacts with bile acids and spontaneously forms “micelles” of about 10 μm in diameter in the intestine (Hong et al., 2007). A phase III trial to evaluate the efficacy and safety of DHP107 compared to Taxol (IV paclitaxel) as first‐line therapy in patients with recurrent or metastatic HER2‐negative breast cancer is ongoing (NCT03315364).

Besides solving stability issues, NP transport via M cells in Peyer's patches through the lymphatic system can thus become a pivotal mechanism to circumvent the efflux pump and ensure systemic delivery (Kim et al., 2018; Managuli et al., 2018). Gemcitabine‐loaded PLGA NPs showed a 21.47‐fold increase in oral bioavailability compared to a drug solution in rats (Joshi et al., 2014), which is promising. Nevertheless, contradictory results on the ability of PEGylated PLGA NPs to improve drug bioavailability are reported in the literature (Guo et al., 2017; Li et al., 2017; Liu, Wu, et al., 2017; Morelli et al., 2019).

Recently, a PLGA nanoformulation of rifampicin has been tested in humans, and a phase I ascending dose study is ongoing (Rather et al., 2023). It was observed that NPs accumulate in distal parts of the gut from where the encapsulated drug can be released in a sustained manner. More negligible NP absorption via the paracellular spaces from the GI tract is also hypothesized to explain the accumulation of rifampicin in the lymph nodes.

It is worth noting that self‐medication, although more convenient for patients, has multiple opportunities for variability and error. Poor adherence to an oral chemotherapy regimen may decrease the treatment effectiveness and even worsen survival. At the same time, toxic effects experienced by patients in routine practice may be greater than those initially reported in clinical trials (Di Maio et al., 2016).

The presence of peculiar barriers imposed by each delivery route cited above is a crucial aspect of selecting design criteria for polyester NPs and allowing their smooth development (Wang et al., 2024).

## 5 CONCLUSIONS

Looking back, seven decades of efforts have produced stunning drug delivery technologies to break down the barriers between new drug candidates and their targets in tissues and cells. The first generation of cancer nanomedicines, primarily intended to reduce the severe toxicity of IV chemotherapy and avoid using toxic vehicles, still represent the most significant proportion of nanomedicines in the market.

Although potentially providing significant therapeutic advantages for many medical applications, nanomedicine translation has not progressed as rapidly as the plethora of positive preclinical results would have suggested. The discouraging clinical results reported for cancer nanomedicines intended to be superior to commercial products in terms of efficacy and improved survival have underscored the need to revise their development strategy. In the context of polyester NPs, Genexol®‐PM is a stand‐alone product based on polyester micelles approved in South Korea, raising the question beyond such a mismatch between performance in mice and their almost absence in the clinic. Surprisingly, the sustained release features of polymeric NPs have not been fully exploited in the context of cancer chemotherapy.

It is difficult to pinpoint precisely where the issue lies, or which factors are responsible. Despite several advantages, the application of polyester‐based NPs suffers from several limitations, partly shared by other nanomedicines. We envision that conducting thorough preclinical evaluations is crucial to identify potential issues early on. Gaining a deeper understanding of the PKs and pharmacodynamics of NPs is vital for optimizing dosing regimens and achieving desired therapeutic outcomes. This involves adopting robust animal models and analytics to study the biodistribution, clearance mechanisms, and interaction of NPs with biological systems to inform dosing strategies and minimize toxicity. For instance, it is surprising that the impact of continuous exposure of a tumor to low drug amounts realized with slow‐releasing polyester NPs is far from being addressed in clinically relevant conditions. Nevertheless, the persistency of NPs in the tumor has not been fully elucidated. The selection of tailored NPs for a chemotherapeutic should be considered case‐by‐case as dictated by physicochemical and pharmacological behavior. A change in drug biodistribution, for instance, can be relevant for chemotherapeutics with tissue‐specific toxicity (as in the case of Doxorubicin) and to reduce dose‐limiting toxicity (DLT) but worthless for another chemotherapeutic.

From a technological perspective, comprehensive physicochemical characterization of NPs in conditions mimicking biological environments should allow a better understanding of their biological behavior. Developing scalable and reproducible manufacturing processes is essential to ensure consistent quality and batch‐to‐batch reproducibility of NPs. A promise comes from advanced manufacturing techniques, such as continuous manufacturing, that can enhance control over critical quality parameters and reduce batch‐to‐batch variability. Due to their biodegradability, polyester NPs should be formulated as solid products, which is not easy.

AI and ML are expected to facilitate the prediction of drug release kinetics, stability, and interactions with biological systems, allowing for informed decision‐making early in the development process. To this purpose, the power of High Throughput Formulation Screening platforms and the vast amount of data collected on polyester NPs may provide massive output data that can feed predictive models. From a regulatory standpoint, adhering to quality guidelines and ensuring compliance from the early stages of NP development can minimize regulatory hurdles and delays in the approval process.

**FIGURE 8:**
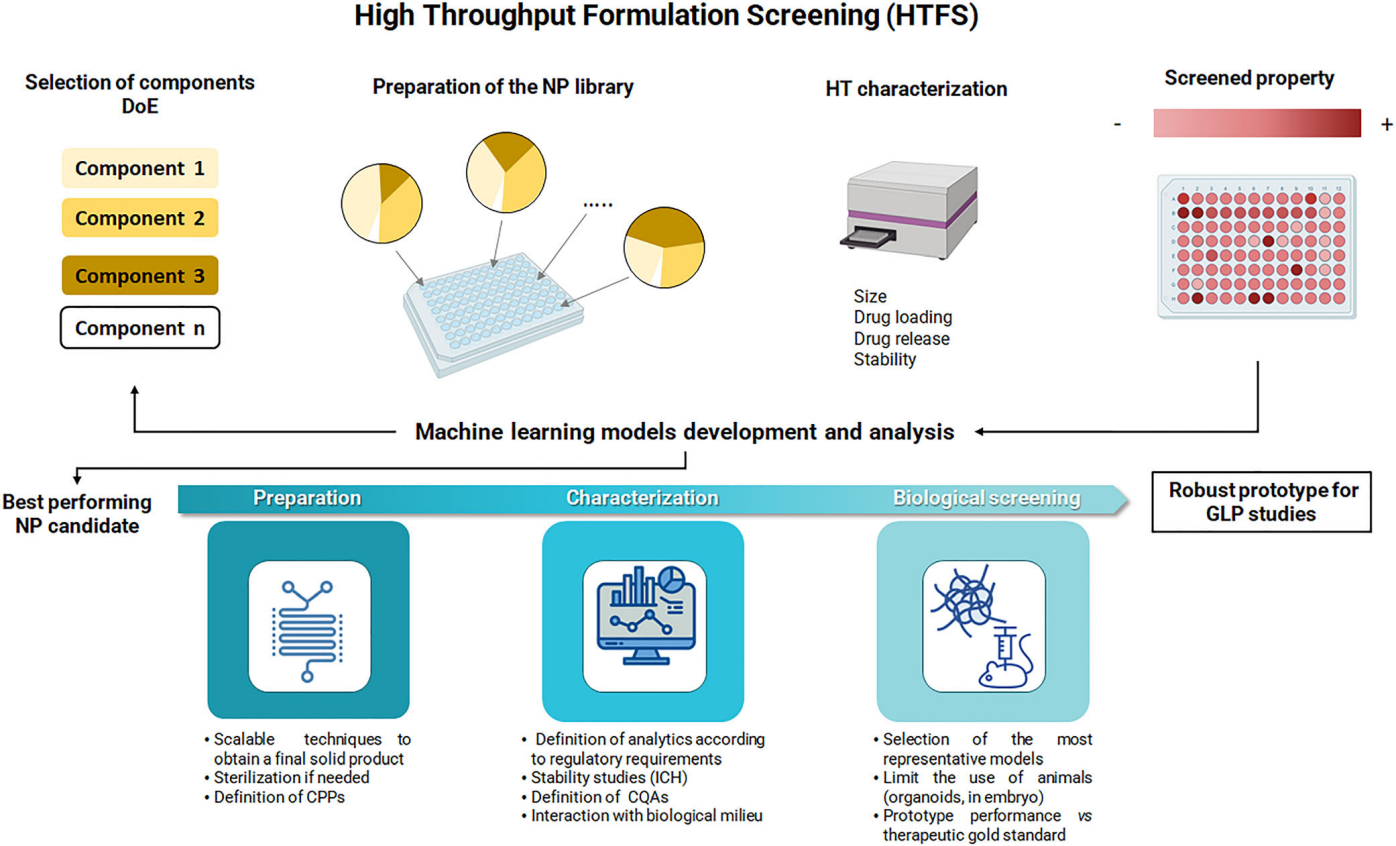
Schematic of the research workflow envisaged in the development of NPs. High throughput formulation screening and machine learning facilitate the selection of the best‐performing NP candidate. In the following step, the fabrication of the selected candidates, taking into account scale‐up and quality requirements in the early NP pipeline, ensures that only robust prototypes move to GLP studies.

Taken together, we conclude by proposing an overall flowchart of research and corresponding actions to implement in the preclinical phase (Figure 8) that, in our view, may push the development and clinical application of polyester NPs.

## AUTHOR CONTRIBUTIONS

**Giuseppe Longobardi:** Writing – original draft (supporting). **Thomas Lee Moore:** Data curation (lead); methodology (lead); writing – review and editing (supporting). **Claudia Conte:** Supervision (equal). **Francesca Ungaro:** Validation (equal). **Ronit Satchi‐Fainaro:** Writing – review and editing (equal). **Fabiana Quaglia:** Writing – review and editing (lead).

## CONFLICT OF INTEREST STATEMENT

The author has declared no conflicts of interest for this article.

## RELATED WIREs ARTICLE

Delivery of therapeutics with nanoparticles: What's new in cancer immunotherapy?

## Supporting information


**Data S1:** Supporting Information.

## Data Availability

Data sharing is not applicable to this article as no new data were created or analyzed in this study.
