# Supplementary material for: Polyester nanoparticles delivering chemotherapeutics: Learning from the past and looking to the future to enhance their clinical impact in tumor therapy
Source: Wiley Interdiscip Rev Nanomed Nanobiotechnol. 2024 Sep 1;16(5):e1990. doi: 10.1002/wnan.1990 (PMC11670051; doi:10.1002/wnan.1990)
Supplement: Supplementary file 1 — Data S1: Supporting Information. [file WNAN-16-e1990-s001.docx]

**Further Reading**

**Knowledge Mapping Methods**

For the scientific mapping approach, a search on terms related to polymeric nanoparticles and cancer/tumors was conducted via the Web of Science (WoS): <https://www.webofscience.com/wos/woscc/summary/3041a176-28c5-4a54-8f22-a2c8822f2bfb-cc22902a/relevance/1>, Accessed 12 February 2024. The specific terms of the search were:

("polymeric nanoparticles" OR "polymeric micelles" OR "polymeric nanocapsules" OR "polymeric nanomedicine")

AND

("Cancer*" OR "tumour*" OR "tumor*" OR "solid tumour*" OR "solid tumor*" OR "Chemotherapy" OR "chemotherapeutic*" OR "anticancer drug*")

AND

("poly(lactic-co-glycolic) acid" OR "poly(lactide-co-glycolide)" OR "polylactide" OR "poly(lactic acid)" OR "PLA*" OR "PLGA*" OR "polyglycolide" OR "poly(glycolic acid)" OR "PLLA*" OR "PDLLA*" OR "PDLA*" OR "poly(epsilon-caprolactone)" OR "poly(*-caprolactone)" OR "PCL*")

This initial search yielded 3964 articles. However, after closer inspection of the resulting search terms, it was noted that several manuscripts had no relation to polyester nanoparticles. The database was thus further cleaned and parsed using R [1], a statistics-centric language for computing and graphics. A search was run using the grep utility to identify those articles containing any mention of specific biodegradable polyesters in the abstract, namely:

PLGA, poly(lactide-co-glycolide), poly(lactic-co-glycolic acid), PLA, poly(lactic acid), polylactic acid, polylactide, poly(d,l lactide), poly(d,l lactic acid), poly(d,l-lactide), poly(l-lactide), PLLA, PDLA, PGA, poly(glycolic acid), polyglycolic acid, polyglycolide, PCL, polycaprolactone, poly-epsilon caprolactone, poly-epsilon-caprolactone, poly(e-caprolactone), poly(ε-caprolactone), poly(ε caprolactone)

To remove case-dependent string matching, abstract text was converted to lower case using the stringr library [2]. These articles were filtered to eliminate any replicated articles using the dplyr library [3]. Roughly 15% of the articles contained no mention in the abstract of any of the above-mentioned words.

In order to further understand which types of cancers are primarily being investigated for polymeric nanomedicine research, search results were filtered to include only articles (as the document type), and then an analysis of article abstracts was performed whereby a search was done through the abstract of each document for instances of words contained within the following list of “cancers:”

bladder cancer, breast carcinoma, breast cancer, brain cancer, bone cancer, bowel cancer, cervical cancer, cholangiocarcinoma, colon carcinoma, colorectal cancer, condrosarcoma, gastric cancer, glioblastoma, glioblastoma multiforme, hepatocarcinoma, hepatocellular carcinoma, hodgkin lymphoma, kaposi sarcoma, karposi's sarcoma, lewis lung carcinoma, lung cancer, liposarcoma, liver cancer, mammary carcinoma, medulloblastoma, melanoma, mesothelioma, neck cancer, head and neck cancer, head & neck cancer, neuroblastoma, non-hodgkin lymphoma, ovarian carcinoma, ovarian cancer, osteosarcoma, prostate cancer, pancreatic cancer, rectal cancer, retinoblastoma, rhabdomyosarcoma, skin cancer, stomach cancer, testicular cancer, thyroid cancer

Words within this list were extracted for each article (identified by the DOI), and duplicated mentions of words were trimmed by selecting unique instances per DOI. Then, the summary of each word/cancer instance was counted (i.e. that is how many articles contained an instance of each cancer). Cancers were roughly grouped by type (e.g. “glioblastoma” and “neuroblastoma” where grouped into “brain cancer”, etc.).

A similar search was done for therapeutics. A list of drug/therapeutic candidates was curated, and a fuzzy search within the abstract of each article was performed:

5-fluorouracil, 5-fu, 6-mercaptopurine, adriamycin, altretamine, azacitidine, bortezomib, busulfan, cabazitaxel, capecitabine, carboplatin, carmustine, cisplatin, clofarabine, colchicine, curcumin, cyclophosphamide, cyclosporine, cytarabine, dacarbazine, daunorubicin, docetaxel, dox, doxorubicin, epirubicin, erlotinib, etoposide, floxuridine, fludarabine, fluorouracil, gefitinib, gemcitabine, idarubicin, ifosfamide, imatinib, irinotecan, lapatinib, lomustine, melphalan, methotrexate, O6-benzylguanine, oxaliplatin, paclitaxel, pemetrexed, pentostatin, pralatrexate, quercetin, rapamycin, resveratrol, sirolimus, sorafenib, tamoxifen, taxol, temozolomide, teniposide, topotecan, trabectedin, trastuzumab, valrubicin, vinblastine, vincristine, vinorelbine

The resulting data frame was cleaned up to comprise of each therapeutic summarized by it’s frequency. During the cleaning process, abbreviations and synonyms were substituted for the full name of the therapeutic (e.g. “dox” and “adriamycin” became “doxorubicin”, “ptx” and “taxol” became “paclitaxel”, etc.).

**Overview of Publication Bibliometrics**

From the articles curated from the WoS search described above, an analysis of the bibliographic information was performed. Publications were grouped by the journal and the top number of citations (for all manuscripts combined) was calculated. **Figure S1 A** shows the number of citations for the top 20 cited journals broken down by article type (e.g. Article or Review). These journals were further stratified by the number of publications per journal (**Figure S1 B**) and the median number of citations per article (**Figure S1 C**). It is important to note that the median number of citations per article may be highly dependent on the number of articles per journal. For example, if a journal has a single relevant article that has been cited thousands of times, this “median citations per article” value will be highly skewed by this difference.

**Figure S1 D** shows the breakdown of the search results into article types, with 82% of the 3368 search results being original research articles and 18% being reviews. The number publication over time also shows the growth of the field from the initial publications in the late 1990s until the 2020s, with a peak in 2018 (**Figure S1 E**). An analysis of the journals’ research areas shows the breakdown of the journal-specific fields divided between the original articles and reviews (**Figure S1 F**).


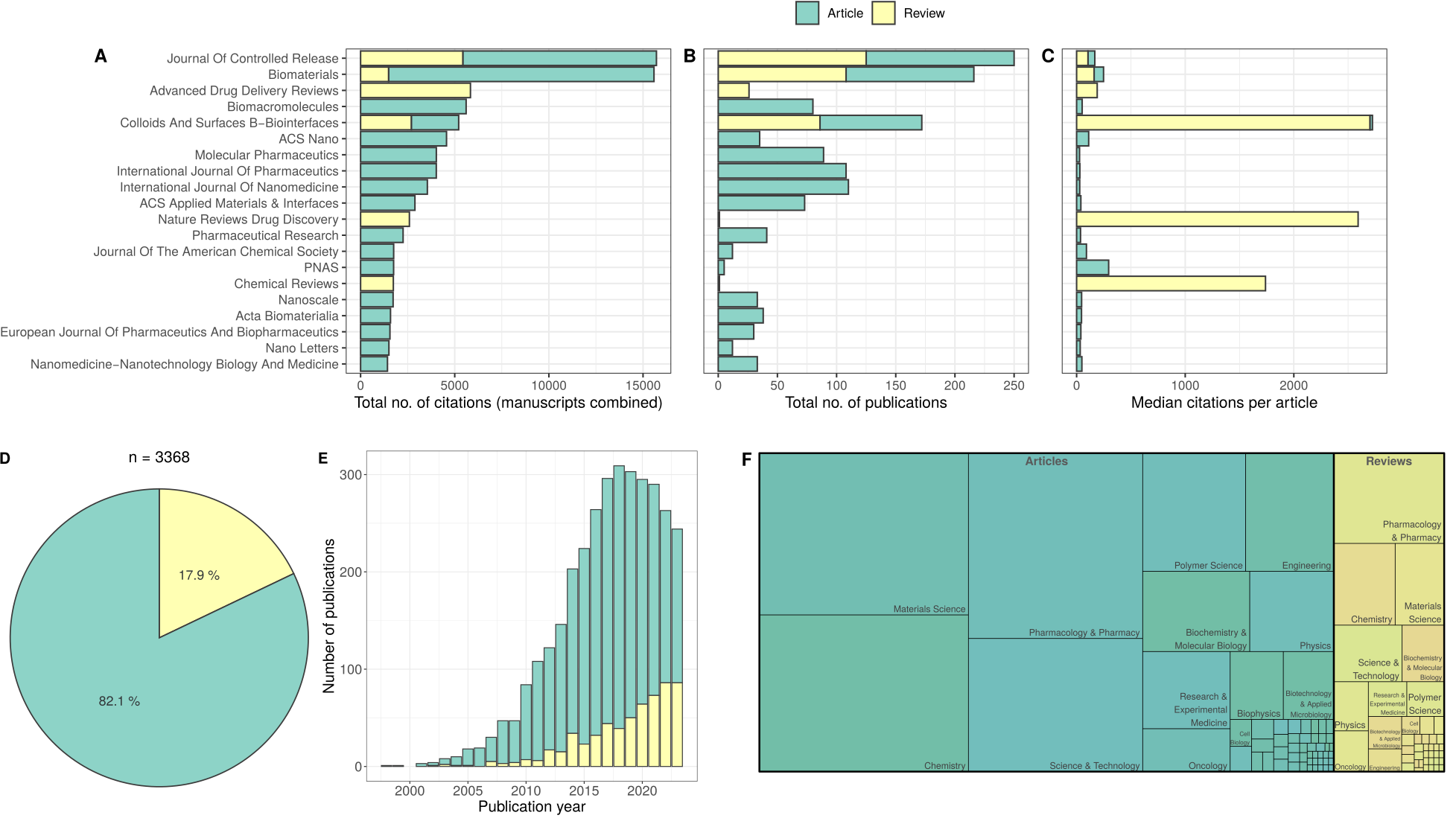
**Figure S1.** Bibliometric analysis of the Web of Science (WoS) search results. The search returned 3368 articles (reviews and original research articles). **(A)** Grouping the results by journal and summing the total number of citations reveals the top 20 journals publishing in this area. These results can further be evaluated based on **(B)** the number of publications per journal and **(C)** the median number of citations per article. **(D)** Overall, the WoS search results could be broken down into ~80% articles and ~20% reviews, and **(E)** the number of articles/reviews published per year. **(F)** An analysis of the journals reveals the top subject areas of these journals for both articles and reviews.

**References**

1. R Core Team (2021). R: A language and environment for statistical computing. R Foundation for Statistical Computing, Vienna, Austria. URL https://www.R-project.org/
2. Hadley Wickham (2023). stringr: Simple, consistent wrapper for common string operations. R package version 1.5.1, URL <https://CRAN.R-project.org/package=stringr>
3. Hadley Wickham, Romain François, Lionel Henry, Kirill Müller, Davis Vaughan (2023). dplyr: A grammar of data manipulation. R package version 1.1.4, URL https://CRAN.R-project.org/package=dplyr
